# A Fine-Tuned Lipophilicity/Hydrophilicity Ratio Governs Antibacterial Potency and Selectivity of Bifurcated Halogen Bond-Forming NBTIs

**DOI:** 10.3390/antibiotics10070862

**Published:** 2021-07-15

**Authors:** Anja Kolarič, Maja Kokot, Martina Hrast, Matjaž Weiss, Irena Zdovc, Jurij Trontelj, Simon Žakelj, Marko Anderluh, Nikola Minovski

**Affiliations:** 1 Laboratory for Cheminformatics, Theory Department, National Institute of Chemistry, Hajdrihova 19, SI-1001 Ljubljana, Slovenia; anja.kolaric2@um.si (A.K.); maja.kokot@ki.si (M.K.); 2 Pharmaceutical Chemistry, Faculty of Pharmacy, University of Ljubljana, Aškerčeva cesta 7, SI-1000 Ljubljana, Slovenia; martina.hrast@ffa.uni-lj.si (M.H.); matjaz.weiss@ffa.uni-lj.si (M.W.); 3 Institute of Microbiology and Parasitology, Veterinary Faculty, University of Ljubljana, Gerbičeva 60, SI-1000 Ljubljana, Slovenia; irena.zdovc@vf.uni-lj.si; 4 Biopharmaceutics and Pharmacokinetics, Faculty of Pharmacy, University of Ljubljana, Aškerčeva cesta 7, SI-1000 Ljubljana, Slovenia; jurij.trontelj@ffa.uni-lj.si (J.T.); simon.zakelj@ffa.uni-lj.si (S.Ž.)

**Keywords:** antibacterials, drug discovery, DNA gyrase inhibitors, topoisomerase IV inhibitors, intercalators, novel bacterial topoisomerase inhibitors

## Abstract

Herein, we report the design of a focused library of novel bacterial topoisomerase inhibitors (NBTIs) based on innovative mainly monocyclic right-hand side fragments active against DNA gyrase and Topo IV. They exhibit a very potent and wide range of antibacterial activity, even against some of the most concerning hard-to-treat pathogens for which new antibacterials are urgently needed, as reported by the WHO and CDC. NBTIs enzyme activity and whole cell potency seems to depend on the fine-tuned lipophilicity/hydrophilicity ratio that governs the permeability of those compounds through the bacterial membranes. Lipophilicity of NBTIs is apparently optimal for passing through the membrane of Gram-positive bacteria, but the higher, although not excessive lipophilicity and suitable hydrophilicity seems to determine the passage through Gram-negative bacterial membranes. However, due to the considerable hERG inhibition, which is still at least two orders of magnitude away from MICs, continued optimization is required to realize their full potential.

## 1. Introduction

The loss of effective antibiotics is undermining our ability to fight bacterial infectious diseases and managing the infectious complications that were considered treatable not long ago. Life-threatening multi-drug resistant bacterial infections are notably common in hospital environments, where patients with compromised immune systems are significantly exposed and portray a particularly vulnerable risk group. In 2008 ESKAPE (*Enterococcus faecium*, *Staphylococcus aureus*, *Klebsiella pneumoniae*, *Acinetobacter baumannii*, *Pseudomonas aeruginosa*, and *Enterobacter species*) pathogens [1] were introduced as extremely concerning bugs in hospitals due to their escalating resistance to many effective antimicrobial agents. Similarly, in 2013 Centers for Disease Control and Prevention (CDC) presented antibiotic resistance threats report, dividing them in three categories, urgent, serious, and concerning based on the antibiotic resistance threats in the United States [2]. In 2017 the World Health Organization (WHO) announced, for the first time, the antibiotic resistance priority list, which became a global R&D guidance explicitly focused on specific pathogens; critical, high, and medium priority pathogen categories were defined, ranking them according to the urgency of need for new antibacterials [3]. “Critical” pathogens group include *A. baumannii*, *P. aeruginosa*, and *Enterobacteriaceae* species (e.g., *Escherichia coli* and *K. pneumoniae*), which are posing particular threat among hospitalized patients with mostly fatal infections such as sepsis and pneumonia. Several other bacterial strains like methicillin-resistant *S. aureus* (MRSA) have been included in the “High” priority list, but they pose no less threat to human health than bacterial strains from the “Critical” list. The only difference is probably few more years that we have to find agents that are effective against the “High” priority group, while for the “Critical” group alarm clock is already ringing—some strains do not respond to any known therapeutic antibacterial agent [4]. Similar conclusions were also drawn for the latest list of prioritized bacterial pathogens released in 2019 by CDC, updating urgent, serious, and concerning bacteria that require attention now, because the antibiotic resistance is still spreading [5]. Accordingly, a global concern was raised, emphasizing the necessity for new classes of antibacterials.

**Figure 1:**
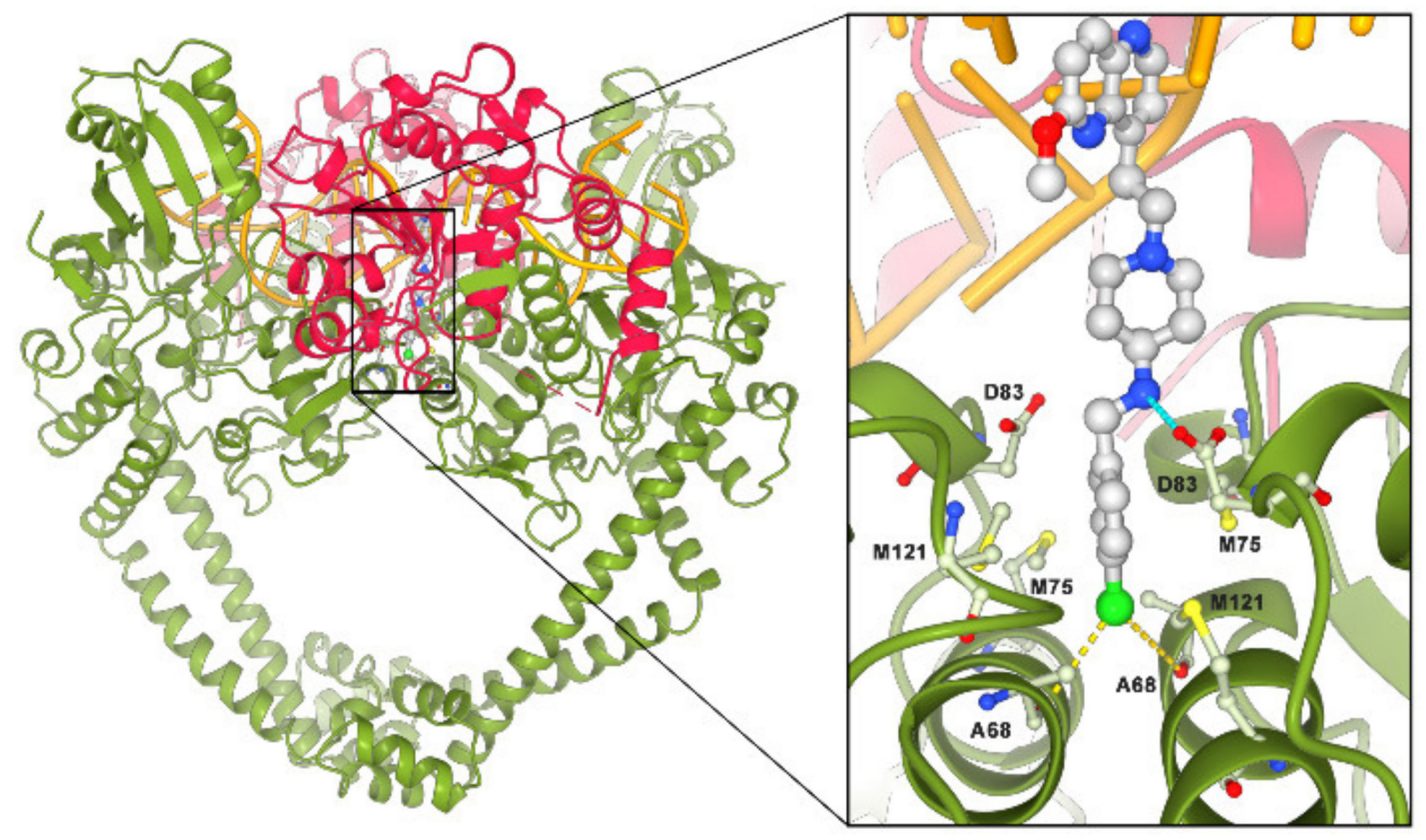
Binding mode representation of an NBTI ligand (inset, grey, compound **13**) in a complex with *S. aureus* DNA gyrase and DNA molecule (PDB ID: 6Z1A) [13]. The GyrA subunit is shown in green, the gyrase B (GyrB) subunit is red, while the DNA molecule is orange. Ionic interaction is shown as cyan dots, while the bifurcated symmetrical halogen bonds are represented as yellow dots.

In response to this global campaign, novel bacterial topoisomerase inhibitors (NBTIs) have become a highly promising class of new antibacterial agents active against well-validated antibacterial targets, the DNA gyrase and its paralogous form topoisomerase IV (Topo IV) [6,7,8,9,10]. By forming a ternary complex with the bacterial enzyme and DNA they prevent native enzyme function that consequently ends with bacterial cell death [9]. Contrary to the well-established quinolones, NBTIs bind to an alternative binding site thereby avoiding the cross-resistance [9]. They are comprised of three major parts; (1) a left-hand side (LHS) that intercalates between DNA base pairs and stabilizes only single-strand cleavage breaks, which is connected through (2) a linker to (3) the right-hand side (RHS) fragment that binds in a pocket midway between both gyrase A (GyrA) subunits [10]. The secondary amine on linker’s chain was identified as an essential element for activity making a contact with Asp83 at the entrance of GyrA hydrophobic binding pocket (Figure 1) [9,10,11,12,13]. The formation of complexes with single-strand DNA breaks has been recognized as a mechanism of inhibition of the enzyme function. We recently solved a 2.3-Å crystal structure of compound **13** in complex with *S. aureus* DNA gyrase and DNA (PDB ID: 6Z1A, Figure 1) [13]. It demonstrates for the first time a co-crystallized inhibitor in a single orientation that does not suffer from static disorder as all previously reported gyrase crystal structures complexed with NBTIs [14]. Due to crystallization of the inhibitor in a single orientation, we were able to relate the compound orientation with the conformation of the catalytic pocket, which allowed us to demonstrate the course of stabilization mechanism. The intercalation of the LHS fragment between DNA base pairs induce a specific spatial rotation that is mandatory for accomplishing optimal stacking interactions. In a concomitant fashion, this rotation affects ribose and phosphates, as well that brings scissile phosphate in one catalytic pocket closer to the catalytic metal and enables catalytic activity, while in the second catalytic pocket scissile phosphate moves away from the catalytic metal and deactivates any catalytic activity. Consequently, DNA breaks can only occur in one strand [13,14].

Notwithstanding the excellent antibacterial properties of existing NBTIs published in the literature, a major obstacle for their progression into clinical trials and more importantly appearance on the market lies in their hERG toxicity that is near to potential therapeutic concentrations. The endeavors to minimize this hERG-related cardiotoxic potential have been particularly invested through modification of NBTIs physicochemical properties, such as basicity and lipophilicity. Such approaches led to noticeable hERG improvements, but unfortunately in many cases at the expense of lowering their antibacterial activity [15]. However, some recent reports have shown that it is possible to improve hERG cardiotoxicity and maintain good antibacterial activity despite reduced basicity [16,17]. This prompted us to focus on optimizing NBTIs in increasing their antibacterial potency across the bacterial spectrum to provide acceptable therapeutic window even with observed hERG toxicity. Namely, increasing antibacterial potency can be achieved either by increasing potency on isolated enzyme or by balanced activity on two related enzymes (e.g., DNA gyrase and Topo IV). By utilizing structural and physicochemical property optimizations, we have managed to obtain among the best-in-class NBTIs remarkably active against a panel of Gram-positive and some Gram-negative bacterial pathogens from the WHOs “Critical” and “High” priority lists with a high potential for further progress.

## 2. Results and Discussion

### 2.1. NBTIs Design Strategy

**Figure 2:**
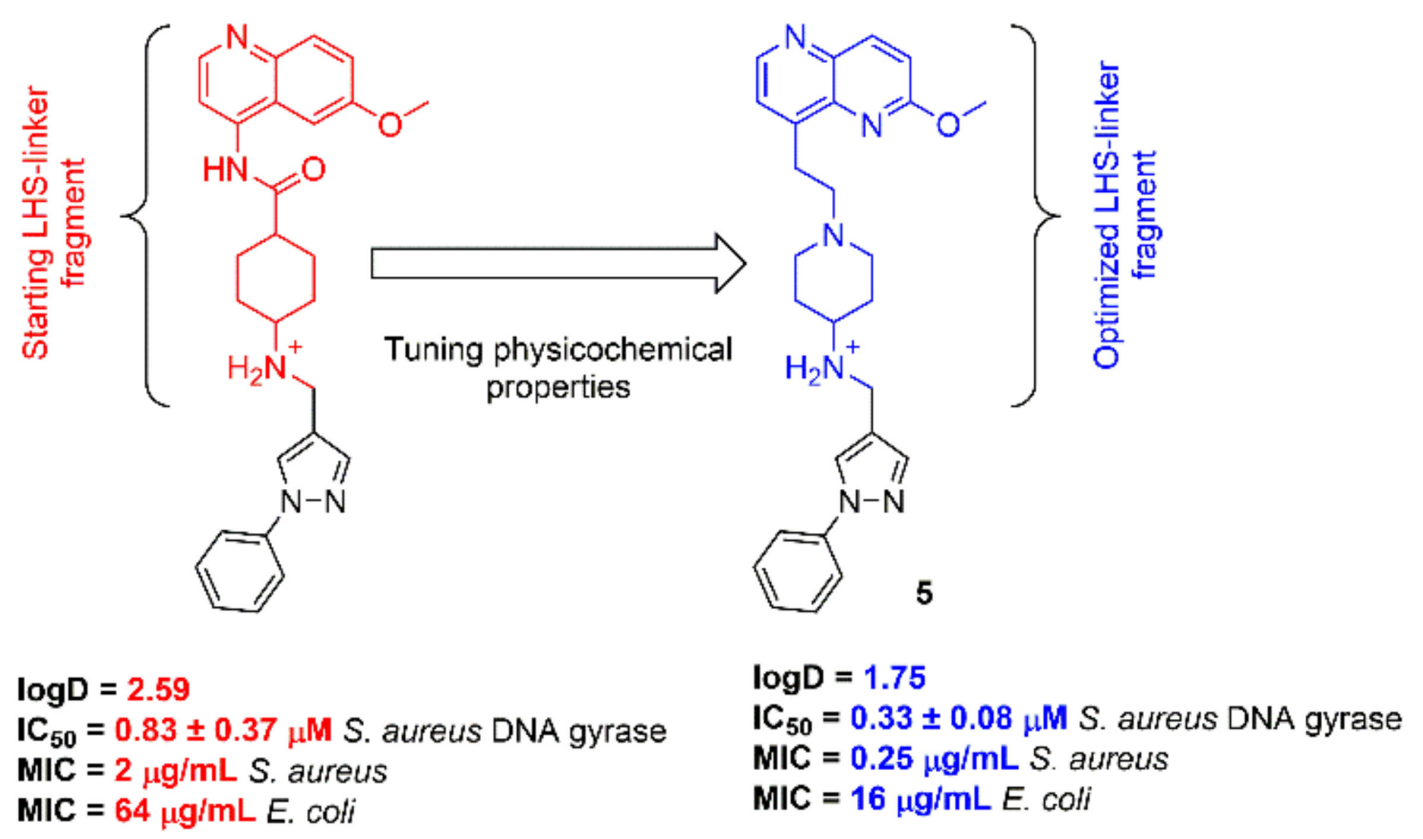
The impact of lipophilicity (logD) and structural flexibility on the change in IC_50_ for a compound containing methoxy-quinoline cyclohexyl-amide LHS-linker combination against *S. aureus* DNA gyrase from our previous study (**left**) [18] and compound **5** (**right**) comprising naphthyridine aminopiperidine LHS-linker construct confirming the rationally optimized physicochemical properties and reduced rigidity (amide vs. ethylene) leading to the improved potency and antibacterial activity.

The strategy for designing optimized NBTI compounds was substantiated on our previous study [18], where we initially synthesized a tiny series of five NBTIs with innovative RHS moieties active against *S. aureus* DNA gyrase [18,19]. Due to their inadequate solubility, excessive rigidity, and instability (compounds were disintegrating), these compounds failed to outperform reported NBTIs in terms of both enzyme inhibition and antibacterial potency [18]. In the present study, the first optimization step was aimed at increasing overall compounds solubility by replacing previously used methoxy-quinoline LHS with a more optimal, naphthyridine LHS as well as the cyclohexyl-amide linker with aminopiperidine linker (Figure 2); these structural alterations introduce a higher flexibility and ensure a proper spatial arrangement of the LHS and RHS, respectively [9]. By retaining identical RHS fragments from our previous study [18], compounds **3**–**7** were synthesized (Table 1). Replacement of LHS-linker construct contributed in increasing the overall polarity (i.e., lowered logD, from 2.59 to 1.75, Figure 2) as well as the basicity (an additional basic center) indicating an improved compounds solubility and unexpectedly increased stability. To our surprise, compound **5** showed 2.5-fold improvements in *S. aureus* gyrase IC_50_ and higher antibacterial potency compared to its predecessor. Since, adjusting physicochemical properties (i.e., lowering logD and increasing pKa) was recognized as an appropriate step toward enhancing NBTIs permeation across membranes of Gram-negative bacteria [20], we envisaged it as an appropriate design feature for improving the whole-cell activity, as well.

The solubility of all studied compounds (Supplementary Materials Table S1) was evaluated by determining the maximum single-dose, which could still dissolve in 500 mL of the fluid assumed to be available for dissolution in the intestinal tract and thereby presenting a limit for drug development [21] (Supplementary Materials Table S1). The most restrictive standard pH (6.8) for basic compounds was chosen for this evaluation. Especially the compounds **9**, **10**, and **16** appear to be soluble at this pH in any conceivable single-dose, while compounds **8**, **13**–**15**, **19**, and **20** would require either dosing below 100 mg or formulation aided solubilization. It is also worth noting that enhanced solubility could also be expected in the more acidic conditions of the stomach, which combined with the high permeability is still favorable for absorption in the upper small intestine. The PAMPA permeability of all tested compounds except **7**, **16**, and **17** is comparable to that of highly permeable reference drugs and clearly higher than that of poorly permeable reference drugs [22].

**Figure 3:**
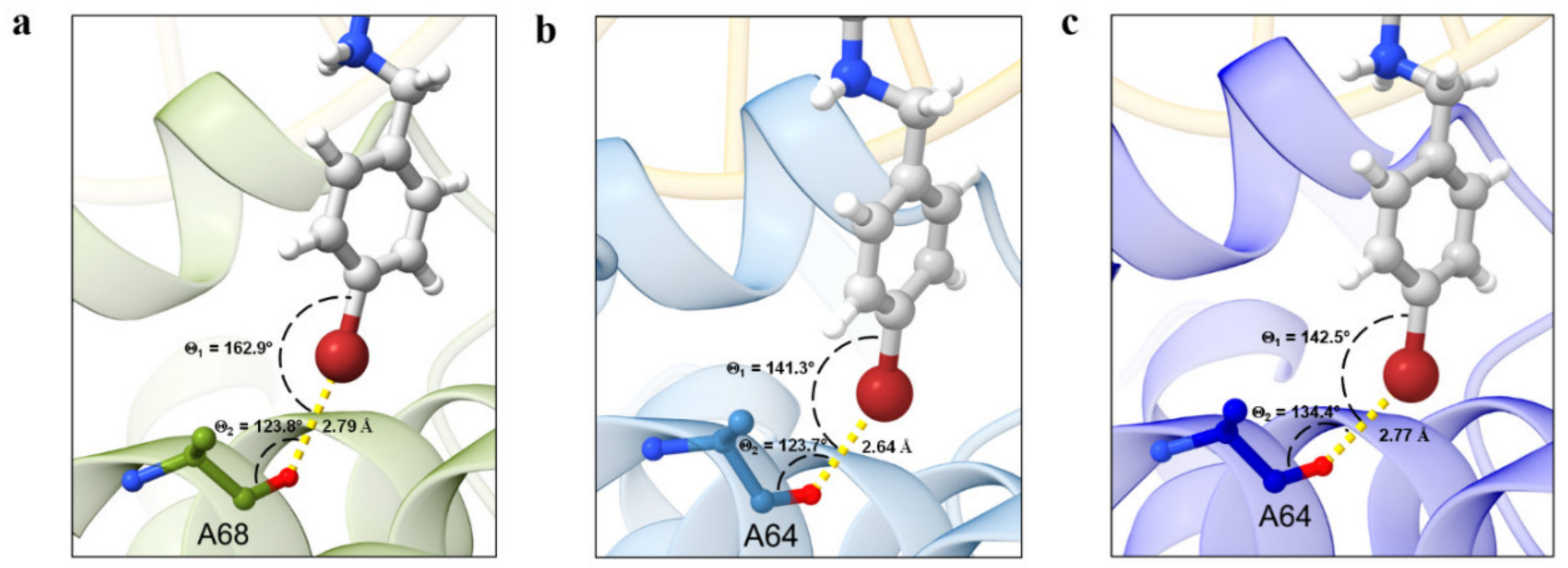
Halogen-bonding interactions for compound **14** as predicted by flexible molecular docking calculations within the RHS binding site of: (**a**) *S. aureus* DNA gyrase (PDB ID: 2XCS) [9], (**b**) *S. aureus* Topo IV homology model, and (**c**) *E. coli* Topo IV homology model. Enzymes are shown in green (DNA gyrase) and blue (Topo IV) ribbons, respectively, compound **14** is colored by element (ball and sticks representation), while DNA in pale orange. Halogen-bonding interactions are represented as yellow dots.

Once proper physicochemical properties of LHS-linker fragment were achieved (Supplementary Materials Table S1), we focused on increasing target affinity (GyrA only at the beginning) and consequently antibacterial potency. Considering that the structure–activity relationship (SAR) of such LHS-linker fragment is relatively well established [10], we recognized a major potential for antibacterial activity improvement in amplifying their binding to DNA gyrase through applying various RHS alterations. While LHS is responsible for DNA intercalation, RHS rules GyrA binding, and consequently influences the overall NBTIs binding affinity and inhibitory potency. After a careful review of the most prosperous NBTIs from the literature [9,10,12,23], with a special focus on their RHS moieties, we observed that the majority of NBTIs contain rigid bicyclic RHSs. In contrast to them, our compound **5** comprises a more flexible 1-phenyl-1*H*-pyrazole RHS moiety, which was identified as the most active one (*S. aureus* DNA gyrase IC_50_ = 0.33 μM in Figure 2) among compounds **3**–**7** (Table 1); consequently, we directed our improvement strategy toward introducing more flexible RHSs. By comparing the length of RHS fragments in terms of measuring the interatomic distances between linkers’ secondary amine and the geometrically most distant RHS heavy atom (Supplementary Materials Table S2), this distance was found to be more than 8 Å for our flexible RHSs (compounds **3**–**8**), comparing to ~7 Å or less for known bicyclic RHSs. Our further optimization goal was thus directed to slight shortening the RHS length that was achieved by introducing monocyclic moieties preferably substituted on *para*-position with aliphatic groups (compounds **9**–**22**), thereby maintaining some degree of flexibility. Such RHSs would enable better adaptation into gyrase binding pocket (Figure 3, Supplementary Materials Figures S1 and S2). The majority of known RHS fragments form mainly van der Waals interactions with the RHS-binding pocket delineated with hydrophobic amino acid residues (i.e., Ala68, Gly72, Met75, Met121 for *S. aureus* gyrase). On the contrary, our idea for strengthening NBTIs binding and consequently improving their overall antibacterial activity was substantiated on targeting the backbone carbonyl oxygens of Ala68 residues from both GyrA subunits with *p*-substituted RHSs. A similar approach was already previously used in achieving excellent enzyme inhibitory activity in the discovery of GSK299423, an NBTI capable to establish unusual hydrogen bonds between the methylene group of the oxathiolopyridine RHS and both Ala68 backbone carbonyl oxygens [9,13]. Apart from important interactions, the added benefit of targeting backbone carbonyls lies in their presumably lower susceptibility to mutations. Although this cannot be stated without a reasonable doubt, very few mutations, if any, may result in backbone cabonyls’ eradication, and few may mask their availability due to steric hindrance, making them less likely to contribute to developing NBTIs acquired resistance. Therefore, monocyclic *p*-phenyl RHSs were substituted with various groups (compounds **12**–**17**) that would strengthen the binding by hydrogen (-CH2OH, -CONH2) or halogen (F, Cl, Br, I) bonding with the backbone carbonyl oxygen of at least one of Ala68 or other non-bonded interactions. We have predicted the possibility of such hydrogen/halogen bonds formation by flexible molecular docking calculations within NBTIs gyrase binding site of *S. aureus* (PDB ID: 2XCS) [9]. The design of compounds and prediction of their ability to form such strong interactions was described in detail in our recent study [13]. Since, NBTIs act on both Gram-positive and Gram-negative bacteria, the ability of such hydrogen/halogen bond interactions was predicted by flexible molecular docking calculations within NBTIs binding pocket of a constructed *E. coli* gyrase homology model, as well [18]. Furthermore, Topo IV is a secondary [11] and equivalently important NBTI target to DNA gyrase, therefore a balanced inhibition of both targets would be advantageous in achieving more potent antibacterial activity and broad spectrum. Since, the enzymes do not act synergistically [24], a balanced activity would also be an advantage in preventing development of bacterial resistance through maintaining activity in one enzyme target in the event of mutations in another. To predict how the compounds would bind to Topo IV and which interactions they may form (especially interactions with the backbone carbonyl oxygen of corresponding Ala64), we also performed flexible molecular docking calculations within NBTI binding site of our *S. aureus* and *E. coli* Topo IV assembled homology models, respectively. The predicted binding mode of *p*-halogenated compounds (Supplementary Materials Figures S1 and S2) is exemplified by compound **14** (Figure 3). Taking into account the defined X···O bond lengths (Cl···O < 3.27 Å, Br···O < 3.37 Å, I···O < 3.50 Å) and angles (140° ≤ Θ_1_ [C-X···O] ≤ 180° and 90° ≤ Θ_2_ [X···O = C] ~ 120°) [25], a high probability of halogen bond formation with the backbone carbonyl oxygen of Ala68 residue from one GyrA subunit of *S. aureus* was noticed (Figure 3a, Supplementary Materials Figure S1a,c,e). Likewise, halogen-bonding was predicted for *S. aureus* and *E. coli* Topo IV with the backbone carbonyl oxygen of the corresponding Ala64 from one ParC subunit (Figure 3b,c and Supplementary Materials Figure S2). Surprisingly, no halogen or any other interactions with the corresponding alanine (Ala67) was noticed in the case of *E. coli* DNA gyrase (Supplementary Materials Figure S1b,d,f), and no hydrogen bonds were anticipated for any of the investigated NBTIs.

### 2.2. Synthesis

**Scheme 1:**
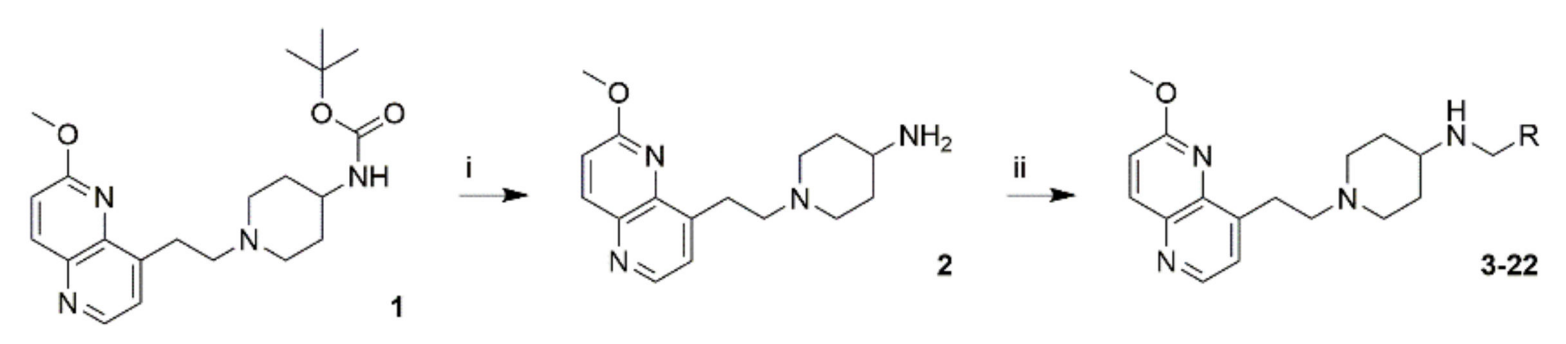
Synthesis of final compounds **3**–**22**. Reagents and conditions: (i) 1M HCl in CH_3_COOH, 30 min, rt; (ii) RCHO, MeOH, CH_3_COOH, 2 h, then NaCNBH_3_, 0 °C, 10 min, then rt.

The free amine **2** was provided by Boc deprotection of commercially available compound **1** (DSK-1030, DSK InnoSciences Pvt. Ltd., Hosur, Tamil Nadu, India) with 1M HCl in acetic acid. All final compounds **3**–**22** were obtained by one-step reductive amination using various aldehydes and NaCNBH_3_ as a reducing agent, as shown on the Scheme 1.

### 2.3. In Vitro Measurement of Inhibitor Potency and Antibacterial Activity

The enzyme inhibition profile of optimized NBTI compounds was assessed by *in vitro* enzyme inhibition assay on isolated bacterial targets, DNA gyrase and Topo IV, originating from wild-type *S. aureus* and *E. coli*, respectively. *In vitro* antibacterial susceptibility expressed as minimal inhibitory concentration (MIC) values was determined against a panel of Gram-positive (*S. aureus*, methicillin-resistant *S. aureus*, *Streptococcus agalactiae*, *Enterococcus faecalis*, and *Streptococcus pneumoniae*), Gram-negative (*E. coli*, *Salmonella alachua*, *P. aeruginosa*, *A. baumannii*, and *Campylobacter jejuni*), and *Mycobacterium* strains (*Mycobacterium smegmatis* and *Mycobacterium avium*), enriched by various MRSA and *E. coli* strains, resistant to several antibiotics as well as *E. coli* strains with increased membrane permeability and lack of efflux pump, as well. Moreover, selectivity of the compounds for bacterial enzymes over orthologous human topoisomerase IIα was also assessed.

As displayed in Table 1, the compounds exhibit stronger enzymatic inhibition (lower IC_50_s) against *S. aureus* DNA gyrase and *E. coli* Topo IV compared to *E. coli* DNA gyrase and *S. aureus* Topo IV. Compounds **14** and **15** with the most potent IC_50_ values on all enzymes come closest to the balanced inhibition; however a difference of 1–2 orders of magnitude in enzyme inhibitory activity (of the same bacteria) is still too high to provide desired balanced DNA gyrase/Topo IV inhibition profile. All compounds expressed selectivity for bacterial enzymes over the orthologous human topoisomerase IIα enzyme, with **15** achieving approximately 1000-fold higher selectivity for bacterial enzyme.

Antibacterial activity is presented in Table 2. The potencies against all MRSA strains were rather comparable for each specific compound. Compounds with bicyclic RHS moiety, such as **6** and **7** were inactive against all strains, while compound **3** showed moderate inhibition and antibacterial potency of *S. aureus* and other Gram-positive strains but was generally poorly active against Gram-negatives. Compounds **4** and **5** comprising 1-phenylpyrazole RHS inhibited *S. aureus* DNA gyrase and *E. coli* Topo IV enzyme, with higher potency on *E. coli* Topo IV (0.33 µM and 0.09 μM, respectively). The overall antibacterial profile of compound **5** with unsubstituted phenyl RHS was found to be more favorable than its fluoro analog **4**; therefore, it represented a starting point for further optimization. The same trends were also observed in our previous study for compounds containing the same RHS fragments in combination with methoxy-quinoline cyclohexyl-amide LHS-linker fragment [18]. The addition of another nitrogen into pyrazole ring (**8**) resulted in slightly decreased potency against Gram-negative strains. According to our design hypothesis, shorter and more flexible RHS fragments, such as isopropyl (**9**) and allyl (**10**) chains were introduced into pyrazole ring. The shorter isopropyl group (**9**) improved *S. aureus* gyrase IC_50_ (0.16 μM) compared to **5** (0.33 μM), while allyl (**10**) group reduced gyrase inhibition and antibacterial activity against all strains, even 8-fold for *S. aureus*, *E. faecalis*, and *A. baumannii*. Both RHS fragments decreased Topo IV enzyme activity.

As a further optimization step, the substituted heteroaromatic pyrazole was replaced with phenyl to optimize the ligand efficiency (LE, Table 3) for their targets, *S. aureus* and *E. coli* DNA gyrase/Topo IV. Unsubstituted phenyl (**11**) showed considerable reduction of enzymatic inhibition as well as lack of antibacterial potency against all strains (up to 32-fold) compared to compounds with pyrazole RHS. However, a notable optimization was achieved by the introduction of *p*-halogenated phenyl RHSs (**12**–**15**), resulting in a significant improvement of DNA gyrase inhibition. This offered an initial confirmation that the predicted halogen bonds were most probably established and was in accordance with our intentions to obtain acceptable value of LE > ~0.3 [26]. Substantially lower LE values were observed for compounds **12**–**15** for *E. coli* DNA gyrase and *S. aureus* Topo IV in comparison to *S. aureus* DNA gyrase and *E. coli* Topo IV, which correlate with observed enzymes activities. Compound **12** with *p*-fluorophenyl RHS enhanced inhibitory activity and potency comparable to **4**, which contains the same fragment. *p*-Chlorophenyl substitution (**13**) led to remarkable inhibition improvement of all enzymes, which was evident in overall antibacterial spectrum. Furthermore, introduction of a bromine (**14**) and an iodine (**15**) showed an exceptional enzymatic inhibitory performances with IC_50_ of 0.007 μM and 0.011 μM for *S. aureus* DNA gyrase, which resulted in highest LE for all enzyme targets among the compounds. Considering that on-target activity increases with increasing the size of halogen, thereby also increasing lipophilicity (calculated values of clogP and logD are available in the Supplementary Materials, Table S1), which challenges our explanation that bifurcated halogen-bonding guide the enzyme inhibitory activity of presented NBTIs. Although, increasing halogen size certainly increases van der Waals forces, the highest difference in IC_50_ values in the series **11**–**15** is between *p*-fluoro (**12**) and *p*-chloro (**13**) derivatives on all enzyme targets, while modest increase in potency is seen for *p*-bromo (**14**) and *p*-iodo (**15**) derivatives. *p*-Fluoro substituent does not form halogen bonds, while *p*-chloro, *p*-bromo, and *p*-iodo does, which offers a reasonable confirmation that halogen-bonding is indeed taking place and contribute to the inhibitory potency. Nevertheless, enhancing the lipophilicity of compounds **11**–**15** increases their cell permeability (see results of PAMPA permeability in Supplementary Materials, Table S1), which most likely results in stronger whole cell activity.

**Figure 4:**
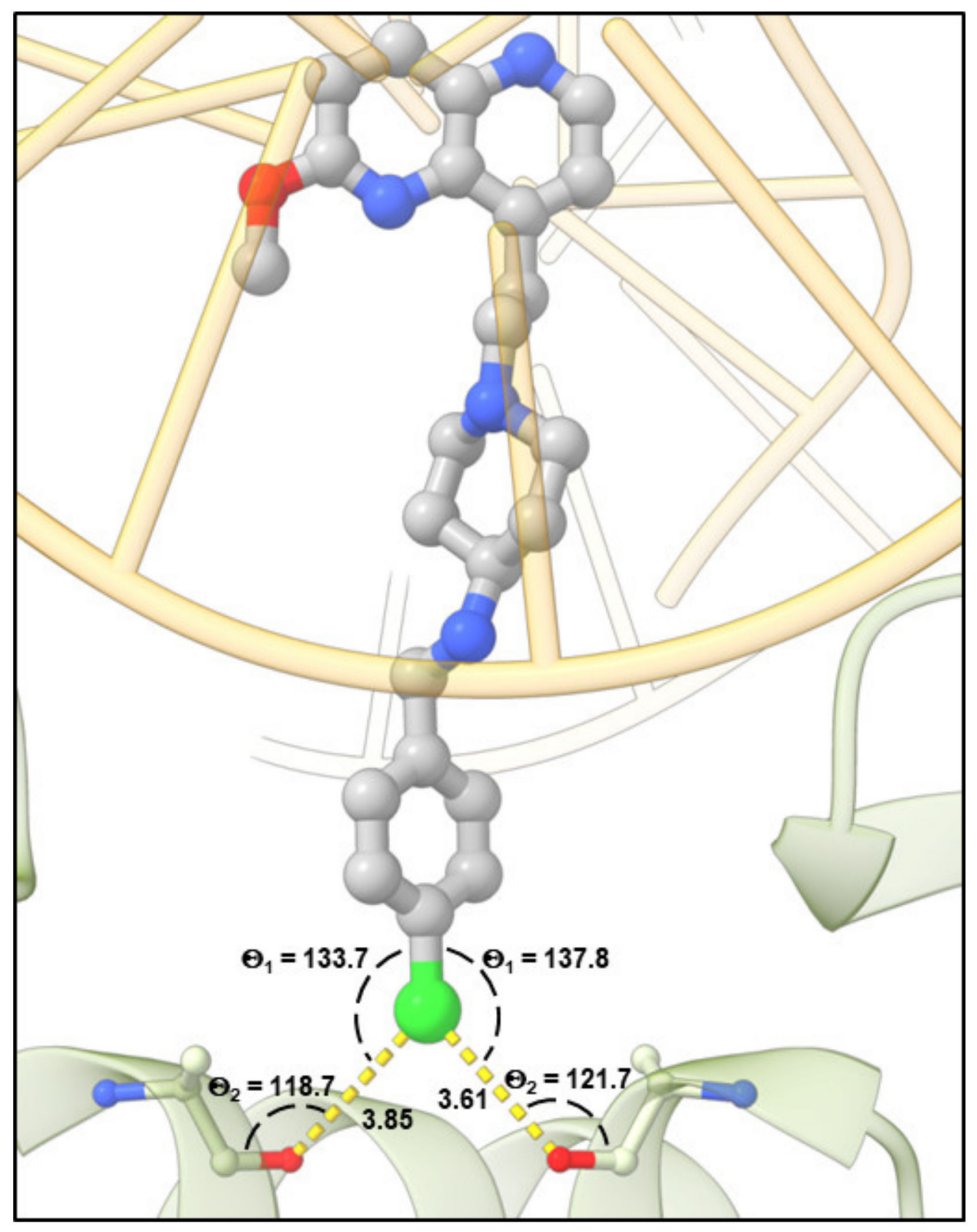
Symmetrical bifurcated halogen-bonding of compound **13** in complex with *S. aureus* DNA gyrase and DNA molecule (PDB ID: 6Z1A) [13]. GyrA subunit is shown in green, compound **13** is colored in grey (ball and sticks representation) and DNA in pale orange. Halogen bonds are represented as yellow dots.

A comparably low nanomolar inhibitory activity has been detected only for GSK299423 (IC_50_ = 0.014 ± 5 μM) [9], however such an outstanding MIC of 0.0078 μg/mL against various *S. aureus* strains for **15** is comparable or even better comparing to the reported NBTIs. Although, both **14** and **15** showed comparable enzymatic inhibition, which is probably due to steric hindrance effect of the iodine atom, the difference in antibacterial potency against all strains is not negligible. **15** shows generally 4-fold higher overall potency than **14** and remarkable MRSA and *S. pneumoniae* impact (MIC = 0.0078 μg/mL and 0.015 μg/mL, respectively), so it was identified as the most active and promising compound among the series. Reasonably potent antibacterial activity against other Gram-negative strains, *A. baumannii* (MIC = 0.25 μg/mL), *C. jejuni* (MIC = 0.015 μg/mL), and a moderate activity against *P. aeruginosa* (MIC = 32 μg/mL) was detected, as well. Moreover, **15** also exhibited an excellent *M. smegmatis* and moderate *M. avium* antibacterial activity with MIC values of 0.015 μg/mL and 1 μg/mL, respectively. This trend in increase of overall antibacterial performance with halogen size (H < F < Cl < Br < I) is as predicted by molecular docking calculations due to halogen bond formation, which was confirmed in our recently solved crystal structure of *p*-chloro compound **13** in a complex with *S. aureus* DNA gyrase and DNA (PDB ID: 6Z1A, Figure 1; Figure 4) [13] in which *p*-chlorophenyl RHS moiety is bound in the hydrophobic pocket formed by both GyrA subunits, delineated with amino acid residues Ala68, Gly72, Met75, and Met121. The most interesting feature related to our crystal structure is the formation of not a single one, but symmetrical bifurcated halogen bond between *p*-chloro and the backbone carbonyls of Ala68 residues from both GyrA subunits. Such halogen-bonding has not been previously identified in a biological system between a ligand and its macromolecular target and, moreover, it also contradicts the classical interpretation for angles and distance of halogen bonds. Detailed description of the crystal structure was described in our previous work [13].

Important conclusions can be drawn when comparing potency of **15** with Gepotidacin, an NBTI in phase III clinical trials for a potential first-in-class antibiotic. **15** outperforms Gepotidacin in isolated enzyme inhibition assay on virtually all four enzymes tested (Table 1). The more potent on-target activity results in more than two orders of magnitude more potent antibacterial activity on *S. aureus* (WT) while retaining the potency on *E. coli* (Table 2). This proves that we were successful in increasing NBTI potency, at least for the Gram-positive bacteria.

Substitution of phenyl with *p*-hydroxymethyl (**16**) and amide group (**17**) led to a significant decrease in activity, with the complete loss of *E. coli* gyrase and *S. aureus* Topo IV on-target activity as well as loss of Gram-negative potency. According to the results for target inhibition and whole cell activity, no hydrogen-bonding with gyrase or Topo IV was established. Regarding the compounds which are not able to form hydrogen or halogen bonds, it was interesting to note that compound **18** comprised of *p*-dimethylaminophenyl RHS was identified as highly active against *S. aureus* gyrase (IC_50_ = 0.067 μM), with moderate activity on *E. coli* gyrase, while no Topo IV activity was determined. According to our docking calculations, each of Ala68 is forming a pseudohydrogen bond with one methyl group, which might be the reason for high *S. aureus* gyrase activity [13]. Introduction of *di*-substituted phenyl RHS as in **19** and **20** demonstrated modest and balanced antibacterial activity on *S. aureus* gyrase and *E. coli* Topo IV, which was slightly better for **20** containing free hydroxyl group. Particularly interesting was its antibacterial activity against *C. jejuni*, which was comparable to the most efficient compound **15**, although its antibacterial potency on all other Gram-negative strains was rather moderate. Compounds **21** and **22** comprising substituted pyridine showed gyrase inhibitory activity on both bacterial strains comparable to **19** and **20**, but unfortunately lost Topo IV inhibitory activity.

**Figure 5:**
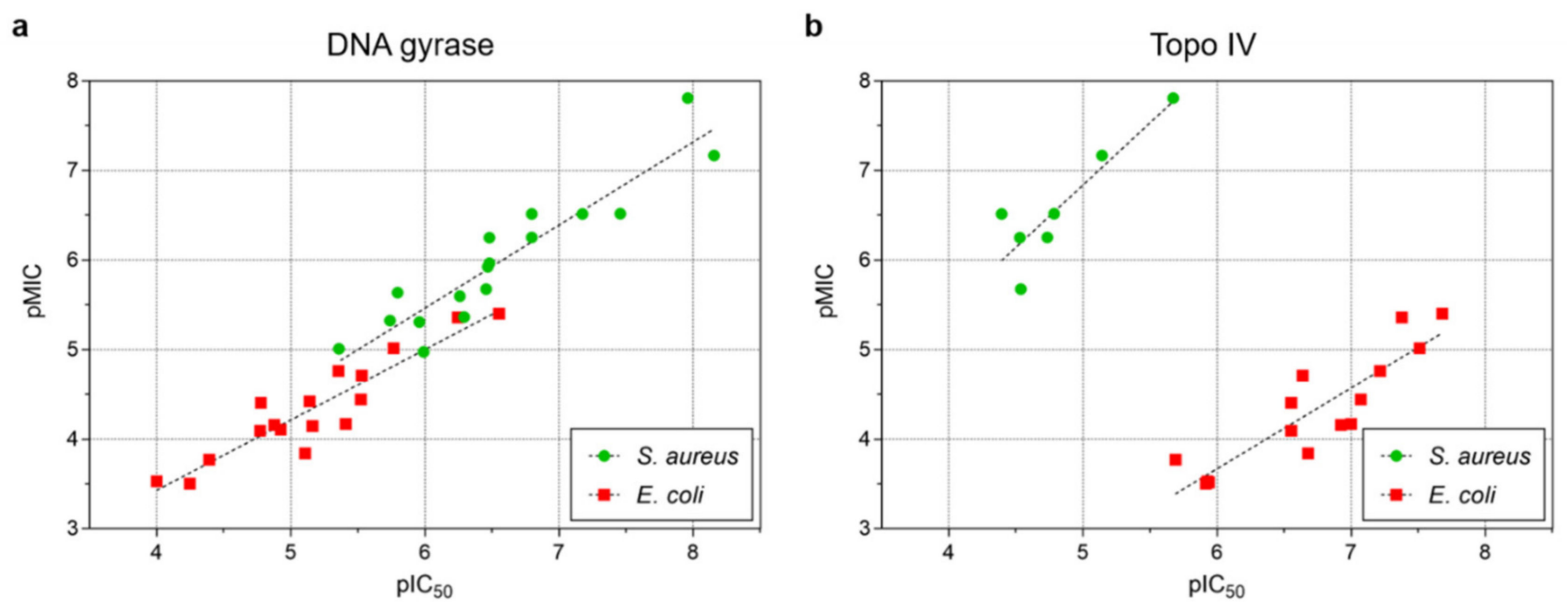
The correlation between antibacterial potency (pMIC = −logMIC in molar) of NBTIs and the corresponding *S. aureus* and *E. coli* inhibitory activity (pIC_50_ = −logIC_50_ in molar) of: (**a**) DNA gyrase and (**b**) Topo IV, respectively. A linear correlation may be observed for DNA gyrase of both bacterial strains (*R*^2^ = 0.864 and 0.856 for *S. aureus* and *E. coli*, respectively), while slightly inferior linear correlation can be obtained for Topo IV (*R*^2^ = 0.788 and 0.790 for *E. coli* and *S. aureus*).

A relatively high correlation was observed between the gyrase pIC_50_ values and corresponding pMIC of R^2^ = 0.864 and 0.856 for *S. aureus* and *E. coli*, respectively (Figure 5), indicating that DNA gyrase is the pivotal target responsible for the observed whole cell antibacterial activity in *S. aureus* strains. The correlation for Topo IV was slightly lower, with R^2^ = 0.788 and 0.790 for *E. coli* and *S. aureus*, respectively. Although, the compounds expressed potent enzyme inhibitory activity against *E. coli* Topo IV, their whole cell potency on *E. coli* (expressed as pMIC) is somehow less proportional to Topo IV pIC_50_ (Figure 5). The same ratio is observed for *S. aureus* pair of enzymes. This is a direct consequence of DNA gyrase being the pivotal bacterial target for *S. aureus* and as such its inhibition governs the antibacterial activity. The comparison on *E. coli* pair of enzymes is not that clear; even though Topo IV is the primary target in isolated enzyme assay, lower R^2^ is observed. This might be a consequence of the intrinsic difference in DNA gyrase and Topo IV importance in bacterial cell survival, or even an outcome of the lower compounds’ accumulation in *E. coli* due to efflux pumps, which does not follow the same SAR as the one for Topo IV. The observed selectivity of NBTIs for two related targets might be explained by the differences in amino acid residues constituting NBTIs’ binding pocket of DNA gyrase and Topo IV for both strains (Supplementary Materials Figure S3). The main difference is the amino acid residue Met75 in *S. aureus* DNA gyrase that corresponds to Ile74 in *E. coli* DNA gyrase, Ile71 in *S. aureus* Topo IV and Leu71 in *E. coli* Topo IV, respectively. Ile residues (*S. aureus* Topo IV, *E. coli* DNA gyrase) are expected to pose higher steric hindrance thus preventing the binding of even smaller NBTI RHSs, while Met75 (*S. aureus* DNA gyrase) and Leu71 (*E. coli* Topo IV) residues allow just the right space to accommodate monocyclic RHSs [10].

**Figure 6:**
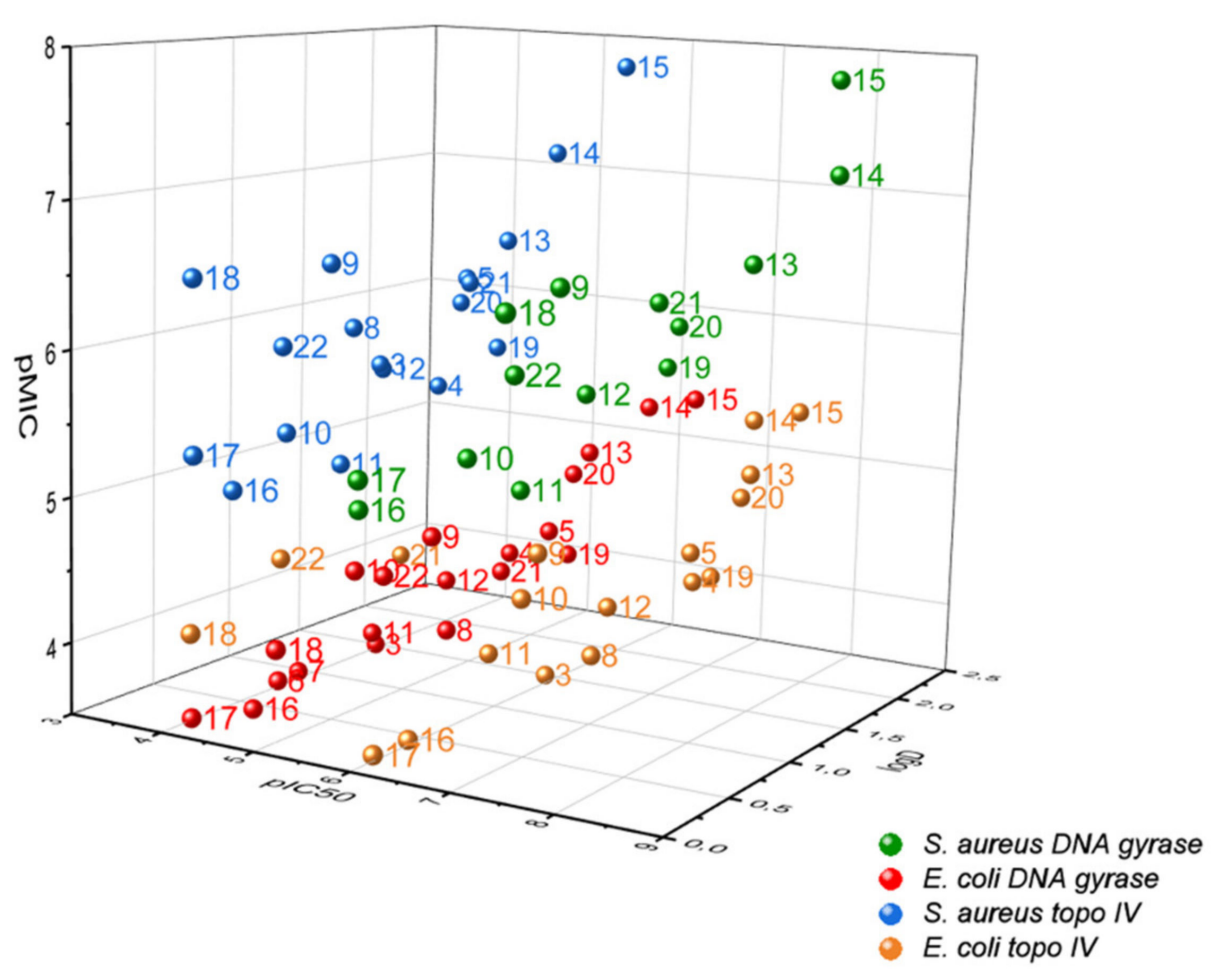
3D plot representing influence of the calculated logD (pH = 7.4) on pIC_50_ and pMIC against all antibacterial targets. The graph clearly indicates that increasing lipophilicity increases enzymatic activity (IC_50_) and antibacterial potency (pMIC).

Since the compounds exhibit potent enzyme inhibitory activity in general, their whole cell potency depends particularly on the uptake into Gram-positive and Gram-negative bacteria (apart from being substrates for the previously mentioned efflux pumps), which depends on their physicochemical properties. In this context, one should be aware about that cell envelopes of Gram-positive and Gram-negative pathogens differ substantially in composition. This is particularly important in the case of Gram-negatives whose cell envelope, in particular its outer layer (that is missing in Gram-positives) is extremely protective for passage of highly lipophilic molecular entities due to tight packing of its lipopolysaccharide (LPS) rich content [27]. Additionally, the aquaporins are an integral part of the outer layer and restrict the diffusion of hydrophilic molecules with molecular weight (MW) around 700 Da [28]. Put differently, more positively charged (i.e., increased basicity) and suitably polar (i.e., lower logD) compounds with appropriate size (MW ~ 400–500, as for the majority of NBTIs) tend to have better permeation in Gram-negative bacterial pathogens [29]. Taking this into consideration, we further examined the relationship between the compound’s lipophilicity and the enzyme inhibitory activity and the whole cell potency by plotting the data on a 3D graph (Figure 6). The 3D plot distinguishes separated clusters for each of the bacterial targets (*S. aureus* gyrase (green), Topo IV (blue) and *E. coli* gyrase (red), Topo IV (yellow)) divided by the correlation between pIC_50_ and logD as independent variables, and pMIC as a dependent variable. On one hand, the increase in MICs is a reasonable consequence of the increase in the halogen size (**12**–**15**) and aforementioned rise in halogen interactions potency. On other hand, the increase in lipophilicity (logD) potentiates antibacterial activity (MIC), which is particularly noticeable for compounds **13**–**15** and **20** having highest logD values (~1.9–2.2), compared to compounds **16** and **17** with the lowest logD (0.44 and 0.19, respectively). These findings are congruent with the previously determined, specific, low to moderate lipophilicity range (logD ~ 0–3) that is optimal for passive cytoplasmic permeation, particularly in Gram-positive bacteria [30,31]. Nevertheless, the data represented in Figure 6 point to an important conclusion, i.e., a deft balance in NBTIs lipophilicity and on-target potency has to be achieved for high antibacterial potency. On one hand, the secondary amine and overall hydrophilicity has to be kept in order to achieve crucial interactions with DNA gyrase and Topo IV as well as to increase the overall optimal ligands’ solubility (that is decisive for passing the aquaporins rich outer layer of cell envelope in Gram-negative bacteria). On the other hand, higher, but not excessive lipophilicity increases cellular permeability (Supplementary Materials Table S1) and thus antibacterial activity especially on Gram-positive bacteria. Indeed, on-target activity is a prerequisite for antibacterial activity; however, how lipophilicity/hydrophilicity influence the antibacterial activity could be best observed by comparing DNA gyrase/Topo IV inhibition and antibacterial activity of compounds **18**–**20**. Although, **19** is distinguished by the most equilibrated inhibition of DNA gyrase/Topo IV, both **18** and **20** surpass the latter in antibacterial activity. For **18** this might be a consequence of more potent inhibition of *S. aureus* DNA gyrase. **20** has in general more potent antibacterial activity on both Gram-positive and Gram-negative bacteria albeit having very comparable on-target potency as **19**. The reason for such a difference in activity can be attributed to the fine-tuned lipophilic/hydrophilic properties of **20**; these allow sufficient compound solubility and cell permeability; however, due to slightly increased hydrophilicity, **20** is probably a better substrate for Gram-negative aquaporins (for passing the highly restrictive outer layer), i.e., optimally lipophilic (for passing not only LPS compartment of the outer layer, but also the inner, lipid rich cytoplasmic layer) [31,32] and/or a worse substrate for the efflux pumps (see below). Further evidence on the influence of the lipophilicity/hydrophilicity balance on the antibacterial activity may be seen by comparing the most potent compound from our previous study [18] and compound **5** (both depicted in Figure 2). Approximately 4-fold increase in potency on *E. coli* DNA gyrase resulted in the same increase in antibacterial potency (evaluated by comparing MIC values). However, although we observed approximately the same increase in the potency on isolated enzyme from *S. aureus*, the antibacterial potency increased even more (8-fold). This increase in antimicrobial potency is apparently not influenced solely by the on-target activity. Since **5** is more hydrophilic than the amidocyclohexane derivative, one would expect it to have poorer permeability properties. Instead, the increase in antibacterial potency on *S. aureus* of **5** can only be explained by better permeability properties or the non-susceptibility to the efflux pumps. Increase in permeability is unlikely since the starting compound is slightly more lipophilic, but it is likely that lower lipophilicity of **5** makes it poorer substrate for *S. aureus* efflux pumps [33,34]. A similar conclusion can also be reached when comparing compounds activity on *E. coli* wild-type, and *E. coli* D22 and N43 strains. The increased membrane permeability of *E. coli* D22 increases the antibacterial potency up to 16× (2× in majority of cases), where the latter is improved the same or even more in *E. coli* N43 strain with removed efflux pumps (32× in majority of cases). This led us to a conclusion that the compounds have some difficulties in passing into the *E. coli* and are allegedly pumped-out by the efflux pumps. As a consequence, the accumulation of the compounds in *E. coli* is significantly reduced, and it is most likely the pivotal reason for their lower whole cell activity. This is in line with the previous observation that the efflux pump containing AcrA protein is known to bind preferably lipophilic compounds [28,35] and shows that delicate balance, i.e., a “sweet spot” in compounds lipophilicity has to be achieved in order to assure both permeability and the absence of efflux pumps susceptibility. This “sweet spot” needs to be defined for each organism, and in *S. aureus* seems to be close to logD = 2.

### 2.4. Cytotoxicity Studies

In vitro safety profile was determined by cytotoxicity assessment on human umbilical vein endothelial (HUVEC) and HepG2 liver cancer cell lines, and by the blockage of hERG potassium ion channel. Cytotoxicity on human HUVEC and HepG2 cell lines was assessed by compounds’ effect on cell metabolic activity (Table 4). Slightly higher toxicity was observed on HepG2 cell line than on HUVEC. In general, cytotoxic IC_50_ values against both cell lines were higher in comparison to corresponding *S. aureus* MIC values. The most potent compounds **13**, **14**, **15**, and **18** showed up to 3 orders of magnitude difference between *S. aureus* MIC and IC_50_s of both cell lines, with highest cytotoxicity on HepG2 cells being approximately 10 μM, while MIC down to 0.016 μM was measured for compound **15** (Supplementary Materials Table S4). The majority of compounds exhibited higher *E. coli* whole cell potency than cytotoxic IC_50_ values, especially in comparison to HepG2 cell line. Comparing the data for *E. coli* N43 strain with removed efflux pumps, two orders of magnitude higher potency was evident for compounds **14** and **15** on bacterial strains compared to human HUVEC and HepG2 cell lines (0.16 μM for *E. coli* N43 vs. ~20 μM for HUVEC and ~10 μM for HepG2 of compound **15**; Supplementary Materials Table S4). These results lead us to a reasonable conclusion that potency on bacterial strains does not overlap with cytotoxicity and hence more potent compounds on bacteria display better safety profile. For compounds **3**, **6**, **7**, **16**, and **17** that were poorly or non-active, only metabolic cell activity and not cytotoxic IC_50_ was determined (Supplementary Materials Table S5). None of the compounds inhibited human topoisomerase IIα enzyme at concentration 10 μM; however, the majority of them appeared active on HUVEC and HepG2 cells, from which we can conclude that other mechanisms likely participate to the overall cell toxicity.

As has already been recognized for NBTIs reported in the literature, this series of antibacterials suffers from undesired potassium channel inhibition or the hERG cardiac toxicity (Table 4). The hERG blockage induced by compounds **15** and **20** with IC_50_ of 0.18 μM is of particular concern, being only one order of magnitude above the target potency (MIC = 0.016 μM; Supplementary Materials Table S4) of the most favorable compound **15**. A general trend between the gyrase enzyme potency and cardiotoxicity accompanying all NBTIs could be observed here as well; higher potency coincides with stronger hERG inhibition [15]. We should note that **14** and **15**, albeit provoking significant hERG inhibition, have the ratio between hERG (IC_50_ = 0.18 μM for **15**) and IC_50_/MIC (IC_50_ = 0.011 μM and MIC = 0.016 μM for **15**) spanning from 1-2 order of magnitude. This reveals that acceptable therapeutic window might be achieved; however, further optimizations towards lower hERG inhibition and better safety profile is still required.

## 3. Conclusions

In summary, herein we describe a rational design, synthesis, and biological characterization of a series of NBTIs active against DNA gyrase and Topo IV, based on structurally new, mainly monocyclic innovative RHS moieties. In general, these derivatives display potent broad-spectrum antibacterial activity against a panel of clinically relevant Gram-positive and Gram-negative bacterial pathogens, with significant effect also on *Mycobacterium* species. In the course of our research, we have identified a subseries of highly active compounds featuring *p*-halogenated phenyl RHSs with extremely potent whole cell activity on *S. aureus*, due to symmetrical bifurcated halogen-bonding with the backbone carbonyl oxygen of Ala68 from both GyrA subunits, i.e., the first halogen bond of this type identified between a ligand and its target in a biological system. Bromo (**14**) and iodo (**15**) derivatives displayed remarkable inhibitory activities against DNA gyrase from *S. aureus* (IC_50_ of 0.007 μM for **14** and 0.011 μM for **15**, respectively) and *E. coli* Topo IV (IC_50_ of 0.042 μM for **14** and 0.021 μM for **15**, respectively), which are among the most potent NBTIs reported to date. Furthermore, compound **15** showed excellent potency against Gram-positive strains (MIC = 0.0078 μg/mL and 0.015 μg/mL for *S. aureus* and *S. pneumoniae*, respectively) and it was the most active compound against Gram-negative strains, including *E. coli* (MIC ~ 0.25–2.0 μg/mL), *A. baumannii* (MIC = 0.25 μg/mL), *C. jejuni* (MIC = 0.015 μg/mL), and *P. aeruginosa* (MIC = 32 μg/mL) belonging to the list of most critical pathogens, as reported by the WHO and CDC. Moreover, it appears that DNA gyrase is the pivotal antibacterial target for NBTIs activity in Gram-positive bacteria comparing to Topo IV, as demonstrated by its higher pMIC-pIC_50_ correlation. In conjunction with this findings, it seems that NBTIs enzyme activity and whole cell potency are directly dependent on a specific, fine-tuned lipophilicity/hydrophilicity ratio for these compounds that governs their permeation through the bacterial membranes, in particular the highly restrictive outer barrier of Gram-negative bacteria. Indeed, lipophilicity itself (logD ~ 0–2.5) is apparently optimal for passing of NBTIs through the bi-lipid compartment of Gram-positive bacterial membranes, but once reached seems not to be a decisive factor. However, the lower, not excessive lipophilicity as well as the ligand’s size are apparently decisive physicochemical/molecular determinants for passing of these compounds through Gram-negative bacterial membranes (in particular the outer layer’s LPS barrier and aquaporins) and reaching their intracellular targets. We believe and strive to a further optimization toward achieving much wider antibacterial spectrum and a more balanced Gram-positive and Gram-negative potency. Due to relatively potent hERG inhibition, our future efforts would be majorly invested in further improvements of the antibacterial activities as well as reduction of hERG cardiotoxicity.

## 4. Materials and Methods

### 4.1. Biopharmaceutical Classification

Prior to the measurements of solubility and permeability isocratic HPLC methods were developed. An Agilent 1100 system (degasser, bin. pump, well plate sampler, column thermostat and diode array detector) was used with a Waters Xterra MS C18, 3.5 µm, 4.6 × 100 mm column and a mobile phase from A (0.5% ammonium phosphate buffer at pH = 3.0) and B (acetonitrile). The individual method parameters for each compound are given in the Supplementary Materials Table S1.

#### 4.1.1. Solubility

The equilibrium solubility was determined by first weighing approximately 2 mg of the tested or reference compound to a 1.5 mL microcentrifuge vial and then adding 0.5 mL of 0.1 mm glass beads and 200 µL of pH 6.8 phosphate buffered saline (PBS—1.0 g potassium dihydrophosphate, 2.0 g dipotassium hydrophosphate and 8.5 g of sodium chloride dissolved in 1.0 L of purified water). The vials were then placed in a homogenizer (Next Advance Bullet Blender, Troy, NY, USA) for 5 min ad speed setting “2” after which they were kept in a thermostated shaker (Tehtnica HeatMix, Domel, Slovenia) at 37 °C and 1200 RPM for 24 h. The supernatants were obtained after a 10 min centrifugation at 15,000× *g* (Eppendorf Centrifuge 5415R, Eppendorf AG, Hamburg, Germany) and were immediately stabilized by a 12-fold dilution with the same buffer as used for the dissolution process. Twenty µL of these solutions were injected for the HPLC analysis by the described methods (Supplementary Materials Table S1). External standards for calibration were made in a mobile phase with 5% acetonitrile in a range of 30–90 mg/L and injected at 20 µL injection volumes. Less than 15% of the solid was dissolved during the incubation for most compounds except for compounds **9**, **10**, and **16** with 63%, 30%, and 20% of the solid consumed, respectively. The method accuracy was confirmed by the coefficient of variation which was 5.0%, 1.2%, 1.1%, and 2.0% for the reference compounds metoprolol, verapamil, theophylline, and digoxin, respectively.

#### 4.1.2. Permeability

The donor solutions for PAMPA permeability measurements were obtained by dissolving the remaining solids from the solubility measurements in 750 µL of pH 7.4 PBS (2.38 g disodium hydrophosphate, 0.19 g potassium dihydrophosphate, and 8.0 g of sodium chloride) with 20% of methanol in the same homogenizer and the same settings as described above and then diluting the supernatants obtained under the same centrifugation conditions two-fold with pH 7.4 PBS to yield 10% methanol as a donor solution medium. A 96-well precoated three-layer PAMPA plates (Corning^®^ Gentest™ Pre-coated PAMPA Plate System, Corning, NY, USA) were used according to the manufacturer’s instructions and as described previously [36] with 300 µL of the donor solutions in the bottom compartment and 200 µL of the acceptor solution (pH 7.4 PBS) in the upper compartment at 37 °C and a 4-h incubation time. The initial and end donor solutions were injected at a 5 µL injection volume. The initial donor solutions were also diluted 100-fold and injected at a 100 µL injection volume to serve as external references for the end acceptor solutions which were injected at the same injection volume. The permeability calculations were made with a manufacturer-provided Excel spreadsheet directly from the measured peak areas accounting for the relevant dilutions and injection volumes. The concomitantly measured standards for permeability classification metoprolol (high permeability), verapamil (high), theophyllin (high), digoxin (intermediate), atenolol (low), and furosemide (low) were prepared at 200 µM concentrations for the donor solutions in a PBS with 10% methanol and thereafter treated the same as the tested compounds. Measurements were made in quadruplicates with the coefficient of variation up to 26% for the highly permeable compounds. The “high” permeability of the tested compounds was designated when it was higher than that of theophylline (lowest permeability of a “high” permeability reference drug and “intermediate” permeability was designated when it was above the value for digoxin and below teophyllin.

### 4.2. Homology Modeling

The structural homology model for the enzyme Topo IV was built using the protein structure homology modelling server Swiss model. First, the target sequence for both subunits was downloaded from UniProt repository in FASTA format (protein ID for *E. coli* parC: P0AFI2, parE: P20083 and for *S. aureus* parC: Q2FYS4, parE: Q2FYS5). Then the Swiss model template library was searched for all possible templates. Suitable templates were selected (PDB ID: 3KSA for *E. coli* and PDB ID: 3RAF for *S. aureus*), considering the following values: coverage, GMQE score, identity and resolution (Supplementary Materials Table S3). After building the model the correspondence between the homologous model and the template was visually checked.

### 4.3. Molecular Docking Calculations

Molecular docking calculations of novel NBTI compounds within the NBTI binding pocket of *S. aureus* and *E. coli* DNA gyrase and *S. aureus* and *E. coli* topo IV were performed by GOLD docking suite in a flexible fashion [37]. Crystal structure of *S. aureus* DNA gyrase in complex with DNA and an intercalated NBTI ligand (GSK299423; PDB ID: 2XCS) and our constructed *E. coli* DNA gyrase [18], *S. aureus* topo IV and *E. coli* topo IV homology models were used for calculations. The experimental coordinates of the co-crystallized NBTI ligand (GSK299423) were used to define the binding site (cavity radius of 16 Å for *S. aureus* DNA gyrase, 17 Å for *E. coli* DNA gyrase and 15.5 Å for *S. aureus* and *E. coli* topo IV, respectively) and all ligands were protonated. The same settings and technical parameters of the GOLD genetic algorithm (population size = 100, selection pressure = 1.1, number of operations = 100.000, number of islands = 5, niche size = 2, migrate = 10, mutate = 95, cross-over = 95) were used for all calculations by docking each molecule 10 times into the binding site. Amino acids Met75/Leu74/Ile71/Leu74 on α3 helix, Asp83/Asp82/Asp79/Asp79 on α4 helix and Met121/Met120/Met117/Met118 were treated as flexible during docking. The molecular docking protocol was validated by re-docking the co-crystallized NBTI ligands (GSK299423) 3-fold to reproduce their spatial conformation and orientation. The heavy-atoms root-mean-square deviation (RMSD ≤ 2.0 Å) between each calculated docking pose and co-crystallized ligand conformation served as decisive criteria for quality of all structure-based settings [38]. The quality of the calculated docked poses was first visually examined in terms of their correct spatial orientation and conformation within the NBTI binding pocket relative to the natively present co-crystallized ligand conformation and additionally by the calculated GOLDScore Fitness function [37].

### 4.4. Analytical Techniques

All chemicals were obtained from commercial sources and were used without further purification. The reactions requiring anhydrous conditions were carried out under inert argon atmosphere with anhydrous solvents. Reactions were monitored by thin-layer chromatography (TLC) on Merck silica gel (60 F254) plates (0.25 mm thick), visualized by UV light and/or staining reagents. Column chromatography for compound purification of the final compounds was carried out on silica gel 60 (particle size 0.040–0.063 mm; Merck, Kenilworth, NJ, USA). HPLC purity was determined on Thermo Scientific DIONEX UltiMate 3000 instrument equipped with diode array detector using Acquity UPLC^®^ BEH C8 column (1.7 μm. 2.1 mm × 50 mm). The solvent system consisted of 0.1% trifluoroacetic acid in water (A) and acetonitrile (B), employing the following gradient: 90% A to 50% A in 6 min, then 50% A for 3 min, with flow rate 0.3 mL/min and injection volume 5 μL. Melting points were determined on a Reichert hot stage microscope and are uncorrected. NMR spectra (^1^H and ^13^C) were obtained on a Bruker AVANCE III spectrometer (Bruker Corporation. Billerica, MA, USA) at 400 and 100 MHz, respectively in DMSO-d6 or CDCl_3_ solution with tetramethylsilane (TMS) as an internal standard. High resolution mass spectra were obtained on Advion CMS (Advion Inc., Ithaca, NY, USA) or VG Analytical Autospec Q mass spectrometer (Fisons, VG Analytical, Manchester, UK). IR spectra were recorded on Thermo Nicolet Nexus 470 ESP FT-IR spectrometer (Thermo Fisher Scientific, Waltham, MA, USA).

### 4.5. Synthesis

Synthesis and analytical characterization of compounds **11**–**18** is described elsewhere [13].

### 4.5.1. 1-(2-(6-Methoxy-1,5-naphthyridin-4-yl)ethyl)piperidin-4-amine (**2**)

Tert-butyl 1-(2-(6-methoxy-1,5-naphthyridin-4-yl)ethyl)piperidin-4-ylcarbamate (0.63 g, 1.63 mmol, 1 equiv) was dissolved in 12.5 mL of 1 M HCl in acetic acid and stirred for 30 min at room temperature. Solvent was evaporated, the residue dissolved in 30 mL of saturated NaHCO_3_(aq) and pH adjusted to 12 with 1 M NaOH. Water layer was washed with DCM (3 × 30 mL), combined organic layers dried over Na_2_SO_4_ and concentrated in vacuum to afford compound **2** as dark-brown viscous liquid (0.33 g, 70.7%). Rf = 0.10 (DCM/MeOH 4:1 + 1% Et_3_N). ^1^H NMR (400 MHz, CDCl_3_) δ = 1.41–1.51 (m, 2H; N(CH_2_CH_2_)_2_CH), 1.88 (d, *J* = 11.9 Hz, 2H; N(CH_2_CH_2_)_2_CH), 2.20 (t, *J* = 11.0 Hz, 2H; N(CH_2_CH_2_)_2_CH), 2.66–2.78 (m, 1H; N(CH_2_CH_2_)_2_CH), 2.73–2.84 (m, 2H; CH_2_CH_2_N), 3.05 (d, *J* = 11.5 Hz, 2H; N(CH_2_CH_2_)_2_CH), 3.30–3.46 (m, 2H; CH_2_CH_2_N), 4.08 (s, 3H; CH_3_O), 7.11 (d, *J* = 9.0 Hz, 1H; Ar–H), 7.41 (d, *J* = 4.5 Hz, 1H; Ar–H), 8.19 (d, *J* = 9.0 Hz, 1H; Ar–H), 8.66 (d, *J* = 4.5 Hz, 1H; Ar–H) ppm. ^13^C NMR (100 MHz, CDCl_3_): δ = 28.46, 35.83, 52.37, 53.72, 58.37, 77.23, 116.31, 124.24, 140.33, 140.97, 141.48, 146.64, 147.70, 161.43 ppm. IR (ATR): ν = 3271, 2941, 2811, 1614, 1590, 1503, 1489, 1438, 1398, 1334, 1125, 1079, 1018, 932, 853, 807, 751, 713, 615, 584, 536 cm^–1^. HRMS (ESI): *m*/*z* calculated for C_16_H_22_N_4_O_14_ + H^+^: 287.1872 [M + H]^+^, found: 287.1875.

#### 4.5.2. General Procedure for Reductive Amination

To a solution of amine 2 (1 equiv) in dry methanol, the appropriate aldehyde (1–1.3 equiv) was added together with the catalytic amount of acetic acid. The reaction mixture was stirred for 2 h at the room temperature under the inert atmosphere. Next, the reaction mixture was cooled to 0 °C and NaCNBH_3_ (3–5 equiv) dissolved in small amount of dry methanol was added drop-wise. After stirring overnight at room temperature under inert atmosphere, the solvent was evaporated in vacuum and the residue diluted in ethyl acetate. Organic phase was washed with saturated Na_2_CO_3_ three times, dried over Na_2_SO_4_, filtered and the solvent evaporated under reduced pressure. The residue was purified by flash column chromatography to afford appropriate substituted product.

#### 4.5.3. 1-(2-(6-Methoxy-1,5-naphthyridin-4-yl)ethyl)-N-((1-methyl-1H-benzo[d]imidazol-2-yl)methyl)piperidin-4-amine (**3**)

According to the general procedure for reductive amination, compound **2** (0.15 g, 0.524 mmol, 1 equiv) was used together with the 1-methyl-1*H*-benzo[d]imidazole-2-carbaldehyde (0.101 g, 0.631 mmol, 1.2 equiv). The residue was purified by flash column chromatography using CHCl_3_/MeOH (15:1 following 9:1 + 1% Et_3_N) to afford compound **3** (0.083 g, 36.6%) as a brown-red solid. Rf = 0.17 (CHCl_3_/MeOH 15:1 + 1% Et_3_N). m.p. 57–62 °C. ^1^H NMR (400 MHz, CDCl_3_): δ = 1.43–1.61 (m, 2H; N(CH_2_CH_2_)_2_CH), 1.99 (d, *J* = 11.2 Hz, 2H; N(CH_2_CH_2_)_2_CH), 2.21 (t, *J* = 10.8 Hz, 2H; N(CH_2_CH_2_)_2_CH), 2.59–2.69 (m, 1H; N(CH_2_CH_2_)_2_CH), 2.78–2.82 (m, 2H; CH_2_CH_2_N), 3.06 (d, *J* = 11.6 Hz, 2H; N(CH_2_CH_2_)_2_CH), 3.33–3.42 (m, 2H; CH_2_CH_2_N), 3.84 (s, 3H; CH_3_N), 4.08 (s, 3H; CH_3_O), 4.12 (s, 2H; NHCH_2_), 7.11 (d, *J* = 9.0 Hz, 1H; Ar–H), 7.23–7.31 (m, 2H; 2 × Ar–H) *, 7.32–7.36 (m, 1H; Ar–H), 7.41 (d, *J* = 4.5 Hz, 1H; Ar–H), 7.70–7.75 (m, 1H; Ar–H), 8.18 (d, *J* = 9.0 Hz, 1H; Ar–H), 8.66 (d, *J* = 4.5 Hz, 1H; Ar–H) ppm. ^13^C NMR (100 MHz, CDCl3): δ = 28.43, 29.96, 32.52, 43.89, 52.18, 53.74, 58.39, 77.24*, 109.09, 116.32, 119.46, 121.94, 122.49, 124.25, 136.16, 140.33, 140.96, 141.48, 142.26, 146.57, 147.70, 153.18, 161.43 ppm. IR (ATR): ν = 3390, 2932, 2798, 1611, 1590, 1501, 1490, 1480, 1447, 1400, 1353, 1333, 1261, 1201, 1187, 1111, 1077, 1012, 874, 843, 770, 642, 584 cm^–1^. HRMS (ESI): *m*/*z* calculated for C_25_H_30_N_6_O+H^+^: 431.2559 [M + H]^+^, found: 431.2563. HPLC purity (254 nm): 94.9%. * signal is overlapping with residual solvent peak.

#### 4.5.4. *N*-((1-(4-Fluorophenyl)-1*H*-pyrazol-4-yl)methyl)-1-(2-(6-methoxy-1,5-naphthyridin-4-yl)ethyl)piperidin-4-amine (**4**)

According to the general procedure for reductive amination, compound **2** (0.220 g, 0.768 mmol, 1 equiv) was used together with the 1-(4-fluorophenyl)-1*H*-pyrazole-4-carbaldehyde (0.220 g, 1.157 mmol, 1.5 equiv). The residue was purified by flash column chromatography using CHCl_3_/MeOH (15:1 + 1% Et_3_N) to afford compound **4** (0.220 g, 41.3%) as a light brown solid. Rf = 0.12 (DCM/MeOH 4:1 + 1% Et_3_N). m.p. 59–74 °C. ^1^H NMR (400 MHz, CDCl_3_): δ = 1.45–1.55 (m, 2H; N(CH_2_CH_2_)_2_CH), 1.98 (d, *J* = 12.0 Hz, 2H; N(CH_2_CH_2_)_2_CH), 2.19 (t, *J* = 10.9 Hz, 2H; N(CH_2_CH_2_)_2_CH), 2.53–2.70 (m, 1H; N(CH_2_CH_2_)_2_CH), 2.75–2.85 (m, 2H; CH_2_CH_2_N), 3.07 (d, *J* = 11.7 Hz, 2H; N(CH_2_CH_2_)_2_CH), 3.37–3.41 (m, 2H; CH_2_CH_2_N), 3.81 (s, 2H; NHCH_2_), 4.08 (s, 3H; CH_3_O), 7.09–7.17 (m, 3H; 3 × Ar–H), 7.42 (d, *J* = 4.5 Hz, 1H; Ar–H), 7.60–7.65 (m, 2H; 2 × Ar–H), 7.65 (s, 1H; Ar–H), 7.84 (s, 1H; Ar–H), 8.18 (d, *J* = 9.0 Hz, 1H; Ar–H), 8.66 (d, *J* = 4.5 Hz, 1H; Ar–H) ppm. ^13^C NMR (100 MHz, CDCl_3_): δ = 28.46, 32.66, 40.73, 52.35, 53.72, 58.41, 77.24 *, 116.22 (d, *J* = 23.0 Hz), 116.29, 120.66 (d, *J* = 8.3 Hz), 124.25, 125.67, 136.57, 140.36, 140.76, 140.98, 141.50, 146.69, 147.72, 161.02 (d, *J* = 245.8 Hz), 161.42, 163.59 ppm. IR (ATR): ν = 2927, 2803, 1613, 1591, 1489, 1458, 1443, 1400, 1334, 11263, 1216, 1157, 1126, 1098, 1077, 1042, 1019, 952, 851, 836, 810, 774, 768, 743, 713, 661, 625, 524 cm^–1^. HRMS (ESI): *m*/*z* calculated for C_26_H_29_FN_6_O + H^+^: 461.2465 [M + H]^+^, found: 461.2452. HPLC purity (254 nm): 90.9%. * signal is overlapping with residual solvent peak.

#### 4.5.5. 1-(2-(6-Methoxy-1,5-naphthyridin-4-yl)ethyl)-*N*-((1-phenyl-1*H*-pyrazol-4-yl)methyl)piperidin-4-amine (**5**)

According to the general procedure for reductive amination, compound **2** (0.220 g, 0.768 mmol, 1 equiv) was used together with the 1-phenyl-1*H*-pyrazole-4-carbaldehyde (0.198 g, 1.150 mmol, 1.5 equiv). The residue was purified by flash column chromatography using CHCl_3_/MeOH (15:1) to afford compound **5** (0.220 g, 43.23%) as an orange-red viscous liquid. Rf = 0.19 (DCM/MeOH 4:1 + 1% Et_3_N). ^1^H NMR (400 MHz, CDCl_3_): δ = 1.48–1.59 (m, 2H; N(CH_2_CH_2_)_2_CH), 2.00 (d, *J* = 14.3 Hz, 2H; N(CH_2_CH_2_)_2_CH), 2.19 (t, *J* = 10.8 Hz, 2H; N(CH_2_CH_2_)_2_CH), 2.60–2.70 (m, 1H; N(CH_2_CH_2_)_2_CH), 2.77–2.83 (m, 2H; CH_2_CH_2_N), 3.09 (d, *J* = 11.9 Hz, 2H; N(CH_2_CH_2_)_2_CH), 3.36–3.42 (m, 2H; CH_2_CH_2_N), 3.82 (s, 2H; NHCH_2_), 4.08 (s, 3H; CH_3_O), 7.11 (d, *J* = 9.0 Hz, 1H; Ar–H), 7.24–7.30 (m, 1H; Ar–H) *, 7.40–7.45 (m, 3H; 3 × Ar–H), 7.65–7.69 (m, 3H; 3 × Ar–H), 7.93 (s, 1H, Ar–H), 8.19 (d, *J* = 9.0 Hz, 1H; Ar–H), 8.66 (d, *J* = 4.5 Hz, 1H; Ar–H) ppm. ^13^C NMR (100 MHz, CDCl_3_): δ = 28.43, 32.58, 40.69, 52.33, 53.70, 59.19, 58.37, 116.29, 118.88, 119.27, 122.46, 124.23, 125.59, 126.31, 129.42, 140.12, 140.27, 140.73, 140.96, 141.43, 146.72, 147.63, 161.41 ppm. IR (ATR): ν = 2941, 2807, 1612, 1594, 1502, 1489, 1463, 1437, 1398, 1373, 1333, 1259, 1105, 1076, 1017, 953, 851, 807, 712, 690, 653, 610, 596, 584, 508 cm^–1^. HRMS (ESI): *m*/*z* calculated for C_26_H_30_N_6_O + H^+^: 443.2559 [M + H]^+^, found: 443.2560. HPLC purity (254 nm): 97.1%. * signal is overlapping with residual solvent peak.

#### 4.5.6. *N*-((2,8-Dimethylimidazo[1,2-a]pyridin-3-yl)methyl)-1-(2-(6-methoxy-1,5-naphthyridin-4-yl)ethyl)piperidin-4-amine (**6**)

According to the general procedure for reductive amination, compound **2** (0.252 g, 0.880 mmol, 1 equiv) was used together with the 2,8-dimethylimidazo[1,2-a]pyridine-3-carbaldehyde (0.157 g, 0.901 mmol, 1 equiv). The residue was purified by flash column chromatography using CHCl_3_/MeOH (9:1) to afford compound **6** (0.252 g, 62.9%) as an orange-brown solid. The synthesis of 2,8-dimethylimidazo[1,2-a]pyridine-3-carbaldehyde was previously reported [18]. Rf = 0.23 (DCM/MeOH 4:1 + 1% Et_3_N). m.p. 50–61 °C. ^1^H NMR (400 MHz, CDCl_3_): δ = 1.46–1.55 (m, 2H; N(CH_2_CH_2_)_2_CH), 1.97 (d, *J* = 10.1 Hz, 2H; N(CH_2_CH_2_)_2_CH), 2.22 (t, *J* = 10.8 Hz, 2H; N(CH_2_CH_2_)_2_CH), 2.48 (s, 3H; CH_3_Ar) 2.50–2.58 (m, 1H; N(CH_2_CH_2_)_2_CH), 2.60 (s, 3H; CH_3_Ar), 2.79–2.87 (m, 2H; CH_2_CH_2_N), 3.07 (d, *J* = 11.8 Hz, 2H; N(CH_2_CH_2_)_2_CH), 3.35–3.43 (m, 2H; CH_2_CH_2_N), 4.07 (s, 3H; CH_3_O), 4.11 (s, 2H; NHCH_2_), 6.70 (t, *J* = 6.8 Hz, 1H; Ar–H), 6.92–7.00 (m, 1H; Ar–H), 7.12 (d, *J* = 9.0 Hz, 1H; Ar–H), 7.41 (d, *J* = 4.5 Hz, 1H; Ar–H), 8.15 (d, *J* = 6.7 Hz, 1H; Ar–H), 8.18 (d, *J* = 9.0 Hz, 1H; Ar–H), 8.66 (d, *J* = 4.5 Hz, 1H; Ar–H) ppm. ^13^C NMR (100 MHz, CDCl_3_): δ = 13.38, 17.10, 28.37, 32.51, 39.66, 52.25, 53.73, 58.32, 77.23 *, 111.59, 116.35, 118.45, 122.38, 123.06, 124.26, 126.17, 139.85, 140.37, 140.93, 141.51, 144.94, 146.33, 147.72, 161.46 ppm. IR (ATR): ν = 3258, 2925, 2813, 1613, 1590, 1489, 1437, 1398, 1373, 1354, 1333, 1259, 1102, 1020, 983, 853, 807, 767, 745, 641, 584, 568 cm^–1^. HRMS (ESI): *m*/*z* calculated for C_26_H_32_N_6_O + H^+^: 445.2716 [M + H]^+^, found: 445.2704. HPLC purity (254 nm): 98.4%. * signal is overlapping with residual solvent peak.

#### 4.5.7. 8-Cyano-N-(1-(2-(6-methoxy-1,5-naphthyridin-4-yl)ethyl)piperidin-4-yl)imidazo[1,2-a]pyridine-2-carboxamide (**7**)

Compound **2** (0.209 g, 0.730 mmol, 1 equiv), TBTU (0.234 g, 0.729 mmol, 1 equiv) and Et_3_N (0.31 mL, 2.223 mmol, 3 equiv) were dissolved in 5 mL of CH_3_CN and stirred at the room temperature under inert atmosphere for 30 min. 8-cyanoimidazo[1,2-a]pyridine-2-carboxylic acid (0.209 g, 0.730 mmol, 1 equiv) dissolved in 3 mL of CH_3_CN was added drop-wise and the reaction mixture stirred overnight. The precipitate was filtered with vacuum and dried under the reduced pressure. The residue was purified with flash column chromatography using CHCl_3_/MeOH (9:1 + 1% Et_3_N) to afford compound **7** (0.209 g, 62.7%) as a white solid. The synthesis of 8-cyanoimidazo[1,2-a]pyridine-2-carboxylic acid was previously reported [18]. Rf = 0.3 (CHCl_3_/MeOH 9:1 + 1% Et_3_N). m.p. 175–179 °C. ^1^H NMR (400 MHz, CDCl_3_): δ = 1.63–1.75 (m, 2H; N(CH_2_CH_2_)_2_CH), 2.09 (d, *J* = 12.5 Hz, 2H; N(CH_2_CH_2_)_2_CH), 2.35 (t, *J* = 11.3 Hz, 2H; N(CH_2_CH_2_)_2_CH), 2.80–2.86 (m, 2H; CH_2_CH_2_N), 3.09 (d, *J* = 11.3 Hz, 2H; N(CH_2_CH_2_)_2_CH), 3.42–3.36 (m, 2H; CH_2_CH_2_N), 3.99–4.07 (m, 1H; N(CH_2_CH_2_)_2_CH), 4.09 (s, 3H; CH_3_O), 7.12 (d, *J* = 9.0 Hz, 1H; Ar–H), 7.38 (dd, *J*_1_ = 9.5 Hz, *J*_2_ = 1.7 Hz, 1H; Ar–H) 7.42 (d, *J* = 4.5 Hz, 1H; Ar–H), 7.66–7.70 (m, 1H; Ar–H), 8.19 (d, *J* = 9.0 Hz, 1H; Ar–H), 8.25 (s, 1H; Ar–H), 8.63–8.64 (m, 1H; Ar–H), 8.68 (d, *J* = 4.5 Hz, 1H; Ar–H) ppm. ^13^C NMR (100 MHz, CDCl_3_): δ = 28.45, 32.29, 46.57, 52.27, 53.72, 58.38, 77.23 *, 100.08, 115.16, 115.86, 116.33, 119.28, 124.22, 125.68, 132.66, 140.35, 140.96, 141.50, 142.33, 143.54, 146.55, 147.69, 160.89, 161.44 ppm. IR (ATR): ν = 3403, 3276, 3128, 2948, 2814, 2365, 2229, 2176, 1661, 1650, 1612, 1591, 1564, 1503, 1430, 1400, 1335, 1302, 1258, 1230, 1137, 1121, 1074, 1018, 855 cm^–1^. HRMS (ESI): *m*/*z* calculated for C_25_H_25_N_7_O_2_ + H^+^: 456.2148 [M + H]^+^, found: 456.2143. HPLC purity (254 nm): 99.8%. * signal is overlapping with residual solvent peak.

#### 4.5.8. 2-Phenyl-2H-1,2,3-triazole-4-carbaldehyde (**23**)

The compound was synthesized according to the procedure from the literature (Supplementary Materials Scheme S1) [39]. To a suspension of glucose (5 g, 27.8 mmol, 1 equiv) in 50 mL of water, phenylhydrazine hydrochloride (12 g, 83.3 mmol, 3 equiv), sodium acetate (14 g, 166.8 mmol, 6 equiv) and catalytic amount of acetic acid were added. The reaction mixture was refluxed for 1h. After reaction was complete, the yellow residue was collected by vacuum filtration (7.308 g, 73.4%). Rf = 0.48 (DCM/MeOH 4:1 + 1% Et_3_N). ^1^H NMR (400 MHz, DMSO-d6): δ = 3.40–3.50 (m, 2H; 2 × CH(OH)), 3.53–3.65 (m, 2H; CH_2_(OH)), 4.34–4.37 (m, 1H; CH(OH)), 4.51–4.53 (m, 1H; CH_2_(OH)), 4.56–4.60 (m, 2H; 2 × CH(OH)), 5.10 (d, *J* = 5.2 Hz; 1H, CH(OH)C(NNHPh)), 6.83–6.88 (m, 2H; 2 × Ar–H), 7.03 (dd, *J*_1_ = 1.2 Hz, *J*_2_ = 8.8 Hz; 2H, 2 × Ar–H), 7.14 (dd, *J*_1_ = 1.2 Hz, *J*_2_ = 8.8 Hz, 2H; 2 × Ar–H), 7.28–7.38 (m, 4H; 4 × Ar–H), 7.87 (s, 1H; CHNNHPh), 10.70 (s, 1H; CHNNHPh), 12.29 (s, 1H; CNNHPh) ppm.

Next, the crude product (7.303 g, 20.4 mmol, 1 equiv) was mixed with the copper sulfate pentahydrate (7.128 g, 28.5 mmol, 1.42 equiv) in water (150 mL). The reaction was refluxed for 2 h and filtered hot after completion, to remove the copper salts. The product was crystallized after cooling to room temperature and filtered with vacuum. As the product was unclean, it was recrystallized from ethanol as a light brown solid (1.76 g, 32.5%). Rf = 0.40 (DCM/MeOH 4:1 + 1% Et_3_N). ^1^H NMR (400 MHz, DMSO-d6): δ = 3.54–3.64 (m, 4H; CH(OH)CH_2_(OH)), 4.39–4.40 (m, 1H; CH(OH)), 4.65–4.69 (m, 2H; CH(OH)CH(OH)), 5.11 (s, 1H; CH(OH)), 5.29 (s, 1H; CH(OH)), 7.23 (s, 1H; Ar–H), 7.38–7.41 (m, 1H; Ar–H), 7.54–7.57 (m, 2H; 2 × Ar–H), 7.95–7.99 (m, 2H; 2 × Ar–H) ppm.

To the product (1.76 g, 6.64 mmol, 1 equiv), dissolved in water (10 mL), sodium metaperiodate (0.85 g, 4.0 mmol, 0.3 equiv) was added and the reaction mixture stirred at the room temperature overnight. Upon the reaction completion, the crystalized crude product was vacuum filtered and washed with diethyl ether, while the mother liquor was washed with DCM (3 × 10 mL), organic phase was dried over Na_2_SO_4_ and concentrated in vacuum. Compound **23** is a dark brown solid (0.282 g, 24.5%). Rf = 0.82 (DCM/MeOH 4:1). ^1^H NMR (400 MHz, DMSO-d6): δ = 7.53–7.57 (m, 1H; Ar–H), 7.63–7.67 (m, 2H; 2 × Ar–H), 8.11–8.13 (m, 2H; 2 × Ar–H), 8.70 (s, 1H; Ar–H), 10.19 (s, 1H; CHCO) ppm.

#### 4.5.9. 1-(2-(6-Methoxy-1,5-naphthyridin-4-yl)ethyl)-N-((2-phenyl-2H-1,2,3-triazol-4-yl)methyl)piperidin-4-amine (**8**)

According to the general procedure for reductive amination, compound **2** (0.20 g, 0.698 mmol, 1 equiv) was used together with the 23 (0.169 g, 0.908 mmol, 1.3 equiv). The residue was purified by flash column chromatography using CHCl_3_/MeOH (9:1) to afford compound **8** (0.134 g, 43.3%) as a dark brown solid. Rf = 0.45 (DCM/MeOH 4:1), 0.22 (CHCl_3_/MeOH 9:1). m.p. 74.1–78.9 °C. ^1^H NMR (400 MHz, CDCl_3_): δ = 1.50–1.58 (m, 2H; N(CH_2_CH_2_)_2_CH) *, 1.99–2.02 (m, 2H; N(CH_2_CH_2_)_2_CH), 2.19–2.24 (m, 2H; N(CH_2_CH_2_)_2_CH), 2.60–2.65 (m, 1H; N(CH_2_CH_2_)_2_CH), 2.79–2.83 (m, 2H; CH_2_CH_2_N), 3.07–3.10 (m, 2H; N(CH_2_CH_2_)_2_CH), 3.38–3.42 (m, 2H; CH_2_CH_2_N), 4.03 (s, 2H; NHCH_2_), 4.09 (s, 3H; CH_3_O), 7.12 (d, *J* = 9.2 Hz, 1H; Ar–H), 7.33–7.37 (m, 1H; Ar–H), 7.43 (d, *J* = 4.4 Hz, 1H; Ar–H), 7.46–7.51 (m, 2H; 2 × Ar–H), 7.75 (s, 1H; Ar–H), 8.04–8.07 (m, 2H; 2 × Ar–H), 8.19 (d, *J* = 9.2 Hz, 1H; Ar–H), 8.67 (d, *J* = 4.4 Hz, 1H; Ar–H) ppm. ^13^C (100 MHz, CDCl_3_): δ = 28.47, 32.69, 41.73, 52.31, 53.72, 54.36, 58.42, 77.23 *, 116.29, 118.72, 124.25, 127.33, 129.27, 134.44, 139.85, 140.35, 140.98, 141.49, 146.72, 147.71, 148.88, 164.41 ppm. IR (ATR): ν = 2915, 2814, 1611, 1593, 1489, 1459, 1399, 1334, 1259, 1131, 1108, 1078, 1019, 990, 966, 851, 807, 756, 713, 692, 662, 603, 585, 545 cm^−1^. HRMS (ESI): *m/z* calculated for C_25_H_30_ON_7_ + H^+^: 444.2506 [M + H]^+^, found: 444.2504. HPLC purity (254 nm): 96.5%. * signal is overlapping with residual solvent peak.

#### 4.5.10. N-((1-Isopropyl-1H-pyrazol-4-yl)methyl)-1-(2-(6-methoxy-1,5-naphthyridin-4-yl)ethyl)piperidin-4-amine (**9**)

According to the general procedure for reductive amination, compound **2** (0.201 g, 0.702 mmol, 1 equiv) was used together with the 1-isopropyl-1*H*-pyrazole-4-carbaldehyde (0.100 mL, 0.782 mmol, 1.1 equiv). The residue was purified by flash column chromatography using CHCl_3_/MeOH (9:1 + 1% Et_3_N) to afford compound **9** (0.201 g, 62.9%) as a light brown viscous liquid. Rf = 0.24 (CHCl_3_/MeOH 9:1 + 1% Et_3_N). ^1^H NMR (400 MHz, CDCl_3_): δ = 1.49 (d, *J* = 6.7 Hz, 6H; 2 × CH(CH_3_)_2_), 1.60–1.74 (m, 2H; N(CH_2_CH_2_)_2_CH), 2.07 (d, *J* = 10.7 Hz, 2H; N(CH_2_CH_2_)_2_CH), 2.30–2.49 (m, 2H; N(CH_2_CH_2_)_2_CH), 2.72–2.84 (m, 1H; N(CH_2_CH_2_)_2_CH), 2.90–3.00 (m, 2H; CH_2_CH_2_N), 3.18 (d, *J* = 11.9 Hz, 2H; N(CH_2_CH_2_)_2_CH), 3.36–3.50 (m, 2H; CH_2_CH_2_N), 3.79 (s, 2H; NHCH_2_), 4.08 (s, 3H; CH_3_O), 4.40–4.54 (m, 1H; CH(CH_3_)_2_) 7.12 (d, *J* = 9.0 Hz, 1H; Ar–H), 7.44 (d, *J* = 4.5 Hz, 1H; Ar–H), 7.46 (s, 1H; Ar–H), 7.48 (s, 1H; Ar–H), 8.19 (d, *J* = 9.0 Hz, 1H; Ar–H), 8.67 (d, *J* = 4.5 Hz, 1H; Ar–H) ppm. ^13^C NMR (100 MHz, CDCl_3_): δ = 18.43, 22.97, 28.47, 29.71, 32.76, 40.98, 52.43, 53.64, 53.70, 54.44, 58.45, 116.26, 119.96, 124.23, 125.22, 138.08, 140.33, 141.00, 141.49, 146.81, 147.69, 161.41 ppm. IR (ATR): ν = 2942, 2814, 2328, 2169, 1613, 1590, 1490, 1439, 1399, 1372, 1334, 1261, 1182, 1122, 1079, 1018, 984, 854, 808, 750, 625, 585, 539 cm^–1^. HRMS (ESI): *m*/*z* calculated for C_23_H_32_N_6_O + H^+^: 409.2710 [M + H]^+^, found: 409.2706. HPLC purity (254 nm): 91.7%. signal is overlapping with residual solvent peak.

#### 4.5.11. N-((1-Allyl-1H-pyrazol-4-yl)methyl)-1-(2-(6-methoxy-1,5-naphthyridin-4-yl)ethyl)piperidin-4-amine (**10**)

According to the general procedure for reductive amination, compound **2** (0.20 g, 0.698 mmol, 1 equiv) was used together with the 1-allyl-1*H*-pyrazole-4-carbaldehyde (0.112 g, 0.823 mmol, 1.2 equiv). The residue was purified by flash column chromatography using CHCl_3_/MeOH (9:1 + 1% Et_3_N) to afford compound **10** (0.200 g, 59.8%) as a dark red viscous liquid. Rf = 0.16 (DCM/MeOH 4:1 + 1% Et_3_N). ^1^H NMR (400 MHz, CDCl_3_): δ = 1.50–1.61 (m, 2H; N(CH_2_CH_2_)_2_CH), 2.00 (d, *J* = 11.1 Hz, 2H; N(CH_2_CH_2_)_2_CH) *, 2.30 (t, *J* = 12.9 Hz, 2H; N(CH_2_CH_2_)_2_CH), 2.60–2.71 (m, 1H; N(CH_2_CH_2_)_2_CH), 2.83–2.91 (m, 2H; CH_2_CH_2_N), 3.11 (d, *J* = 11.8 Hz, 2H; N(CH_2_CH_2_)_2_CH), 3.37–3.46 (m, 2H; CH_2_CH_2_N), 3.74 (s, 2H; NHCH_2_), 4.08 (s, 3H; CH_3_O), 4.71 (m, 2H; CH_2_CH=CH_2_), 5.19–5.31 (m, 2H; CH_2_CH=CH_2_), 6.01 (m, 1H; CH_2_CH=CH_2_), 7.12 (d, *J* = 9.0 Hz, 1H; Ar–H), 7.43 (s, 3H; 3 × Ar–H), 8.19 (d, *J* = 9.0 Hz, 1H; Ar–H), 8.66 (d, *J* = 4.5 Hz, 1H; Ar–H) ppm. ^13^C NMR (100 MHz, CDCl_3_): δ = 28.43, 32.46, 40.64, 44.04, 46.12, 52.32, 53.69, 54.15, 54.73, 58.37, 116.27, 118.65, 120.31, 124.22, 128.00, 132.90, 138.93, 140.28, 140.96, 141.44, 146.72, 147.64, 161.40 ppm. IR (ATR): ν = 2938, 2805, 2324, 2167, 1612, 1589, 1489, 1439, 1398, 1372, 1333, 1260, 1119, 1077, 1018, 990, 928, 853, 807, 731, 642, 585 cm^–1^. HRMS (ESI): *m*/*z* calculated for C_23_H_30_N_6_O + H^+^: 407.2554 [M + H]^+^, found: 407.2547. HPLC purity (254 nm): 96.0%. * signal is overlapping with residual solvent peak.

#### 4.5.12. N-(4-(Difluoromethoxy)-3-methoxybenzyl)-1-(2-(6-methoxy-1,5-naphthyridin-4-yl)ethyl)piperidin-4-amine (**19**)

According to the general procedure for reductive amination, compound **2** (0.194 g, 0.677 mmol, 1 equiv) was used together with the 4-(difluoromethoxy)-3-methoxybenzaldehyde (0.120 mL, 0.748 mmol, 1.1 equiv). The residue was purified by flash column chromatography using CHCl_3_/MeOH (9:1 + 1% Et_3_N) to afford compound **19** (0.194 g, 54.9%) as a red-brown solid. Rf = 0.19 (DCM/MeOH 4:1 + 1% Et_3_N). m.p. 85–93 °C. ^1^H NMR (400 MHz, CDCl_3_): δ = 1.44–1.55 (m, 2H; N(CH_2_CH_2_)_2_CH), 1.96 (d, *J* = 11.6 Hz, 2H; N(CH_2_CH_2_)_2_CH), 2.19 (t, *J* = 10.5 Hz, 2H; N(CH_2_CH_2_)_2_CH), 2.48–2.59 (m, 1H; N(CH_2_CH_2_)_2_CH), 2.72–2.84 (m, 2H; CH_2_CH_2_N), 3.06 (d, *J* = 11.7 Hz, 2H; N(CH_2_CH_2_)_2_CH), 3.34–3.41 (m, 2H; CH_2_CH_2_N), 3.81 (s, 2H; NHCH_2_), 3.89 (s, 3H; CH_3_O), 4.07 (s, 3H; CH_3_O), 6.53 (t, *J* = 75.4 Hz, 1H; OCHF_2_), 6.88 (dd, *J*_1_ = 8.1 Hz, *J*_2_ = 1.8 Hz, 1H; Ar–H), 7.01 (d, *J* = 1.6 Hz, 1H; Ar–H), 7.10 (d, *J* = 8.4 Hz, 1H; Ar–H), 7.11 (d, *J* = 8.8 Hz, 1H; Ar–H), 7.41 (d, *J* = 4.5 Hz, 1H; Ar–H), 8.18 (d, *J* = 9.0 Hz, 1H; Ar–H), 8.66 (d, *J* = 4.5 Hz, 1H; Ar–H) ppm. ^13^C NMR (100 MHz, CDCl_3_): δ = 28.45, 32.79, 50.51, 52.36, 53.71, 55.91, 58.41, 77.23 *, 112.34, 116.25 (t, *J* = 259.5 Hz), 116.29, 120.21, 122.22, 124.25, 138.73, 139.72, 140.37, 140.97, 141.50, 146.62, 147.72, 151.08, 161.42 ppm. IR (ATR): ν = 2937, 2832, 1610, 1592, 1507, 1490, 1465, 1419, 1398, 1374, 1334, 1264, 1212, 1191, 1079, 1034, 1015, 853, 811, 787, 753, 732, 586 cm^–1^. HRMS (ESI): *m*/*z* calculated for C_25_H_30_F_2_N_4_O_3_ + H^+^: 473.2359 [M + H]^+^, found: 473.2354. HPLC purity (254 nm): 97.2%. * signal is overlapping with residual solvent peak.

#### 4.5.13. 2-(Difluoromethoxy)-5-((1-(2-(6-methoxy-1,5-naphthyridin-4-yl)ethyl)piperidin-4-ylamino)methyl)phenol (**20**)

According to the general procedure for reductive amination, compound **2** (0.171 g, 0.597 mmol, 1 equiv) was used together with the 4-(difluoromethoxy)-3-hydroxybenzaldehyde (0.138 g, 0.734 mmol, 1.2 equiv). The residue was purified by flash column chromatography using CHCl_3_/MeOH (9:1 + 1% Et_3_N) to afford compound **20** (0.171 g, 50.9%) as a light brown solid. Rf = 0.09 (DCM/MeOH 4:1 + 1% Et_3_N). m.p. 99–104 °C. ^1^H NMR (400 MHz, CDCl_3_): δ = 1.48–1.57 (m, 2H; N(CH_2_CH_2_)_2_CH), 1.98 (d, *J* = 11.2 Hz, 2H; N(CH_2_CH_2_)_2_CH), 2.18–2.24 (m, 2H; N(CH_2_CH_2_)_2_CH), 2.55–2.61 (m, 1H; N(CH_2_CH_2_)_2_CH), 2.78–2.86 (m, 2H; CH_2_CH_2_N), 3.08 (d, *J* = 11.8 Hz, 2H; N(CH_2_CH_2_)_2_CH), 3.36–3.45 (m, 2H; CH_2_CH_2_N), 3.78 (s, 2H; NHCH_2_), 4.10 (s, 3H; CH_3_O), 6.53 (t, *J* = 74.0 Hz, 1H; OCHF_2_), 6.85 (dd, *J*_1_ = 8.2 Hz, *J*_2_ =2.0 Hz, 1H; Ar–H), 7.02 (d, *J* = 2.0 Hz, 1H; Ar–H), 7.06 (d, *J* = 8.2 Hz, 1H; Ar–H), 7.14 (d, *J* = 9.0 Hz, 1H; Ar–H), 7.44 (d, *J* = 4.4 Hz, 1H; Ar–H), 8.21 (d, *J* = 9.0 Hz, 1H; Ar–H), 8.68 (d, *J* = 4.5 Hz, 1H; Ar–H) ppm. ^13^C NMR (100 MHz, CDCl_3_): δ = 28.12, 31.72, 49.94, 51.96, 53.77, 58.06, 77.23 *, 116.38 (t, *J* = 260.8 Hz), 116.46, 117.32, 119.91, 120.53, 124.34, 137.80, 138.54, 140.22, 140.89, 141.38, 146.10, 147.61, 148.08, 161.53 ppm. IR (ATR): ν = 2944, 2338, 1612, 1595, 1490, 1438, 1399, 1334, 1297, 1261, 1113, 1076, 1049, 1014, 851, 809, 782, 752, 714, 674, 641, 586 cm^–1^. HRMS (ESI): *m/z* calculated for C_24_H_28_F_2_N_4_O_3_ + H^+^: 459.2202 [M + H]^+^, found: 459.2196. HPLC purity (254 nm): 97.9%. * signal is overlapping with residual solvent peak.

#### 4.5.14. 1-(2-(6-Methoxy-1,5-naphthyridin-4-yl)ethyl)-N-((6-(trifluoromethyl)pyridin-3-yl)methyl)piperidin-4-amine (**21**)

According to the general procedure for reductive amination, compound **2** (0.184 g, 0.643 mmol, 1 equiv) was used together with the 6-(trifluoromethyl)nicotinaldehyde (0.118 g, 0.674 mmol, 1.05 equiv). The residue was purified by flash column chromatography using CHCl_3_/MeOH (9:1 + 1% Et_3_N) to afford compound **21** (0.184 g, 61.28%) as a red-brown solid. Rf = 0.22 (CHCl_3_/MeOH 9:1 + 1% Et_3_N). m.p. 75–78 °C. ^1^H NMR (400 MHz, CDCl_3_): δ = 1.43–1.54 (m, 2H; N(CH_2_CH_2_)_2_CH), 1.95 (d, *J* = 12.1 Hz, 2H; N(CH_2_CH_2_)_2_CH), 2.17 (t, *J* = 10.9 Hz, 2H; N(CH_2_CH_2_)_2_CH), 2.50–2.57 (m, 1H; N(CH_2_CH_2_)_2_CH), 2.76–2.83 (m, 2H; CH_2_CH_2_N), 3.05 (d, *J* = 11.8 Hz, 2H; N(CH_2_CH_2_)_2_CH), 3.35–3.41 (m, 2H; CH_2_CH_2_N), 3.94 (s, 2H; NHCH_2_), 4.07 (s, 3H; CH_3_O), 7.11 (d, *J* = 9.0 Hz, 1H; Ar–H), 7.41 (d, *J* = 4.5 Hz, 1H; Ar–H), 7.65 (d, *J* = 8.0 Hz, 1H; Ar–H), 7.91 (dd, *J*_1_ = 8.0 Hz, *J*_2_ = 1.4 Hz, 1H; Ar–H), 8.18 (d, *J* = 9.0 Hz, 1H; Ar–H), 8.66 (d, *J* = 4.5 Hz, 1H; Ar–H), 8.69 (d, *J* = 1.1 Hz, 1H; Ar–H) ppm. ^13^C NMR (100 MHz, CDCl_3_): δ = 28.46, 32.78, 47.65, 52.26, 53.70, 58.38, 77.23 *, 116.31, 120.22 (q, *J* = 264.1 Hz), 121.64, 124.22, 136.86, 139.76, 140.34, 140.95, 141.48, 146.60, 146.88, 147.69, 149.72, 161.42 ppm. IR (ATR): ν = 2946, 2796, 1612, 1590, 1490, 1449, 1400, 1373, 1334, 1262, 1172, 1119, 1083, 1014, 980, 837, 799, 751, 631, 586 cm^–1^. HRMS (ESI): *m*/*z* calculated for C_23_H_26_F_3_N_5_O + H^+^: 446.2162 [M + H]^+^, found: 446.2157. HPLC purity (254 nm): 99.4%. * signal is overlapping with residual solvent peak.

#### 4.5.15. 1-(2-(6-Methoxy-1,5-naphthyridin-4-yl)ethyl)-N-((6-methoxypyridin-3-yl)methyl)piperidin-4-amine (**22**)

According to the general procedure for reductive amination, compound **2** (0.20 g, 0.698 mmol, 1 equiv) was used together with the 6-methoxynicotinaldehyde (0.125 g, 0.908 mmol, 1.3 equiv). The residue was purified by flash column chromatography using CHCl_3_/MeOH (9:1) to afford compound **22** (0.187 g, 65.7%) as a yellow-orange solid. Rf = 0.20 (DCM/MeOH 4:1), 0.11 (CHCl_3_/MeOH 9:1). m.p. 67–69 °C. ^1^H NMR (400 MHz, CDCl_3_): δ = 1.44–1.54 (m, 2H; N(CH_2_CH_2_)_2_CH), 1.95–1.98 (m, 2H; N(CH_2_CH_2_)_2_CH), 2.17–2.22 (m, 2H; N(CH_2_CH_2_)_2_CH), 2.52–2.57 (m, 1H; N(CH_2_CH_2_)_2_CH), 2.78–2.82 (m, 2H; CH_2_CH_2_N), 3.05–3.08 (m, 2H; N(CH_2_CH_2_)_2_CH), 3.38–3.42 (m, 2H; CH_2_CH_2_N), 3.78 (s, 2H; NHCH_2_), 3.95 (s, 3H; CH_3_O), 4.09 (s, 3H; CH_3_O), 6.75 (dd, *J*_1_ = 8.4 Hz, *J*_2_ = 0.4 Hz, 1H; Ar–H), 7.13 (d, *J* = 9.2 Hz, 1H; Ar–H), 7.43 (d, *J* = 4.4 Hz, 1H; Ar–H), 7.61 (dd, *J*_1_ = 8.4 Hz, *J*_2_ = 2.4 Hz, 1H; Ar–H), 8.10 (d, *J* = 1.6 Hz, 1H; Ar–H), 8.20 (d, *J* = 9.2 Hz, 1H; Ar–H), 8.68 (d, *J* = 4.4 Hz, 1H; Ar–H) ppm. ^13^C (100 MHz, CDCl_3_): δ = 28.42, 32.65, 47.49, 52.26, 53.42, 53.72, 58.39, 77.24 *, 110.77, 116.30, 124.25, 128.67, 140.35, 139.00, 140.97, 141.49, 146.08, 146.62, 147.71, 161.42, 163.47 ppm. IR (ATR): ν = 2929, 2808, 1610, 1591, 1449, 1461, 1397, 1376, 1334, 1313, 1292, 1256, 1107, 1073, 847, 770 cm^−1^. HRMS (ESI): *m*/*z* calculated for C_23_H_30_O_2_N_5_ + H^+^: 408.2394 [M + H]^+^, found: 408.2391. HPLC purity (254 nm): 98.00%. * signal is overlapping with residual solvent peak.

### 4.6. In Vitro DNA Gyrase Inhibitory Activity

A Gyrase Supercoiling High Throughput Plate assay kit, purchased from Inspiralis (Norwich. UK) was used to determine IC_50_ values of compounds on *S. aureus* and *E. coli*. The assay was performed on black streptavidin-coated 96-well microtiter plates (Thermo Scientific Pierce), starting by wells rehydration with supplied wash buffer (20 mM Tris-HCl (pH 7.6), 137 mM NaCl, 0.005% (*w*/*v*) BSA, 0.05% (*v*/*v*) Tween 20). Biotinylated oligonucleotide in wash buffer was immobilized onto the wells, and the excess oligonucleotide was washed off with wash buffer and ultrapure water. 1.5 U of *S. aureus* or *E. coli* DNA gyrase enzyme was incubated together with 0.75 μg of relaxed pNO1 plasmid as a substrate in the presence of 3 μL inhibitor solution in 10% DMSO and 0.008% Tween 20 at 37 °C for 30 min in a final reaction volume of 30 μL in buffer (40 mM HEPES, KOH (pH 7.6), 10 mM magnesium acetate, 10 mM DTT, 2 mM ATP, 500 mM potassium glutamate, 0.05 mg/mL albumin for *S. aureus* and 35 mM Tris-HCl (pH 7.5), 24 mM KCl, 4 mM MgCl_2_, 2 mM DTT, 1.8 mM spermidine, 1 mM ATP, 6.5% (*w*/*v*) glycerol, and 0.1 mg/mL albumin for *E. coli*). The reactions were stopped by adding the TF buffer (50 mM NaOAc (pH 4.7), 50 mM NaCl, and 50 mM MgCl_2_) to allow the triplex formation (biotin-oligonucleotide-plasmid) for additional 30 min. Afterward, the unbound plasmid was washed off using TF buffer, and the solution of Promega Diamond dye in T10 buffer (10 mM Tris-HCl (pH 8.0), 1 mM EDTA) was added. 10 min after, the solution was mixed and the fluorescence was read using Tecan Fluorimeter (excitation, 495 nm; emission, 537 nm). Preliminary screening was performed at four inhibitor concentrations of 100, 10, 1, and 0.1 μM. The IC_50_ values were determined at seven inhibitor concentrations for the compounds that showed residual enzyme activity at concentration 100 μM less than 50%, whereas the other compounds were noted as inactive (IC_50_ > 100 μM). The concentration of inhibitor where the residual activity of the enzyme is 50% (IC_50_) was calculated using nonlinear regression based fitting of inhibition curves using log [inhibitor] versus response-variable slope (four parameters)–symmetrical equation in GraphPad Prism 6.0 software (GraphPad Software, La Jolla, CA, USA, www.graphpad.com, accessed on 13 July 2021). IC_50_ value represents the mean of two to four independent measurements. Ciprofloxacin was used as a positive control, showing IC_50_ 93.65 μM and 0.44 μM for *S. aureus* and *E. coli*, respectively. IC_50_ determination of compounds **14** and **15** was performed by Inspiralis (Norwich, UK) using their gel-based *S. aureus* Gyrase Supercoiling assay, due to insufficient sensitivity of fluorescence-based High Throughput Supercoiling assay and is described elsewhere [13].

### 4.7. In Vitro Topo IV Inhibitory Activity

A Topo IV Relaxation High Throughput Plate assay kit, purchased from Inspiralis (Norwich. UK) was used to determine IC_50_ values of compounds on *S. aureus* and *E. coli*. The assay was performed on black streptavidin-coated 96-well microtiter plates (Thermo Scientific Pierce, Thermo Fisher Scientific, Waltham, MA, USA), starting by wells rehydration with supplied wash buffer (20 mM Tris-HCl (pH 7.6), 137 mM NaCl, 0.005% (*w*/*v*) BSA, 0.05% (*v*/*v*) Tween 20). Biotinylated oligonucleotide in wash buffer was immobilized onto the wells, and the excess oligonucleotide was washed off with wash buffer and ultrapure water. 1.5 U of *S. aureus* or *E. coli* DNA gyrase enzyme was incubated together with 0.75 μg of supercoiled pNO1 plasmid as a substrate in the presence of 3 μL inhibitor solution in 10% DMSO and 0.008% Tween 20 at 37 °C for 30 min in a final reaction volume of 30 μL in dilution buffer (50 mM Tris-HCl (pH 7.5), 1 mM DTT, 1 mM EDTA, 40% (*w*/*v*) glycerol for *S. aureus* and 40 mM HEPES, KOH (pH 7.6), 100 mM potassium glutamate, 1 mM DTT, 1 mM EDTA, and 40% (*v*/*v*) glycerol for *E. coli*). The reactions were stopped by adding the TF buffer (50 mM NaOAc (pH 4.7), 50 mM NaCl, and 50 mM MgCl_2_) to allow the triplex formation (biotin-oligonucleotide-plasmid) for additional 30 min. Afterward, the unbound plasmid was washed off using TF buffer, and the solution of Promega Diamond dye in T10 buffer (10 mM Tris-HCl (pH 8.0), 1 mM EDTA) was added. Ten minutes after, the solution was mixed and the fluorescence was read using Tecan Fluorimeter (excitation, 495 nm; emission, 537 nm). Preliminary screening was performed at two inhibitor concentrations of 100 and 1 μM. The IC_50_ values were determined at seven inhibitor concentrations for the compounds that showed residual enzyme activity at concentration 100 μM less than 50%, whereas the other compounds were noted as inactive (IC_50_ > 100 μM). The concentration of inhibitor where the residual activity of the enzyme is 50% (IC_50_) was calculated using nonlinear regression based fitting of inhibition curves using log [inhibitor] versus response-variable slope (four parameters)–symmetrical equation in GraphPad Prism 6.0 software (GraphPad Software, La Jolla, CA, USA, www.graphpad.com, accessed on 13 July 2021). IC_50_ value represents the mean of two to four independent measurements. Ciprofloxacin was used as a positive control, showing IC_50_ 26.25 μM and 8.07 μM for *S. aureus* and *E. coli*, respectively.

### 4.8. Human Topo II Selectivity Evaluation

A Human topoisomerase II Alpha Relaxation High Throughput Plate assay kit, purchased from Inspiralis (Norwich, UK) was used to evaluate the selectivity of compounds for the bacterial enzymes over the homologous human topoisomerase II. The assay was performed on black streptavidin-coated 96-well microtiter plates (Thermo Scientific Pierce), starting by wells rehydration with supplied wash buffer (20 mM Tris-HCl (pH 7.6), 137 mM NaCl, 0.005% (*w*/*v*) BSA, 0.05% (*v*/*v*) Tween 20). Biotinylated oligonucleotide in wash buffer was immobilized onto the wells and the excess oligonucleotide was washed off with wash buffer. 1.5 U of Human topoisomerase IIα enzyme was incubated together with 0.75 μg of supercoiled pNO1 plasmid as a substrate in the presence of 3 μL inhibitor solution in 10% DMSO and 0.008% Tween 20, and 1 μL of 30 mM ATP, at 37 °C for 30 min in a final reaction volume of 30 μL in buffer (50 mM Tris-HCl (pH 7.5), 125 mM NaCl, 10 mM MgCl_2_, 5 mM DTT, and 100 μg/mL albumin). The reactions were stopped by adding the TF buffer (50 mM NaOAc (pH 4.7), 50 mM NaCl, and 50 mM MgCl_2_) to allow the triplex formation (biotin-oligonucleotide-plasmid) for additional 30 min. Afterward, the unbound plasmid was washed off using TF buffer, and the solution of Promega Diamond dye in T10 buffer (10 mM Tris-HCl (pH 8.0), 1 mM EDTA) was added. Ten minutes after, the solution was mixed and the fluorescence was read using Tecan Fluorimeter (excitation, 495 nm; emission, 537 nm). Screening was performed at inhibitor concentrations of 10 μM. Raw data were converted to mean ± SD percentage of residual activity of the enzyme obtained by two independent measurements.

### 4.9. Antimicrobial Susceptibility Testing

Antimicrobial testing was carried out by the broth microdilution method in 96-well plate format following the Clinical and Laboratory Standards Institute (CLSI) guidelines [40] and European Committee on Antimicrobial Susceptibility Testing (EUCAST) recommendations [41]. Bacterial suspension of specific bacterial strain equivalent to 0.5 McFarland turbidity standard was diluted with cation-adjusted Mueller Hinton broth with TES (Thermo Fisher Diagnostic, Landsmeer, The Netherlands), to obtain a final inoculum of 10^5^ CFU/mL. Compounds dissolved in DMSO and inoculum were mixed together and incubated for 18–20 h at 35 °C. Suspensions of *Mycobacterium* sp. were prepared into a tube of Middelbrook 7H9 broth supplemented with 5% OADC (Thermo Scientific, Trek Diagnostic Systems, West Sussex, UK) and incubated for at least 10 days. After incubation the minimal inhibitory concentration (MIC) values were determined by visual inspection as the lowest dilution of compounds showing no turbidity. Tetracycline was used as a positive control on every assay plate.

### 4.10. Metabolic Activity Assessment

Cells of the HepG2 line (ATCC^®^ HB-8065™) and HUVEC (ATCC^®^ CRL-1730™) were cultured in Dulbecco’s Modified Eagle Medium (DMEM; Sigma-Aldrich, St. Louis, MO, USA) supplemented with 10% heat-deactivated FBS (Gibco, Grand Island, NE, USA), 2 mM L-glutamine, 100 U/mL penicillin, 100 μg/mL streptomycin (all from Sigma-Aldrich, St. Louis, MO, USA) in a humidified chamber at 37 °C and 5% CO_2_. The experiments were carried out on passages 7–9 for both cell lines. The tested compounds were dissolved in DMSO and further diluted in culture medium to a desired final concentration. Cells were seeded into 96-well plates at 8 × 10^4^ cells/mL (100 μL/well) and incubated for 24 h to attach onto the wells. Cells were treated with 1 μM and 50 μM of each compound of interest or corresponding vehicle as control. For IC_50_ determination, cells were seeded into 96-well plates at 5 × 10^4^ cells/mL (100 μL/well) and after attachment treated with seven different concentration of the selected compounds or corresponding vehicle as control. The metabolic activity was assessed after 72 h treatment using the CellTiter 96 Aqueous One Solution Cell Proliferation Assay (Promega, Madison, WI, USA), in accordance with the manufacturer’s instructions [42]. The absorbance was measured at 492 nm on an automated microplate reader BioTek Synergy HT (BioTek Instruments, Inc., Winooski, VT, USA). Data are presented as the percentage of metabolic activity of control cells stimulated with vehicle or as IC_50_ value (mean ± SD) from two independent experiments, each conducted in duplicate or triplicate. IC_50_ values were calculated by a non-linear regression model using Graph Pad Prism 7 software.

### 4.11. Cardiovascular hERG Inhibition

Cardiovascular safety profile was entirely outsourced. hERG screening was performed at TCG Lifesciences Pvt. Ltd., Kolkata, West Bengal, India with FP-based hERG binding assay (Invitrogen kit).

## 5. Patents

Antibacterials based on monocyclic fragments coupled to aminopiperidine naphthyridine scaffold: WO 2020169593 A1. Munich: European Patent Organisation (EPO).

Antibacterials based on monocyclic fragments coupled to aminopiperidine naphthyridine scaffold: LU101131. Luxembourg: Office de la propriété intellectuelle Ministère de lʹÉconomie.

## Figures and Tables

**Table 1:** DNA supercoiling inhibition of *S. aureus* and *E. coli* DNA gyrase and topoisomerase IV and decatenation activity of human topoisomerase IIα affected by NBTIs.

Cmpd	Structure	DNA Gyrase IC_50_ (μM) ^1^		Topoisomerase IV IC_50_ (μM) ^1^		Human Topo II (% RA) ^2^
		*S. aureus*	*E. coli*	*S. aureus*	*E. coli*	
**3**	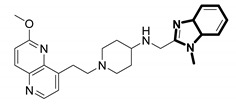	1.60 ± 0.35	>100	>100	1.18 ± 0.09	94.71 ± 4.82
**4**	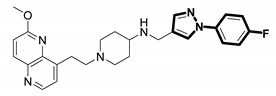	0.51 ± 0.25	13.27 ± 2.84	>100	0.12 ± 0.01	88.41 ± 0.15
**5**	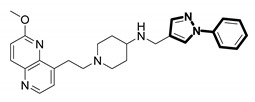	0.33 ± 0.08	3.00 ± 0.55	29.59 ± 0.54	0.085 ± 0.002	82.10 ± 2.52
**6**	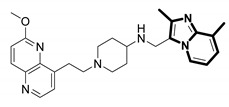	>100	>100	>100	>100	99.36 ± 9.93
**7**	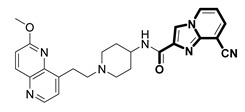	>100	>100	>100	>100	90.35 ± 7.56
**8**	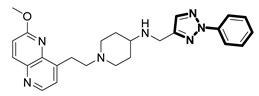	0.24 ± 0.04	7.83 ± 0.55	>100	0.21 ± 0.00	94.17 ± 2.90
**9**	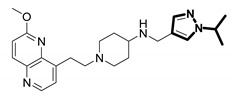	0.16 ± 0.01	2.95 ± 0.56	40.38 ± 0.09	0.23 ± 0.00	101.25 ± 6.82
**10**	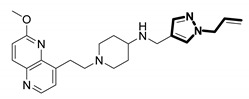	1.10 ± 0.17	16.70 ± 0.93	>100	0.28 ± 0.01	95.69 ± 6.42
**11**	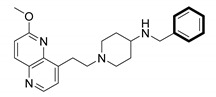	1.02 ± 0.02	40.60 ± 1.97	>100	2.04 ± 0.20	96.97 ± 9.58
**12**	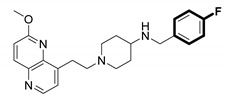	0.55 ± 0.06	16.95 ± 1.69	>100	0.28 ± 0.03	100.81 ± 11.22
**13**	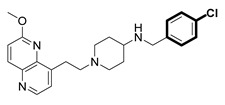	0.035 ± 0.01	1.71 ± 0.05	16.37 ± 0.56	0.031 ± 0.00	101.98 ± 0.84
**14**	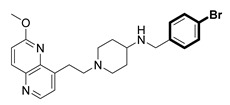	0.007 ± 0.00 ^3^	0.57 ± 0.06	7.22 ± 0.55	0.042 ± 0.003	98.25 ± 2.72
**15**	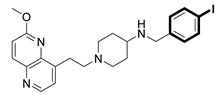	0.011 ± 0.003 ^3^	0.28 ± 0.02	2.13 ± 0.11	0.021 ± 0.001	88.26 ± 3.33
**16**	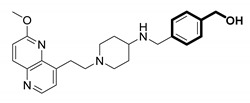	4.39 ± 0.55	56.56 ± 3.32	>100	1.21 ± 0.05	97.73 ± 1.08
**17**	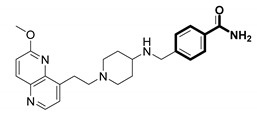	1.82 ± 0.11	>100	>100	1.15 ± 0.03	97.80 ± 2.21
**18**	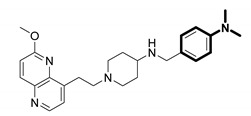	0.067 ± 0.01	11.89 ± 0.83	>100	>100	94.19 ± 1.33
**19**	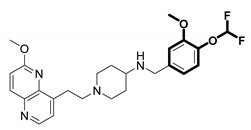	0.35 ± 0.03	3.90 ± 0.06	29.04 ± 1.89	0.10 ± 0.01	96.36 ± 2.30
**20**	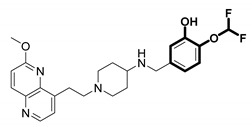	0.33 ± 0.02	4.41 ± 0.16	>100	0.06 ± 0.00	94.80 ± 0
**21**	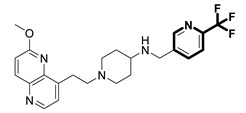	0.16 ± 0.02	6.91 ± 1.62	18.45 ± 1.32	>100	95.49 ± 2.39
**22**	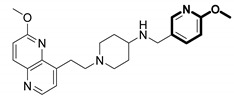	0.34 ± 0.05	7.24 ± 0.34	>100	>100	97.05 ± 6.94
Gepotidacin	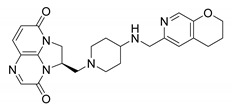	0.37 ± 0.02	0.24 ± 0.04	8.30 ± 0.37	0.05 ± 0.00	ND

^1^ IC_50_ value represents the mean value of two to four independent measurements. ^2^ The mean ± SD percentage of residual activity of the enzyme at compound concentration 10 μM obtained by two independent measurements. ^3^ IC_50_s were determined by using gel-based assays, as detailed in [13].

**Table 2:** Antimicrobial susceptibility of NBTIs against a panel of Gram-positive, Gram-negative, and *Mycobacterium* bacterial strains.

Compd	3	4	5	6	7	8	9	10	11	12	13	14	15	16	17	18	19	20	21	22	Gepotidacin
MIC [μg/mL]																					
*S. aureus* (ATCC 29213)	1	2	0.25	>128	>128	0.5	0.125	2	4	1	0.125	0.031	0.0078	4	2	0.125	1	0.5	0.25	0.5	0.125
*MRSA*	2	2	0.5	>128	>128	0.5	0.25	2	8	2	0.25	0.062	0.015	4	4	0.25	2	1	0.5	1	ND
*MRSA* ^1^ (QA-11.7)	8	2	0.5	>128	>128	0.5	1	4	8	4	0.5	0.0625	0.0078	4	4	0.5	2	0.5	1	1	ND
*MRSA* ^2^ (QA-11.6)	2	4	1	>128	>128	0.5	1	4	8	4	0.25	0.0625	0.03125	4	4	0.25	2	1	0.5	2	ND
*MRSA* ^3^ (QA-11.4)	4	8	1	>128	>128	1	1	4	8	4	0.5	0.125	0.0625	8	8	0.5	2	2	1	2	ND
*MRSA* ^4^ (QA-11.2)	4	8	1	>128	>128	0.5	1	4	8	4	0.25	0.125	0.03125	8	8	0.5	2	1	0.5	2	ND
*E. faecalis DRK 057* (ATCC 29212)	4	8	1	>128	>128	1	1	8	16	8	1	1	1	8	128	1	16	4	2	2	ND
*S. pneumoniae* (QA-10/02)	2	4	0.5	>128	128	0.25	0.5	1	2	2	0.5	0.125	0.015	2	16	0.25	2	1	1	1	ND
*S. agalactiae RDK 047* (QA-990/02)	<1	2	1	128	>128	0.5	0.5	4	16	8	1	0.5	0.125	8	>128	0.25	<1	2	4	1	ND
*E. coli* (ATCC 25922)	>128	32	16	>128	>128	64	8	16	64	32	4	2	2	128	>128	32	32	8	32	16	1
*E. coli* ^5^ (QA-11.8)	64	16	2	>128	>128	32	8	16	64	16	2	1	0.25	128	>128	8	4	4	8	8	ND
*E. coli* ^6^ (QA-11.7)	64	16	2	>128	>128	4	2	4	32	8	1	1	1	32	128	4	2	2	2	2	ND
*E. coli* ^7^ (QA-11.3)	128	8	2	128	>128	32	4	8	32	8	2	1	1	32	128	8	4	1	8	4	ND
*E. coli D22* ^8^	>128	16	1	>128	>128	32	1	8	16	16	2	0.125	0.125	16	128	8	16	2	4	4	0.125
*E. coli N43* ^9^ (CGSC# 5583)	16	2	1	128	>128	1	0.25	1	8	4	0.5	0.125	0.078	16	16	1	1	0.5	1	1	0.016
*A. baumannii 8C6* (GES-14)	>128	32	4	>128	>128	32	2	32	64	32	4	1	0.25	128	128	8	8	4	16	4	ND
*P. aeruginosa RDK 184* (DSM 939)	>128	>128	>128	>128	>128	>128	>128	>128	>128	>128	>128	128	32	>128	>128	>128	>128	>128	>128	>128	ND
*S. alachua RDK 030c* (QA-1482/04)	>128	128	64	>128	>128	>128	64	128	>128	128	32	16	8	>128	>128	128	128	64	128	64	ND
*C. jejuni RDK 135* (ATCC 33560)	1	128	16	>128	>128	4	0.5	8	32	1	2	0.5	0.015	32	128	4	2	0.015	128	2	ND
*M. smegmatis*	4	≤1	0.25	64	16	0.5	0.25	≤1	8	2	0.25	0.125	0.015	32	16	4	4	0.5	0.25	16	ND
*M. avium*	>128	128	64	>128	>128	32	128	>128	>128	32	4	2	1	>128	128	128	128	128	4	>128	ND

QA-labeled isolates were obtained from quality assurance schemes sent by reference laboratories. ^1^ Resistant to: cefoxitin, ciprofloxacin, clindamycin, erythromycin, tetracycline, thiamulin, trimethoprim. ^2^ Resistant to: cefoxitin, gentamicin, sulfamethoxazole, tetracycline. ^3^ Resistant to: cefoxitin. ^4^ Resistant to: cefoxitin, chloramphenicol, clindamycin, linezolid, tetracycline, thiamulin, trimethoprim. ^5^ Resistant to: ampicillin, cefotaxime, ceftazidime, ciprofloxacin, meropenem, nalidixic acid, sulfamethoxazole, tetracycline, trimeptoprim, cefepim, cefoxitn, ertapenem, imipenem. ^6^ Resistant to: ampicillin, azithromycin, cefotaxime, ceftazidime, sulfamethoxazole, trimeptoprim, cefepim, cefoxitin, ertapenem, imipenem. ^7^ Resistant to: ampicillin, cefotaxime, ceftazidime, chloramphenicol, ciprofloxacin, colistin, gentamicin, meropenem, nalidixic acid, sulfamethoxazole, tetracycline, trimeptoprim, cefepim, cefoxitin, imipenem. ^8^ Bears a mutation in the lpxC gene that increases membrane permeability. ^9^ AcrA knockout strain (knockout of cell membrane efflux pump). ND: not determined.

**Table 3:** Calculated ligand efficiency (LE) values of compounds.

	LE ^1^			
Cmpd	DNA Gyrase		Topo IV	
	*S. aureus*	*E. coli*	*S. aureus*	*E. coli*
**3**	0.25	NA	NA	0.25
**4**	0.25	0.20	NA	0.28
**5**	0.27	0.23	0.19	0.29
**6**	NA	NA	NA	NA
**7**	NA	NA	NA	NA
**8**	0.27	0.21	NA	0.28
**9**	0.31	0.25	0.20	0.30
**10**	0.27	0.22	NA	0.30
**11**	0.29	0.21	NA	0.28
**12**	0.30	0.23	NA	0.31
**13**	0.35	0.27	0.23	0.35
**14**	0.39	0.29	0.24	0.35
**15**	0.38	0.31	0.27	0.36
**16**	0.24	0.19	NA	0.27
**17**	0.25	NA	NA	0.26
**18**	0.32	0.22	NA	NA
**19**	0.26	0.22	0.18	0.28
**20**	0.27	0.22	NA	0.30
**21**	0.29	0.22	0.20	NA
**22**	0.30	0.23	NA	NA

^1^ The LE was calculated according to the following equation: LE = (1.37/HA) × pIC_50_, where HA is the number of non-hydrogen (heavy) atoms [26]. NA: not applicable.

**Table 4:** Results of cytotoxicity on human HUVEC and HepG2 cell lines and hERG-related cardiotoxicity.

Cmpd	HUVEC	HepG2	hERG
	IC_50_ [μM]	IC_50_ [μM]	IC_50_ [μM]
**3**	>50	Nd	1.04
**4**	9.64 ± 0.03	4.64 ± 0.10	0.33
**5**	10.70 ± 0.01	6.33 ± 0.19	0.62
**6**	>50	ND	ND
**7**	ND	ND	ND
**8**	39.41 ± 5.47	17.08 ± 0.98	0.37
**9**	>50	>50	9.82
**10**	>50	>50	6.62
**11**	54.08 ± 3.45	17.67 ± 2.16	1.05
**12**	>50	12.67± 0.21	0.59
**13**	26.96 ± 0.38	10.16 ± 0.10	0.27
**14**	25.42 ± 0.01	9.37 ± 0.07	0.29
**15**	18.98 ± 0.85	8.47 ± 0.09	0.18
**16**	>50	ND	3.24
**17**	>50	ND	13.45
**18**	23.85 ± 0.09	10.03 ± 0.06	2.11
**19**	19.83 ± 2.58	6.31 ± 0.09	0.46
**20**	29.80 ± 1.13	10.86 ± 0.12	0.18
**21**	>50	>50	1.97
**22**	>50	32.03 ± 0.30	3.05

ND: not determined.

## Data Availability

Not applicable.

## Supplementary Materials

The following are available online at https://www.mdpi.com/article/10.3390/antibiotics10070862/s1, Figure S1: Predicted binding modes of compound **13**–**15** within the *S. aureus* and *E. coli* DNA gyrase NBTIs binding site, Figure S2: Predicted binding modes of compound **13**–**15** within the *S. aureus* and *E. coli* Topo IV NBTIs binding site, Figure S3: Differences in amino acid residues constituting NBTIs’ binding pocket of DNA gyrase and Topo IV, Scheme S1: Synthesis of 2-phenyl-2*H*-1,2,3-triazole-4-carbaldehyde (**23**), NMR spectra, Table S1: The HPLC method parameters, equilibrium solubility at pH 6.8 and PAMPA permeability of our NBTIs and reference compounds, Table S2: Comparison of *S. aureus* DNA gyrase IC_50_ and the lengths of RHS fragments for our previously published compound **13** and known NBTIs, Table S3: Selected templates for homology modelling, Table S4. Minimal inhibitory concentration (MIC) in molar, Table S5. The metabolic activity of cells at 1 μM and 50 μM of tested compound represented as mean percent ± SD.
