# Supplementary material for: A Fine-Tuned Lipophilicity/Hydrophilicity Ratio Governs Antibacterial Potency and Selectivity of Bifurcated Halogen Bond-Forming NBTIs
Source: Antibiotics (Basel). 2021 Jul 15;10(7):862. doi: 10.3390/antibiotics10070862 (PMC8300687; doi:10.3390/antibiotics10070862)
Supplement: Supplementary file 1 [file antibiotics-10-00862-s001.zip › antibiotics-1265711-supplementary.pdf]

Supporting Information

A Fine-tuned Lipophilicity/Hydrophilicity Ratio Governs Antibacterial Potency and Selectivity of Bifurcated Halogen Bond-Forming NBTIs

Anja Kolarič, Maja Kokot, Martina Hrast, Matjaž Weiss, Irena Zdovc, Jurij Trontelj, Simon Žakelj, Marko Anderluh,* and Nikola Minovski*

Table S1. The HPLC method parameters, equilibrium solubility at pH 6.8 and PAMPA permeability of our NBTIs and reference compounds.

Cmpd	HPLC method parameters			Equlibrium solubility at pH 6.8		PAMPA permeability		clogP[a]	logD[b]
	%B	*Λ$_{max}$* [nm]	*RT* [min]	[mg/L]	max. dose for "high" solubility in mg	[nm/s]	classification [low/high]		
**3**	11	324	3.0	349	174	54	high	3.02	1.45
**4**	17	250	2.8	585	293	142	high	3.86	1.9
**5**	16	212	3.3	381	191	255	high	3.63	1.75
**6**	5	216	2.6	797	399	56	high	3.56	0.75
**7**	18	240	2.5	883	441	26	intermediate	2.76	0.89
**8**	15	212	4.0	106	53	106	high	3.68	1.25
**9**	10	214	2.8	7551	3776	33	high	2.53	0.84
**10**	7	216	3.2	5696	2848	41	high	2.47	0.79
**11**	8	212	3.3	1369	684	109	high	3.58	1.17
**12**	10	212	2.7	460	230	121	high	3.72	1.47
**13**	15	216	2.9	119	59	132	high	4.29	1.89
**14**	16	216	2.9	72	36	315	high	4.44	2.02
**15**	22	214	2.7	184	92	398	high	4.70	2.18
**16**	6	214	2.3	3387	1694	15	intermediate	2.54	0.44
**17**	6	216	2,1	560	280	3.1	low	2.09	0.19
**18**	12	214	3.1	537	268	167	high	3.74	0.18
**19**	16	216	2.7	79	39	90	high	3.68	2.00
**20**	11	214	3.2	103	52	30	high	3.21	2.07
**21**	13	214	2.11	209	104	83	high	3.11	1.63
**22**	9	216	2,1	1460	730	82	high	2.73	0.76
**Metoprolol**	18	222	2.6	30394	15197	42	high	/	/
**Verapamil**	34	230	2.9	2684	1342	462	high	/	/
**Theophyllin**	5	274	3.0	8218	4109	27	high	/	/
**Digoxin**	31	222	3.0	52	26	5.8	intermediate	/	/
**Atenolol**	5	224	2.8	21324	10662	0.31	low	/	/
**Furosemide**	29	274	2.8	3752	1876	0.18	low	/	/

[a]clogP was calculated with ChemDraw 15.0.0.106. [b]logD was calculated at pH = 7.4 with MarvinSketch 20.17.

Table S2. Comparison of *S. aureus* DNA gyrase IC_{50} and the lengths of RHS fragments for our previously published compound 13 and known NBTIs.

Compd	13[1]	GSK299423[2]	AM8191[3]	ACT387042[4]
Structure	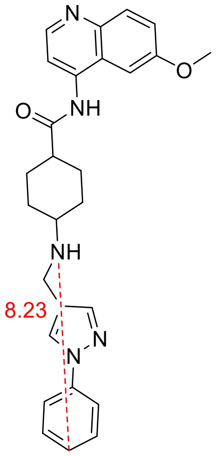	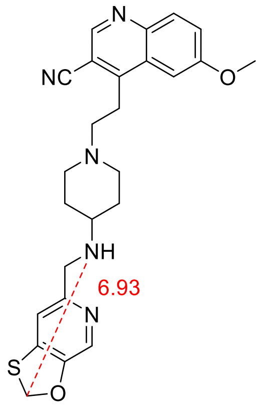	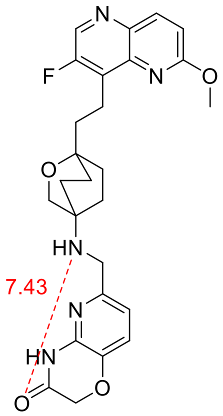	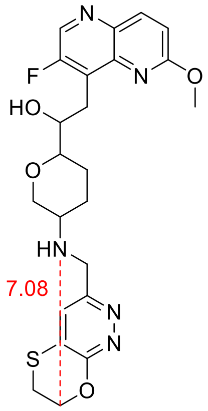
***S. aureus* DNA gyrase IC_{50} (μM)**	0.83	0.014	0.22	0.125

Atom distances depicted in red dots are in Å.

Table S3. Selected templates for homology modelling.

Compd	*E. coli*	*S. aureus*
**ParC (protein ID from Uniprot)**	P0AFI2	Q2FYS4
**ParE (protein ID from Uniprot)**	P20083	Q2FYS5
**Template protein (PDB ID)**	3ksa	3raf
**GMQE**	0.41	0.45
**Identity**	43.0	68.3
**Resolution**	3.3	3.2

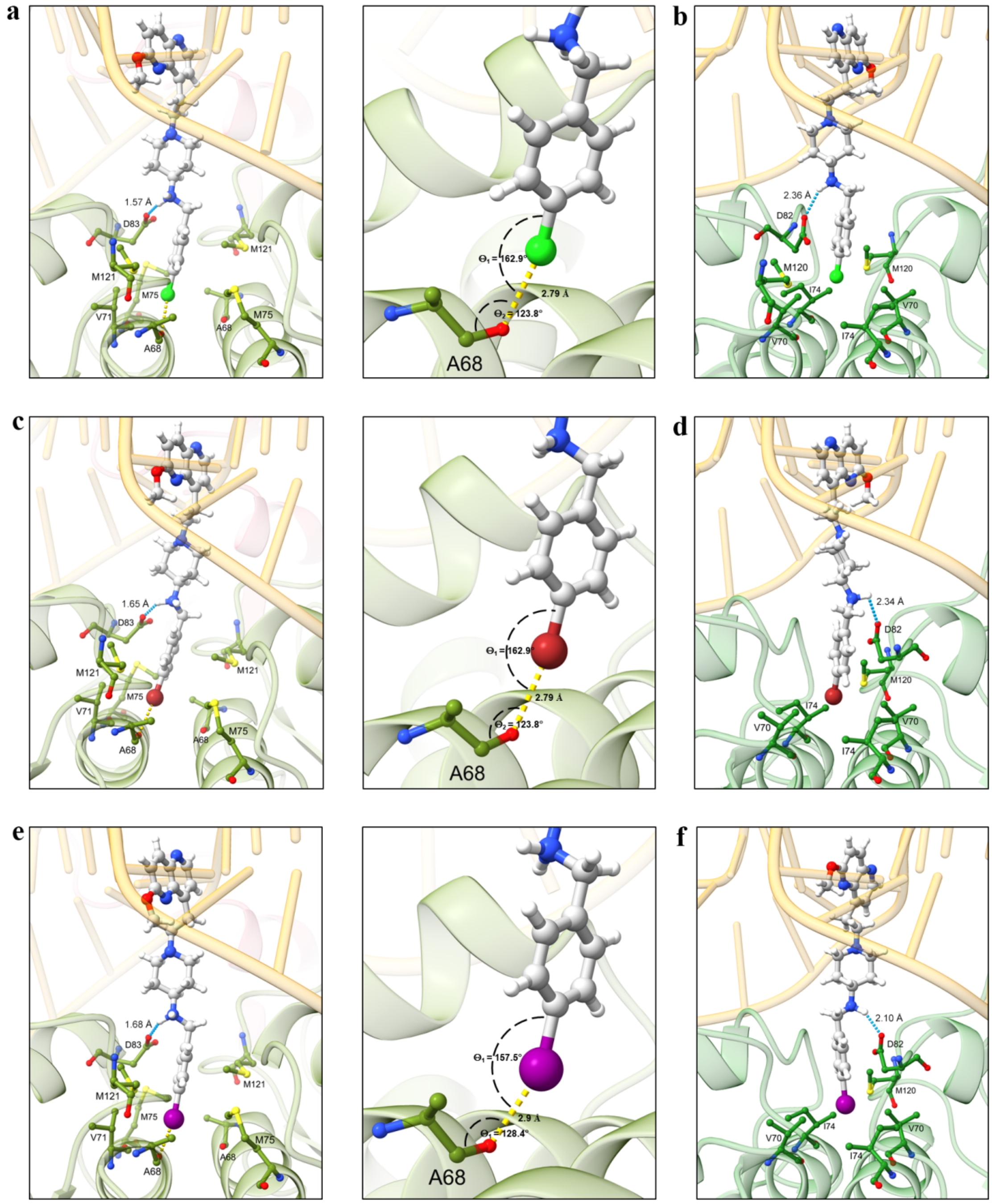

Figure S1. Predicted binding modes of compound **13**-**15** within the *S. aureus* and *E. coli* DNA gyrase NBTIs binding site (PDB ID: 2XCS[2] and our constructed homology model, respectively), revealing key intermolecular interactions and a close view of predicted halogen bonding. **a)** compound **13** within *S. aureus* DNA gyrase **b)** compound **13** within *E. coli* DNA gyrase **c)** compound **14** within *S. aureus* DNA gyrase **d)** compound **14** within *E. coli* DNA gyrase **e)** compound **15** within *S. aureus* DNA gyrase **f)** compound **15** within *E. coli* DNA gyrase. *S. aureus* and *E. coli* DNA gyrase are shown in dark and light green ribbons, respectively, compounds and amino acids are coloured by element (ball and sticks representation), while DNA in pale orange. Ionic interactions are represented as cyan dots and halogen-bonding interactions as yellow dots.

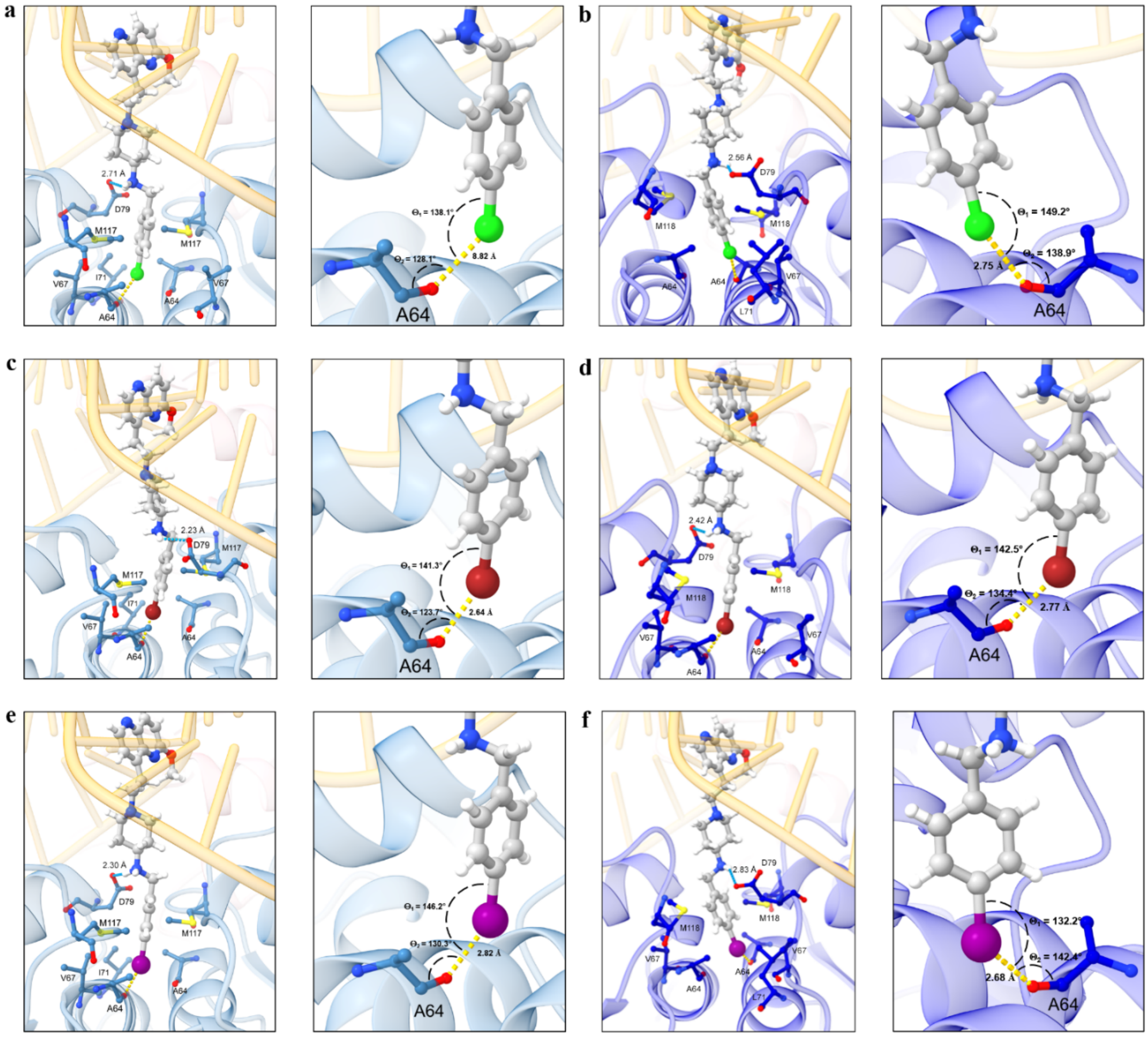

Figure S2. Predicted binding modes of compound **13**-**15** within the *S. aureus* and *E. coli* Topo IV NBTIs binding site (our constructed homology models), revealing key intermolecular interactions and a close view of predicted halogen bonding. **a)** compound **13** within *S. aureus* Topo IV **b)** compound **13** within *E. coli* Topo IV **c)** compound **14** within *S. aureus* Topo IV **d)** compound **14** within *E. coli* Topo IV **e)** compound **15** within *S. aureus* Topo IV **f)** compound **15** within *E. coli* Topo IV. *S. aureus* and *E. coli* Topo IV are shown in light and dark blue ribbons, respectively, compounds and amino acid residues are colored by element (ball and sticks representation), while DNA in pale orange. Ionic interactions are represented as cyan dots and halogen-bonding interactions as yellow dots.

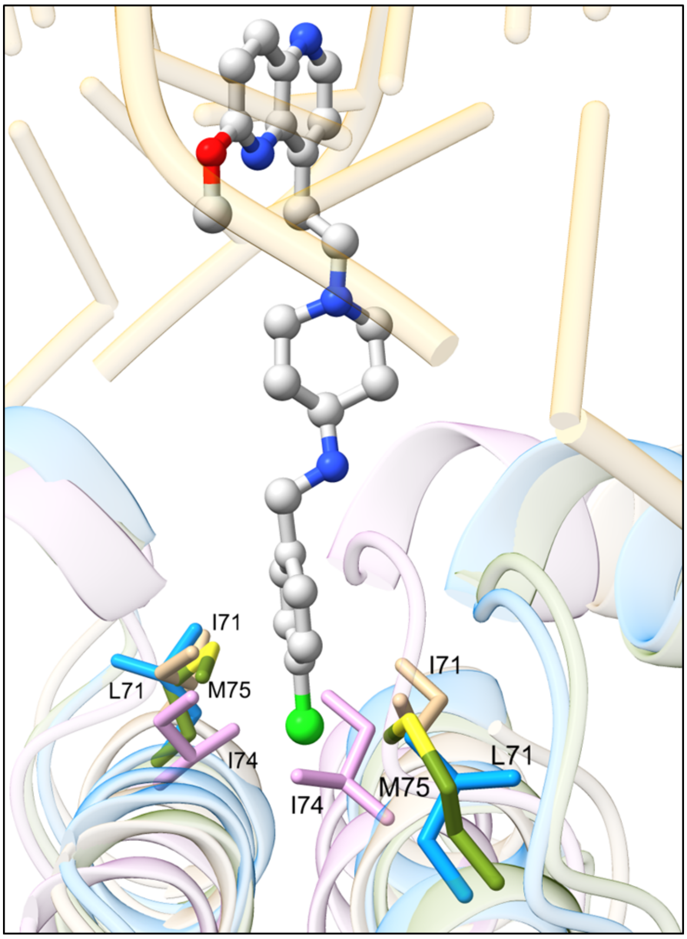

Figure S3. Met75 from *S. aureus* DNA gyrase (green, PDB code 6Z1A)(Kolarič et al., 2021) in comparison to Ile74 in *E. coli* DNA gyrase (violet, PDB code 4CKK),[5] Ile71 in *S. aureus* Topo IV (beige, PDB code 2INR), and Leu71 in *E. coli* Topo IV homology model utilizing *Klebsiella pneumoniae* Topo IV structure as a template (blue, PDB code 5EIX).[6] For clarity, the corresponding GyrA and ParC subunits originating from *S. aureus* and *E. coli*, respectively, were used for the structural superimposition. Compound **13** (inset, silver)[7] was inserted for observation of steric hindrance. Enzymes are shown in ribbons, compound **13** (ball and sticks representation) and amino acids (sticks representation) are colored by element, while DNA in pale orange.

Scheme S1. Synthesis of 2-phenyl-2*H*-1,2,3-triazole-4-carbaldehyde (23).

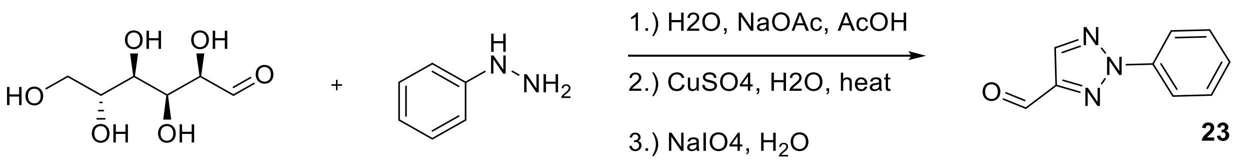

23

NMR spectra

1-(2-(6-Methoxy-1,5-naphthyridin-4-yl)ethyl)-*N*-((1-methyl-1*H*-benzo[d]imidazol-2-yl)methyl)piperidin-4-amine (**3**)

^{1}H NMR (400 MHz, $CDCl_3$)

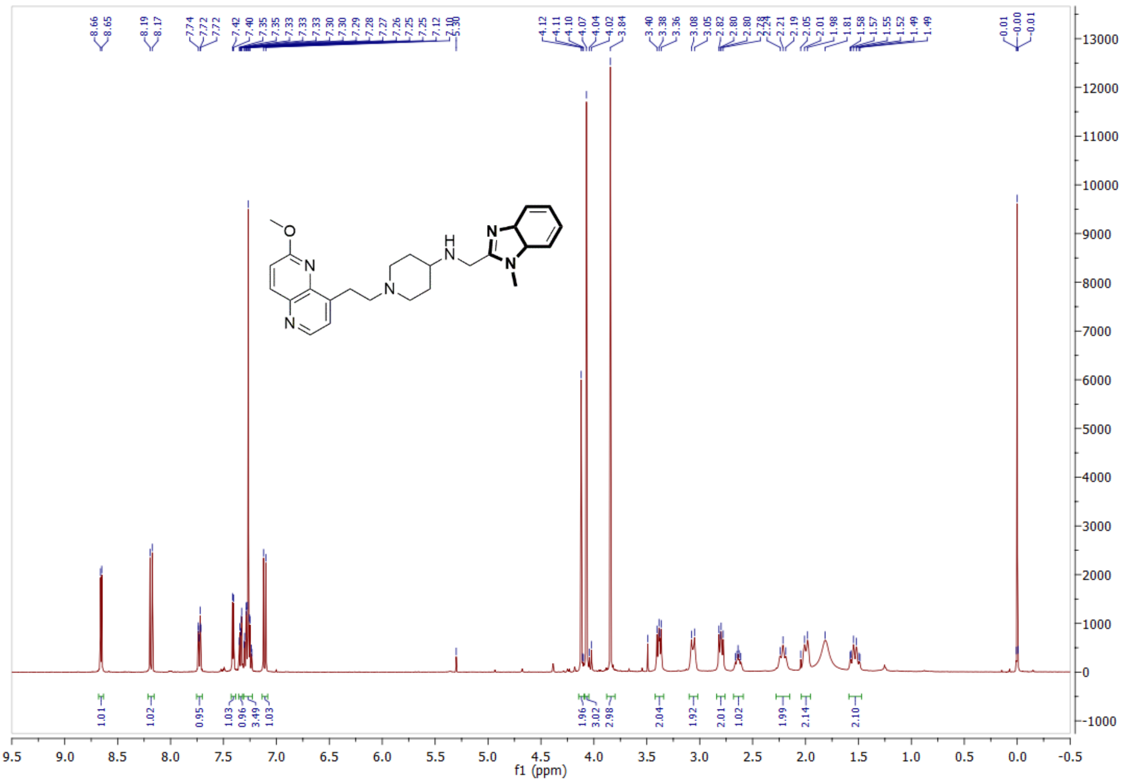

^{13}C NMR (100 MHz, $CDCl_3$)

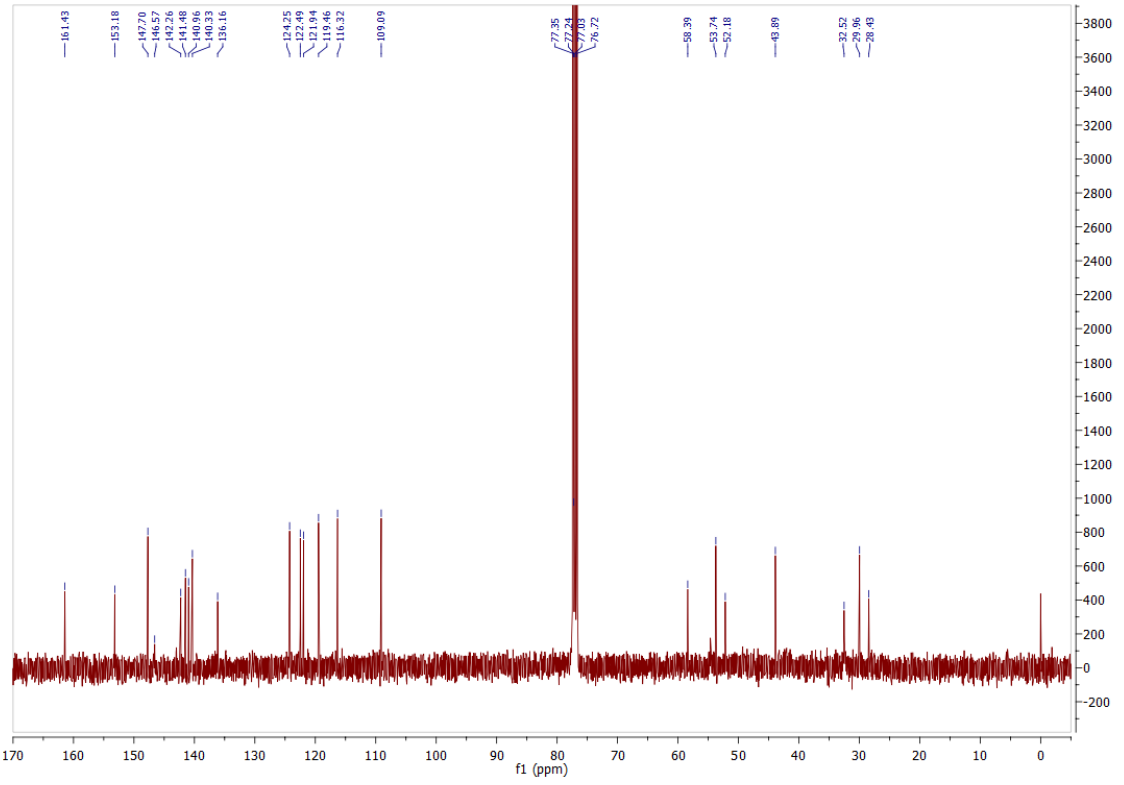

N-((1-(4-Fluorophenyl)-1*H*-pyrazol-4-yl)methyl)-1-(2-(6-methoxy-1,5-naphthyridin-4-yl)ethyl)piperidin-4-amine (**4**)

^{1}H NMR (400 MHz, $CDCl_3$)

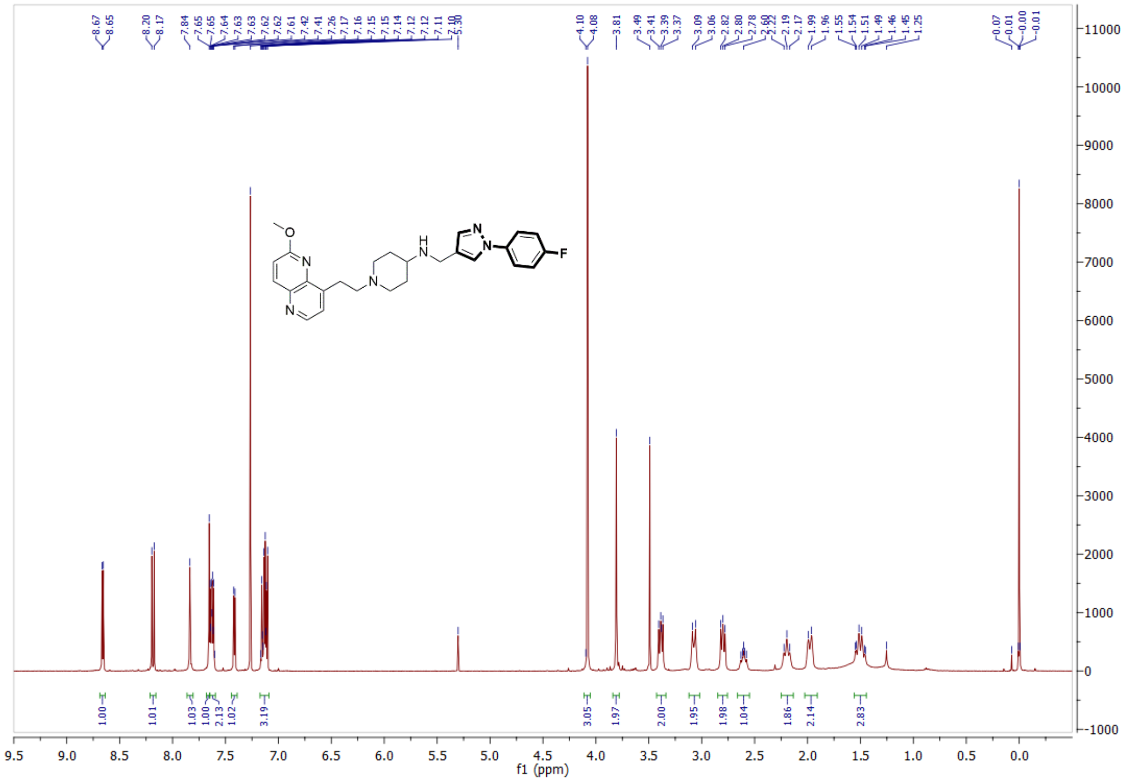

^{13}C NMR (100 MHz, $CDCl_3$)

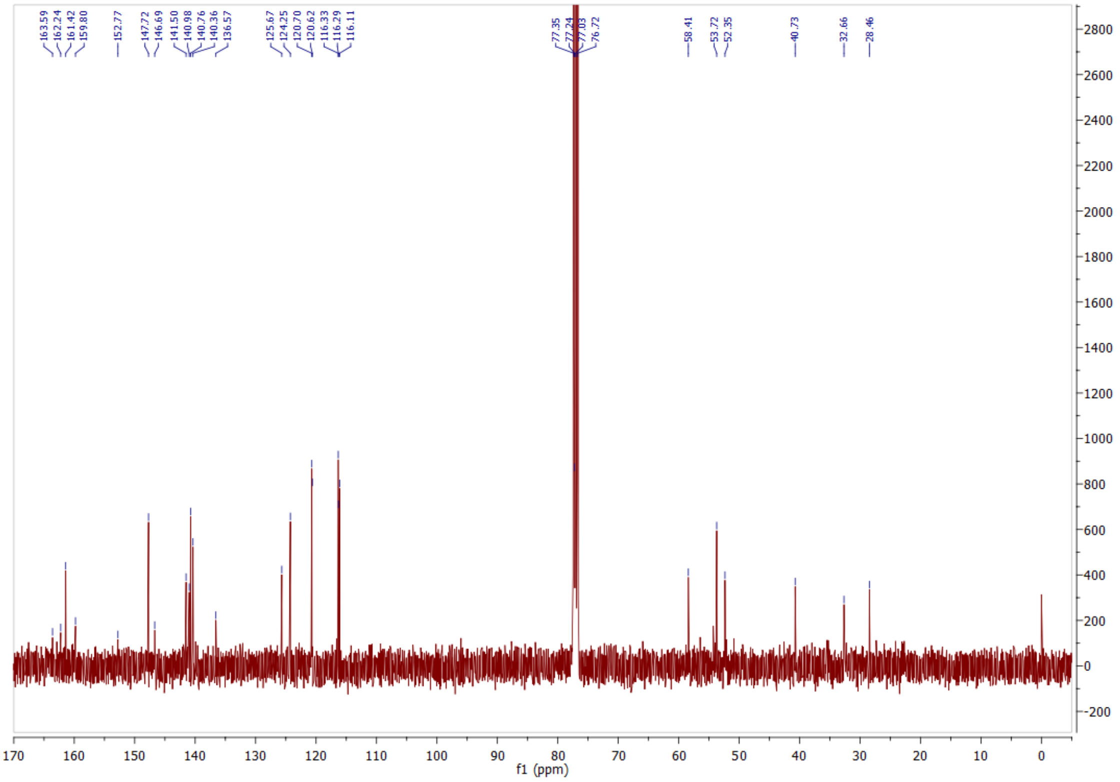

1-(2-(6-Methoxy-1,5-naphthyridin-4-yl)ethyl)-*N*-((1-phenyl-1*H*-pyrazol-4-yl)methyl)piperidin-4-amine (**5**)

^{1}H NMR (400 MHz, $CDCl_3$)

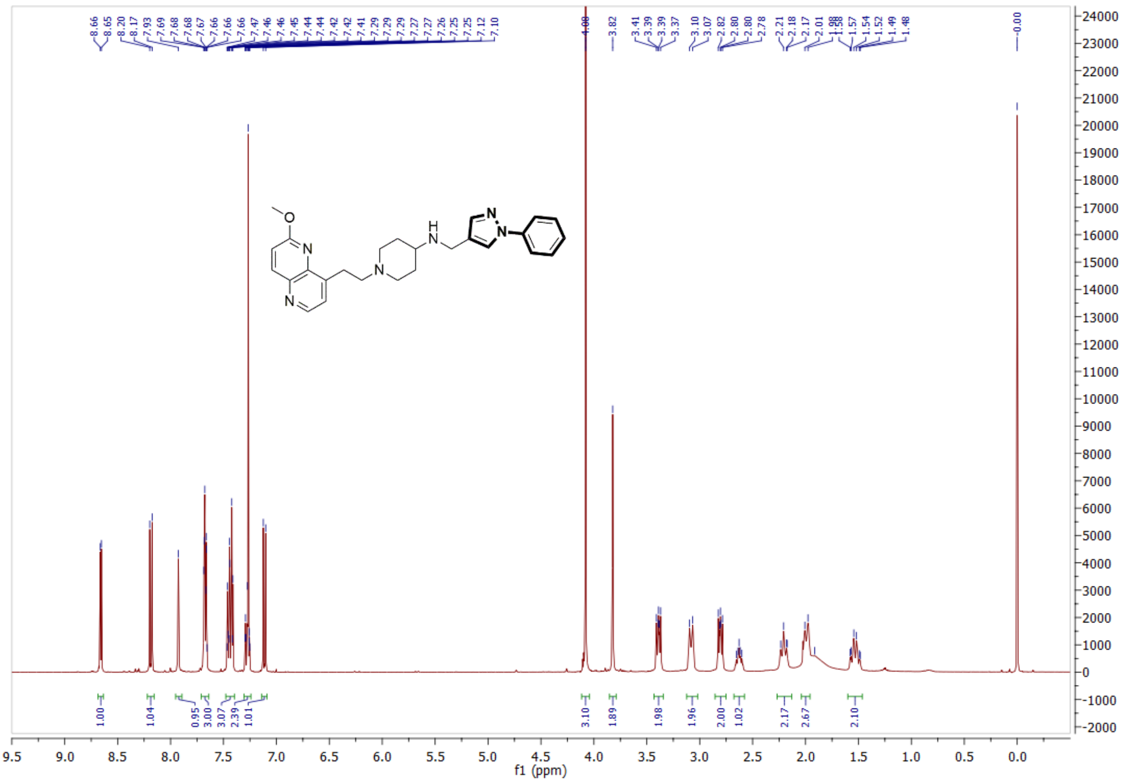

^{13}C NMR (100 MHz, $CDCl_3$)

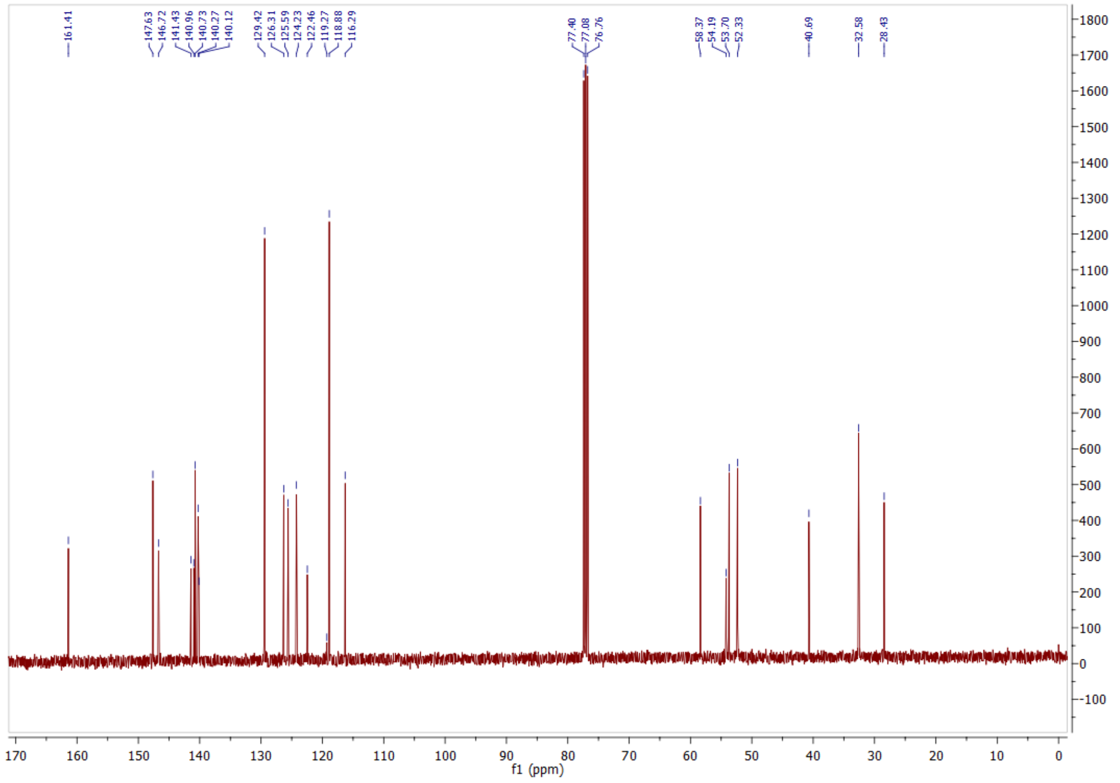

N-((2,8-dimethylimidazo[1,2-*a*]pyridin-3-yl)methyl)-1-(2-(6-methoxy-1,5-naphthyridin-4-yl)ethyl)piperidin-4-amine (**6**)

^{1}H NMR (400 MHz, $CDCl_3$)

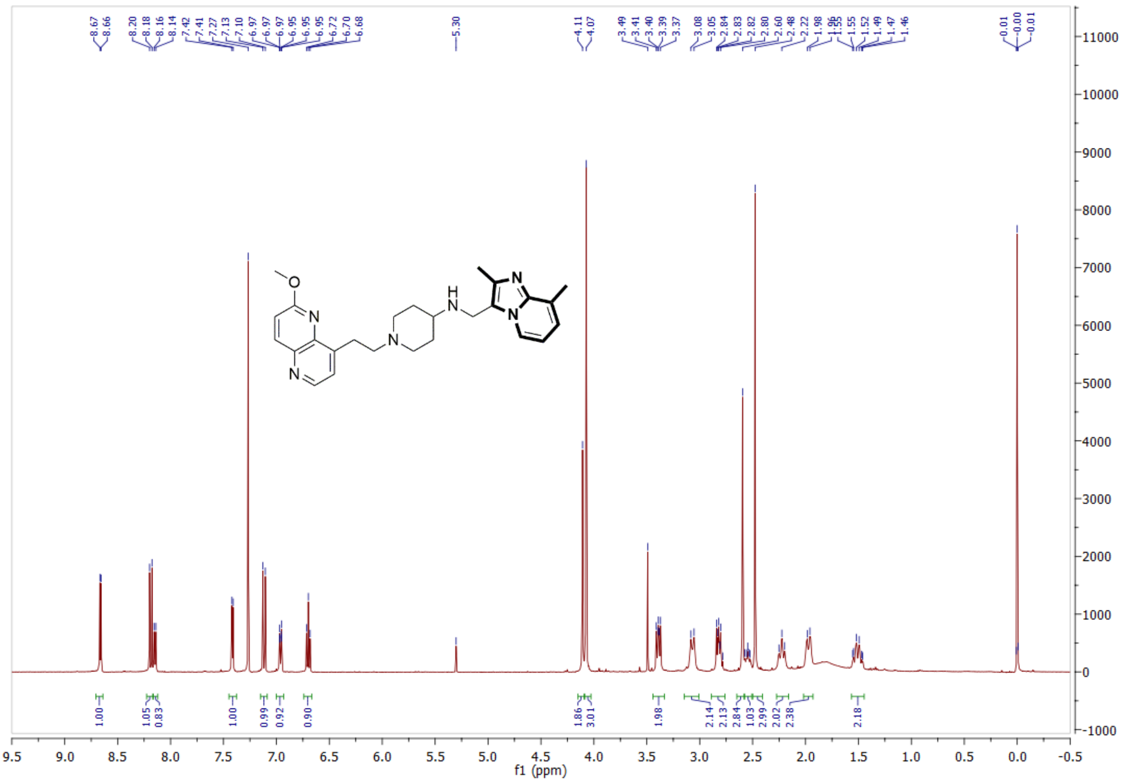

^{13}C NMR (100 MHz, $CDCl_3$)

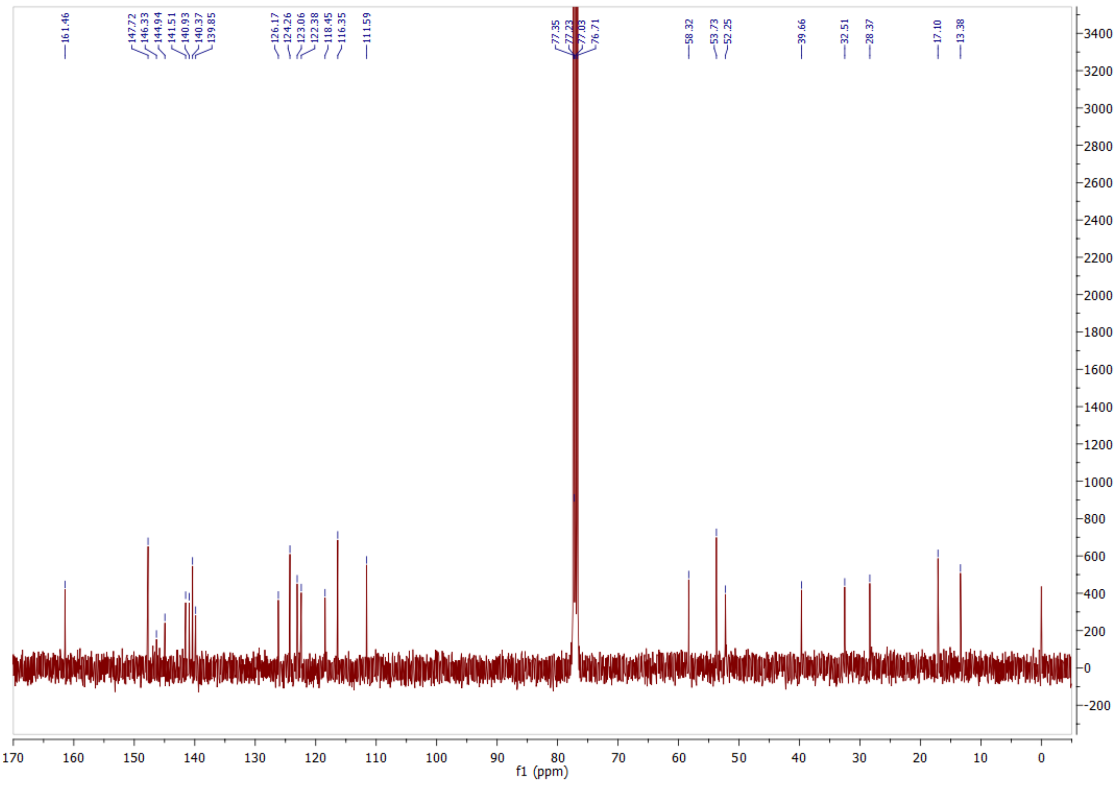

8-Cyano-*N*-(1-(2-(6-methoxy-1,5-naphthyridin-4-yl)ethyl)piperidin-4-yl)imidazo[1,2-a]pyridine-2-carboxamide (**7**)

^{1}H NMR (400 MHz, $CDCl_3$)

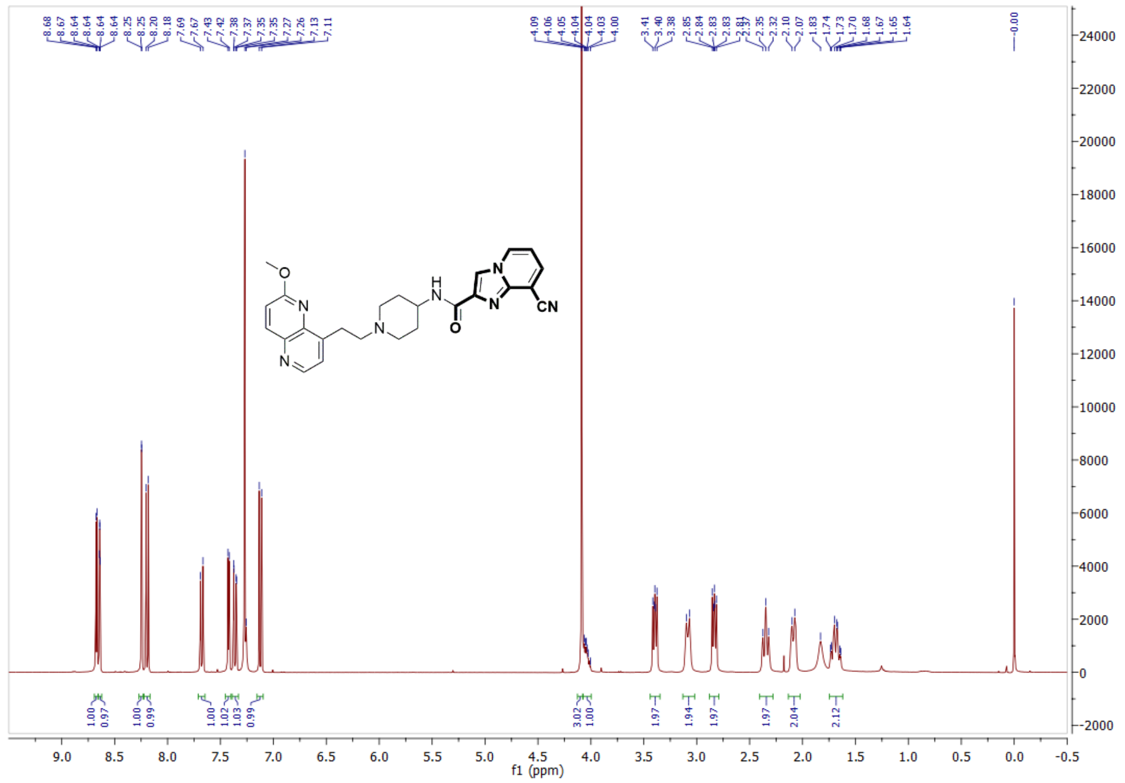

^{13}C NMR (100 MHz, $CDCl_3$)

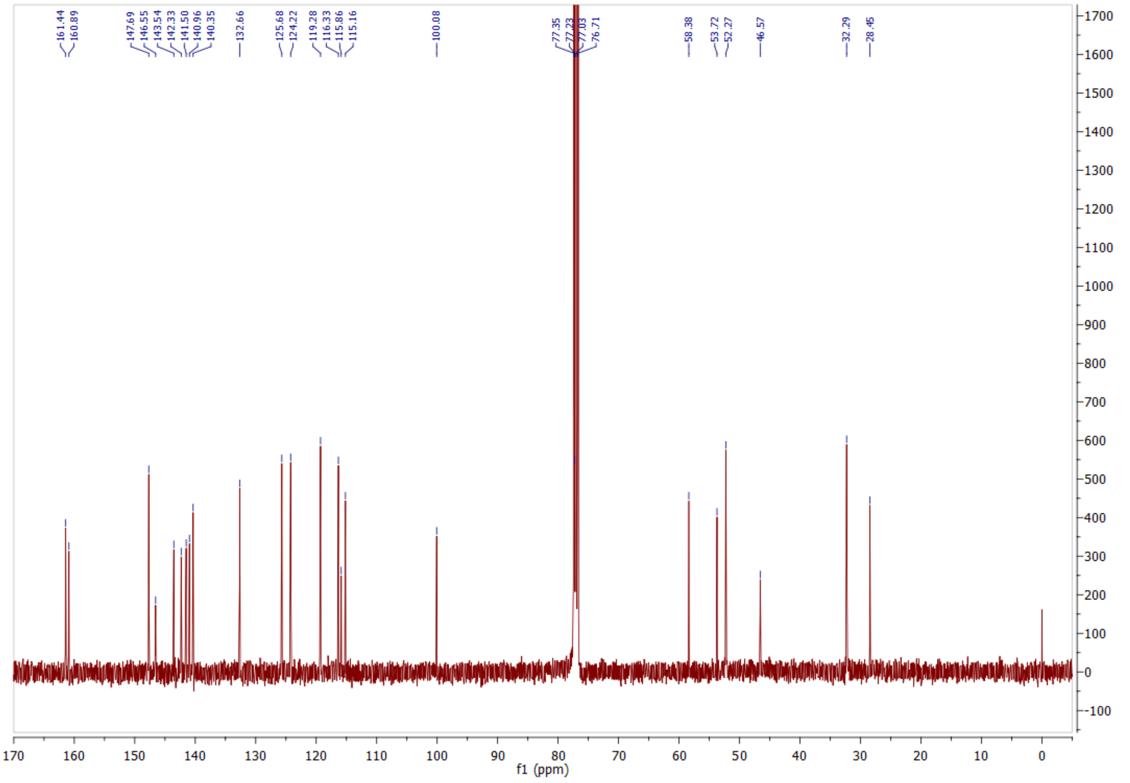

1-(2-(6-methoxy-1,5-naphthyridin-4-yl)ethyl)-*N*-((2-phenyl-2*H*-1,2,3-triazol-4-yl)methyl)piperidin-4-amine (**8**)

^{1}H NMR (400 MHz, $CDCl_3$)

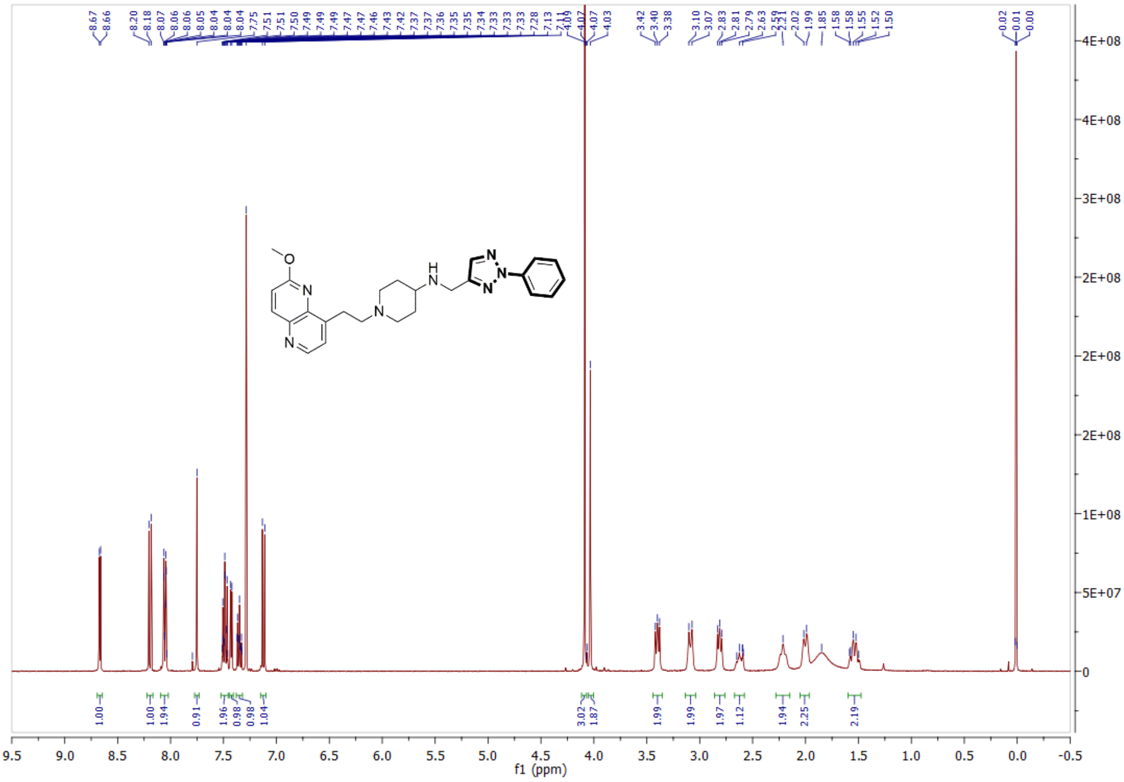

^{13}C NMR (100 MHz, $CDCl_3$)

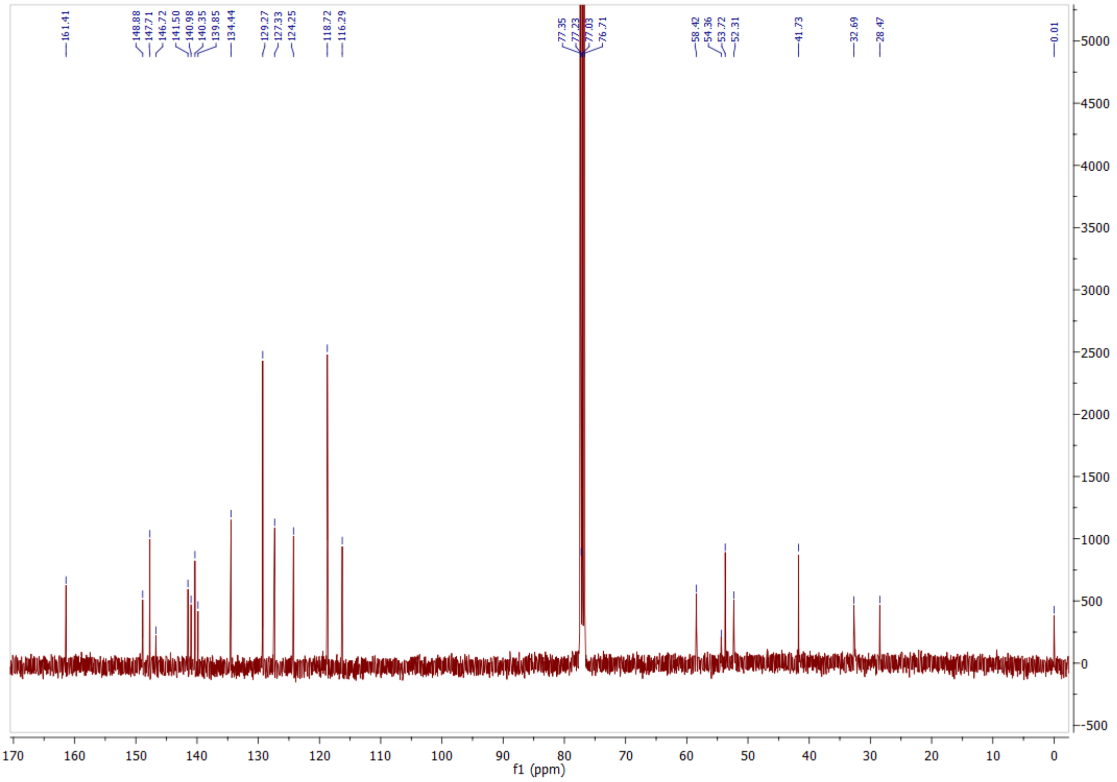

N-((1-isopropyl-1*H*-pyrazol-4-yl)methyl)-1-(2-(6-methoxy-1,5-naphthyridin-4-yl)ethyl)piperidin-4-amine (**9**)

^{1}H NMR (400 MHz, $CDCl_3$)

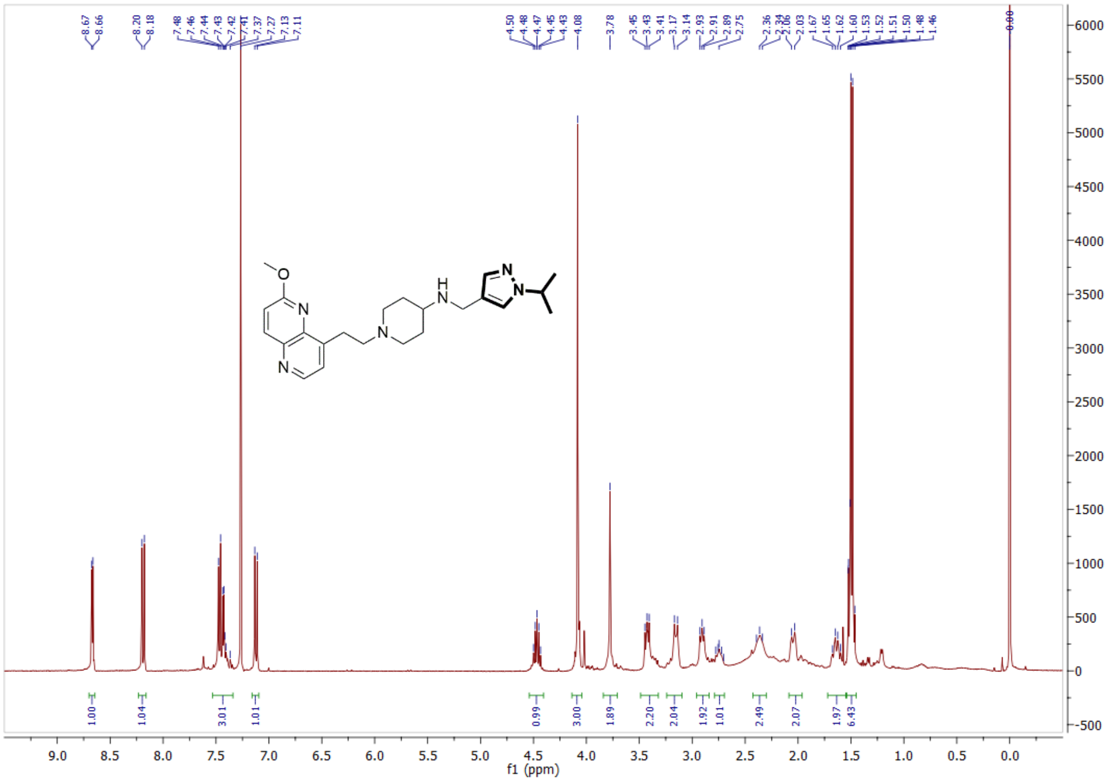

^{13}C NMR (100 MHz, $CDCl_3$)

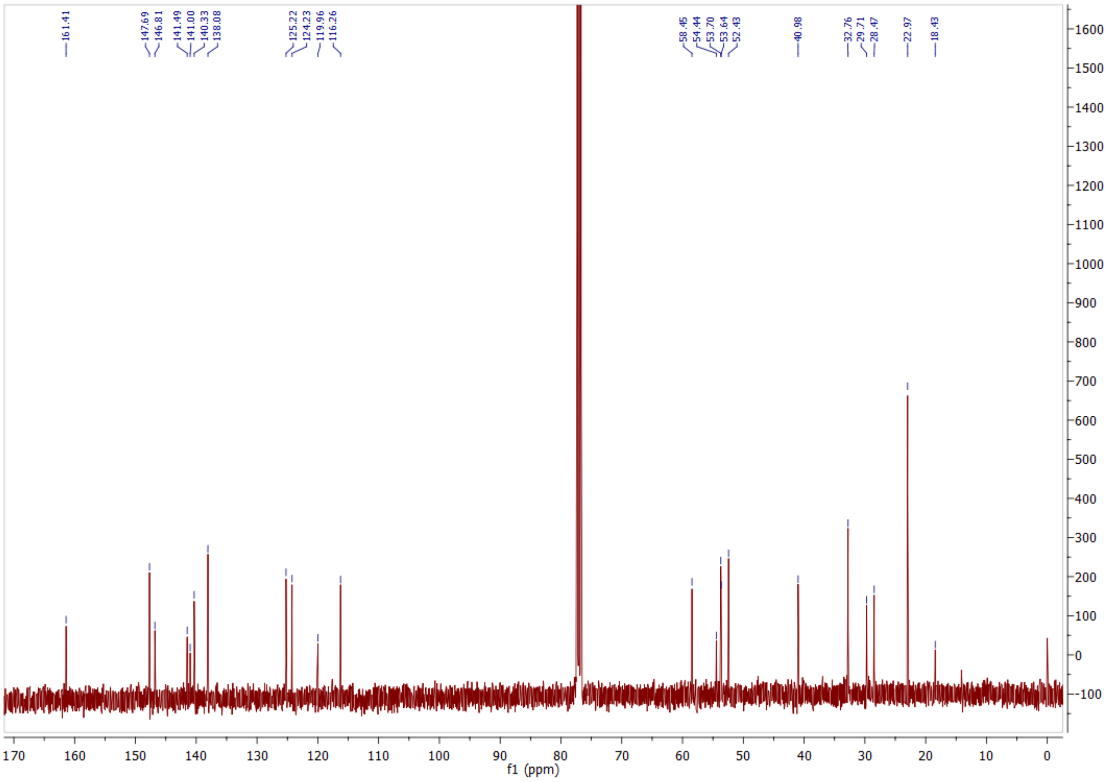

N-((1-allyl-1*H*-pyrazol-4-yl)methyl)-1-(2-(6-methoxy-1,5-naphthyridin-4-yl)ethyl)piperidin-4-amine (**10**)

^{1}H NMR (400 MHz, $CDCl_3$)

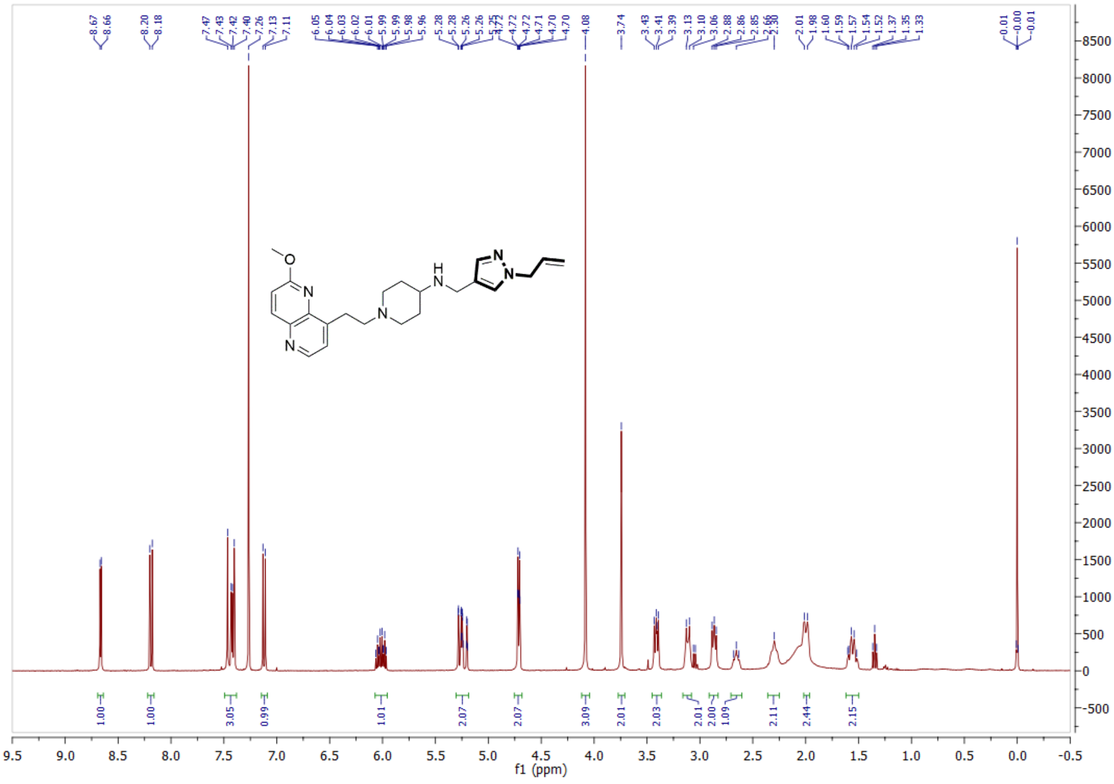

^{13}C NMR (100 MHz, $CDCl_3$)

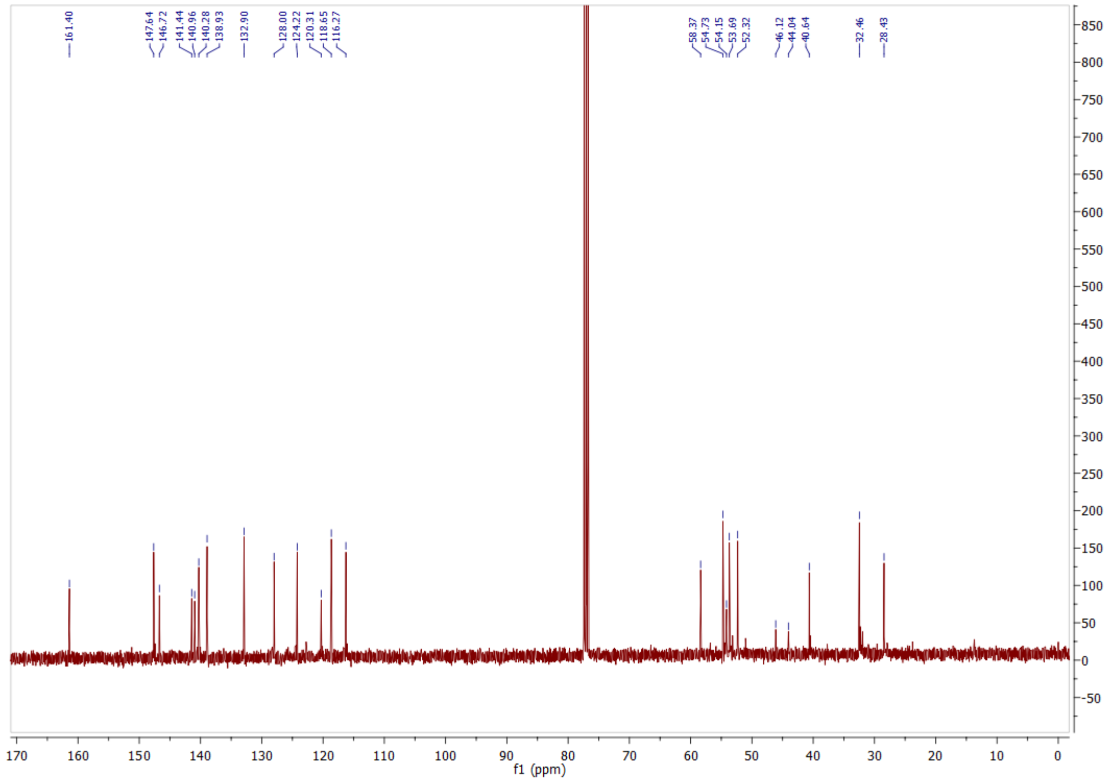

N-(4-(difluoromethoxy)-3-methoxybenzyl)-1-(2-(6-methoxy-1,5-naphthyridin-4-yl)ethyl)piperidin-4-amine (**19**)

^{1}H NMR (400 MHz, $CDCl_3$)

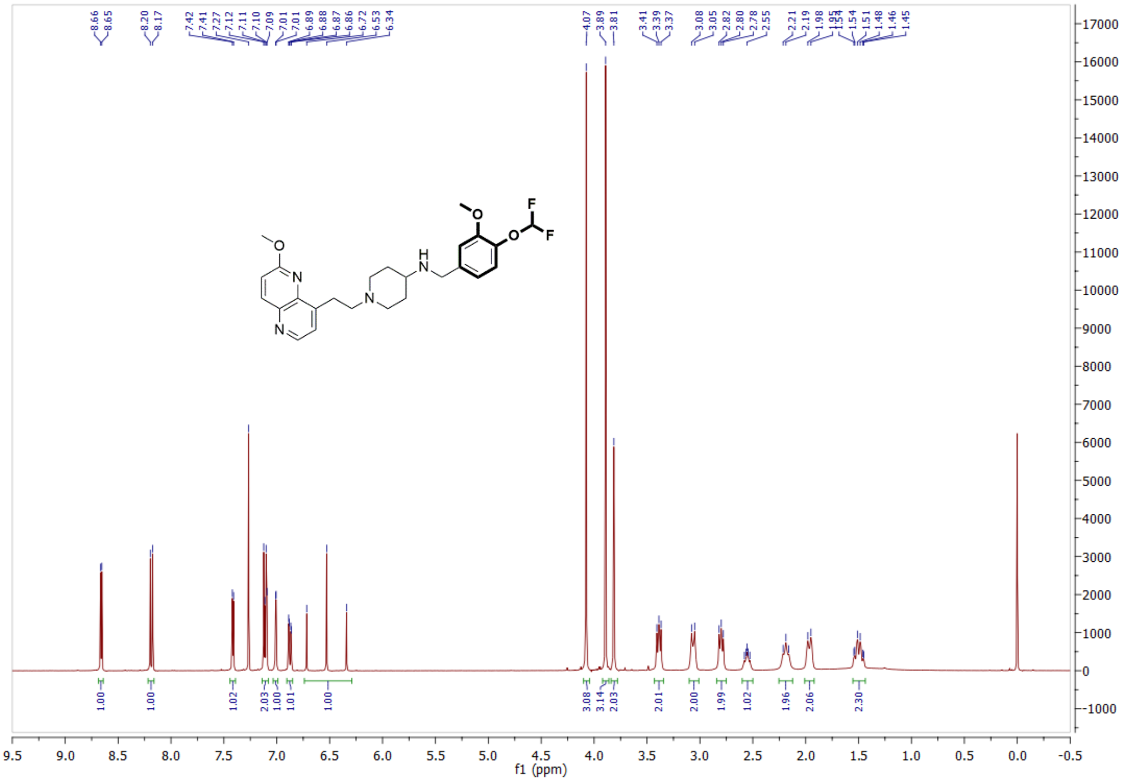

^{13}C NMR (100 MHz, $CDCl_3$)

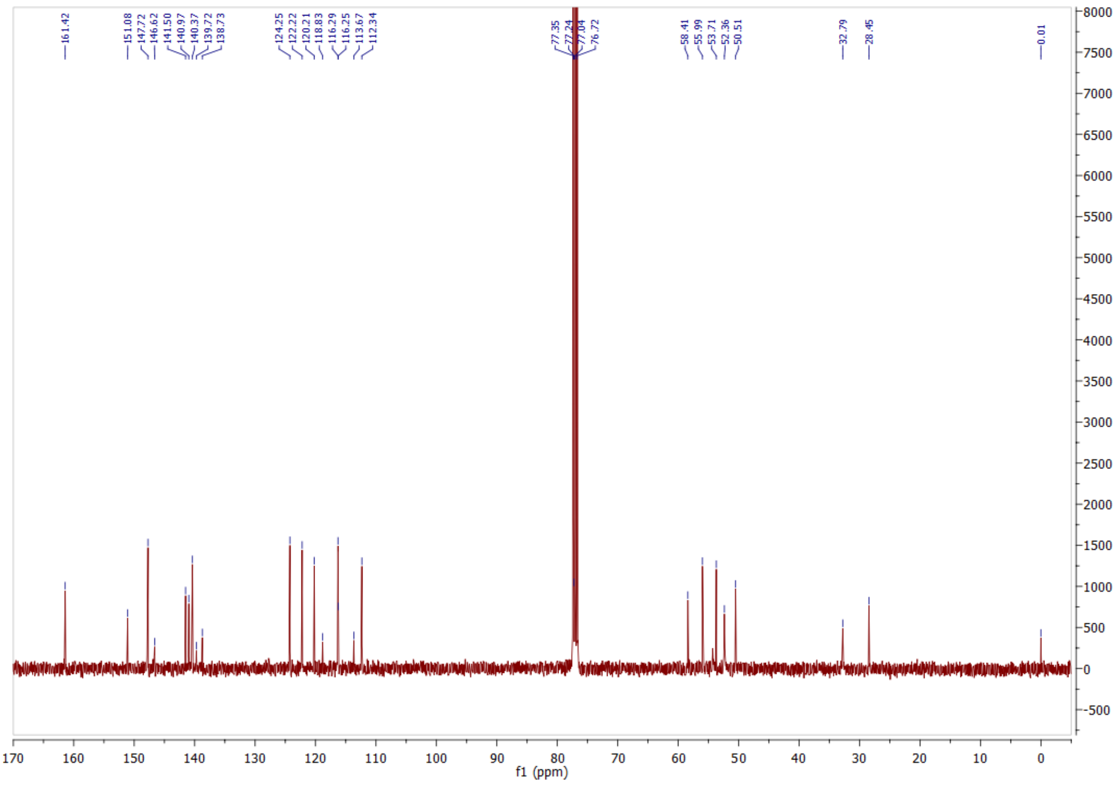

2-(difluoromethoxy)-5-((1-(2-(6-methoxy-1,5-naphthyridin-4-yl)ethyl)piperidin-4-ylamino)methyl)phenol (**20**)

^{1}H NMR (400 MHz, $CDCl_3$)

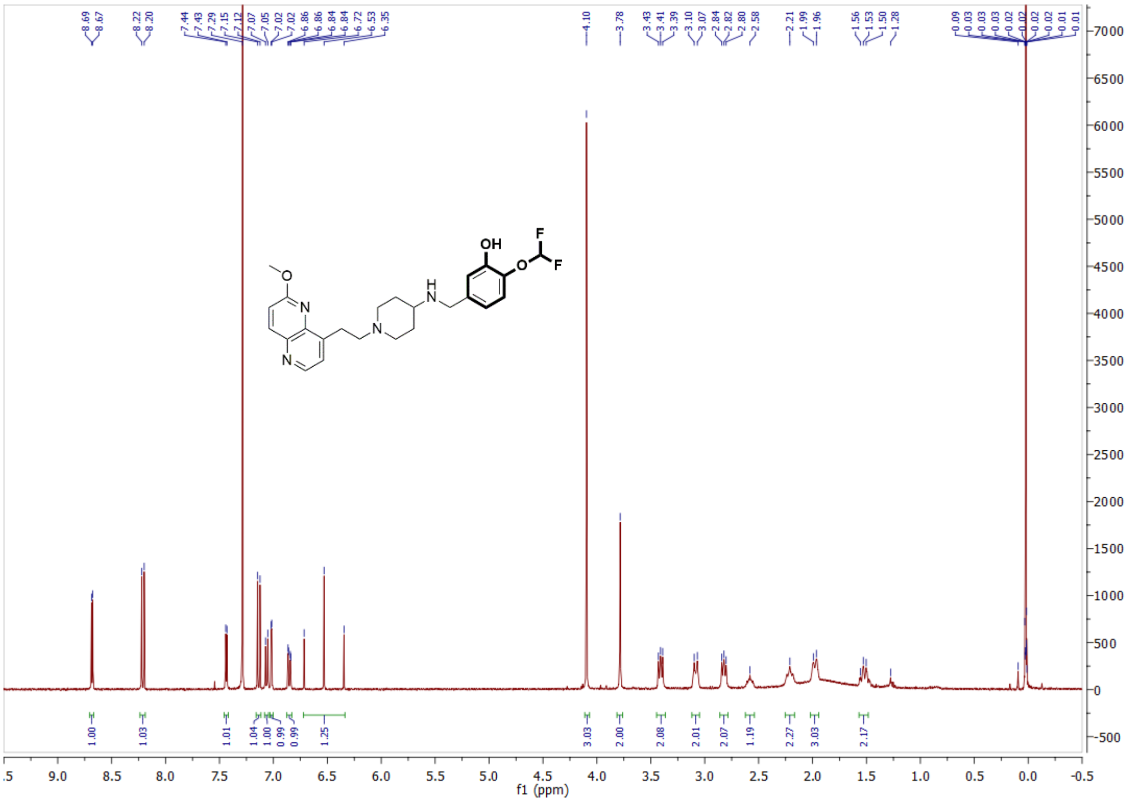

^{13}C NMR (100 MHz, $CDCl_3$)

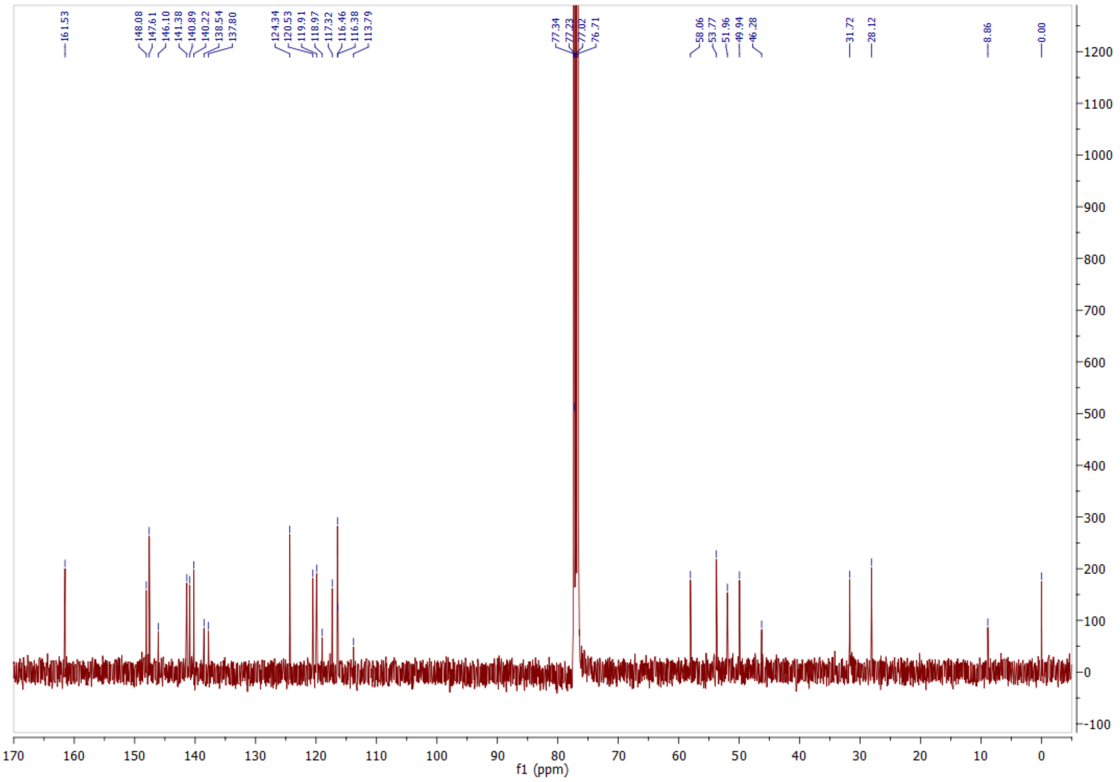

1-(2-(6-methoxy-1,5-naphthyridin-4-yl)ethyl)-N-((6-(trifluoromethyl)pyridin-3-yl)methyl)piperidin-4-amine (**21**)

^{1}H NMR (400 MHz, $CDCl_3$)

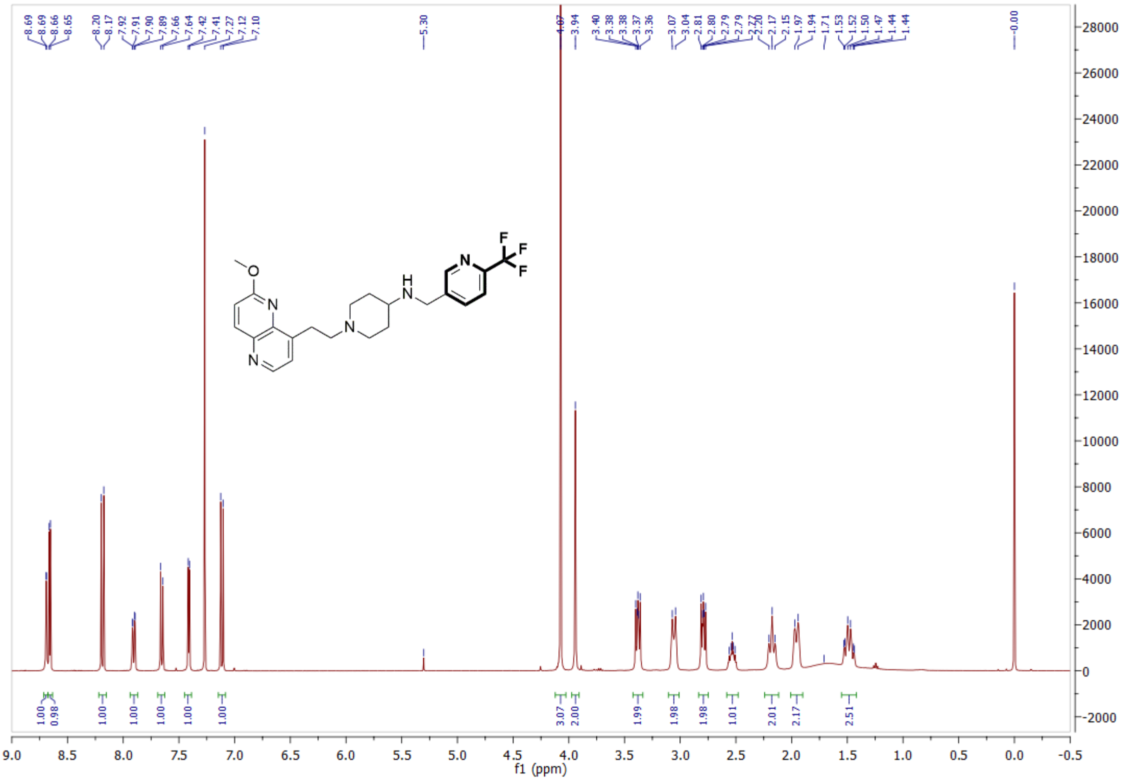

^{13}C NMR (100 MHz, $CDCl_3$)

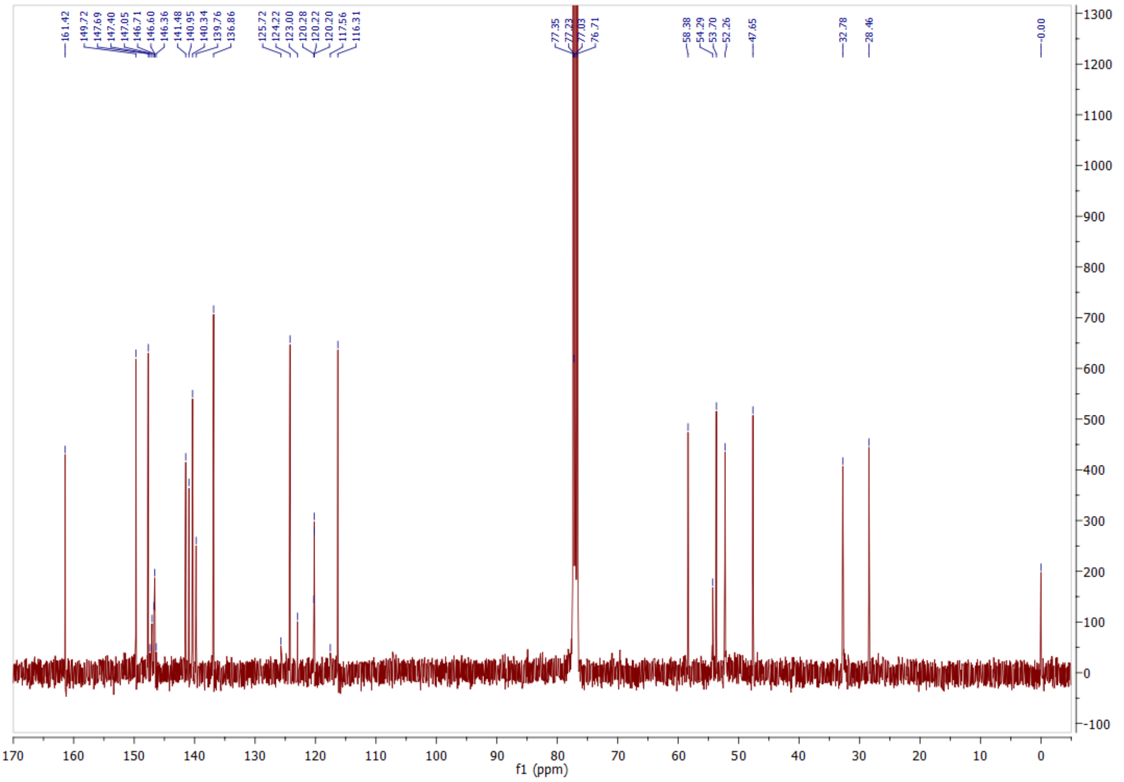

1-(2-(6-methoxy-1,5-naphthyridin-4-yl)ethyl)-*N*-((6-methoxypyridin-3-yl)methyl)piperidin-4-amine (**22**)

^{1}H NMR (400 MHz, $CDCl_3$)

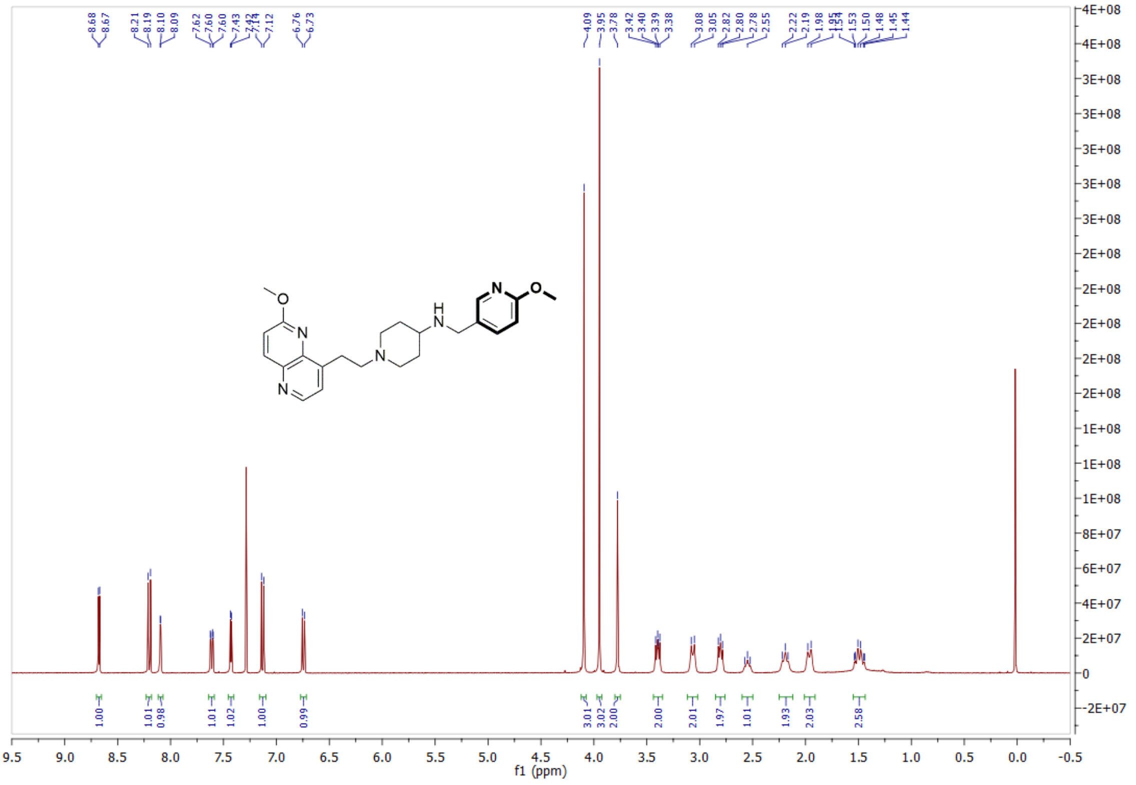

^{13}C NMR (100 MHz, $CDCl_3$)

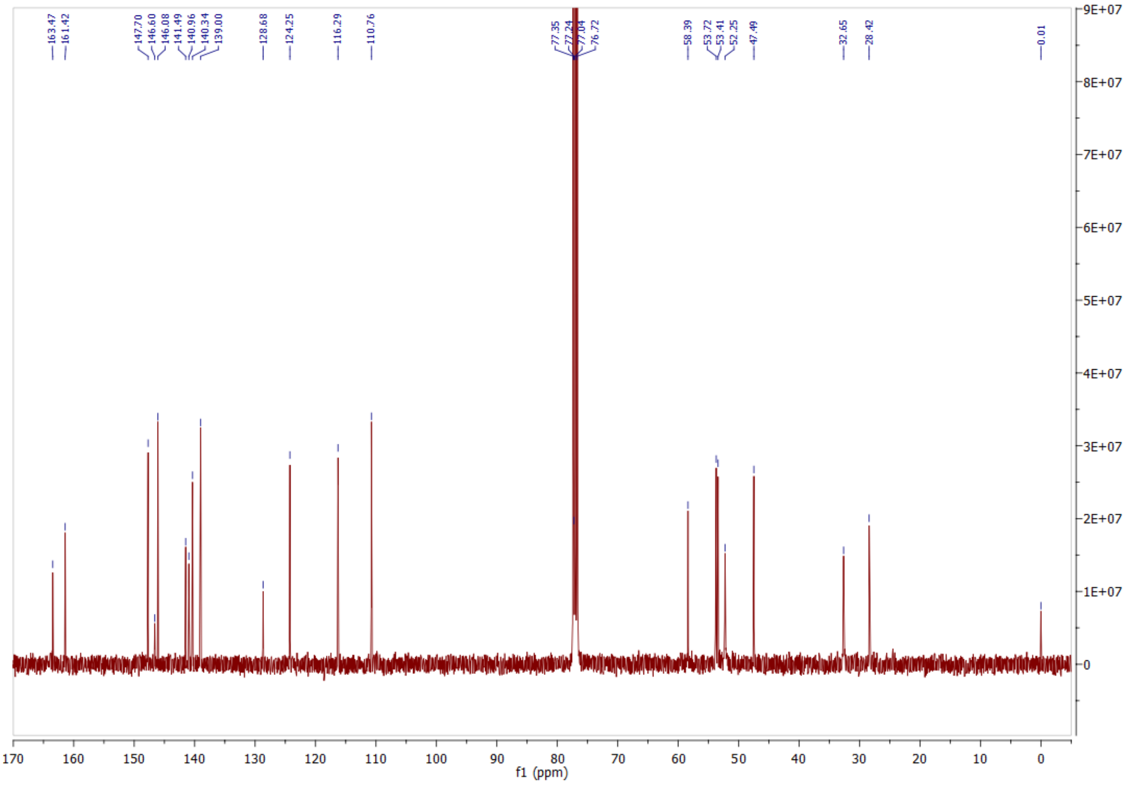

Table S4. Minimal inhibitory concentration (MIC) in molar.

Compd	*S. aureus* MIC [μM][a]	*E. coli* MIC [μM][b]	*E. coli* N43 MIC [μM][c]
**3**	2.32	297.29	37.16
**4**	4.34	69.48	4.34
**5**	0.57	36.15	2.26
**6**	287.91	287.91	287.91
**7**	280.99	280.99	280.99
**8**	1.13	144.29	2.26
**9**	0.31	19.58	0.61
**10**	4.92	39.36	2.46
**11**	10.62	169.99	21.25
**12**	2.54	81.12	10.14
**13**	0.30	9.73	1.22
**14**	0.07	4.39	0.27
**15**	0.01	3.98	0.16
**16**	9.84	314.87	39.36
**17**	4.77	305.11	38.14
**18**	0.31	78.53	2.45
**19**	2.12	67.72	2.12
**20**	1.09	17.45	1.09
**21**	0.56	71.83	2.25
**22**	1.19	38.14	2.38

[a]*S. aureus* ATCC 29213. [b]*E. coli* ATCC 25922. [c]*E. coli* CGSC# 5583; AcrA knockout strain (knockout of cell membrane efflux pump).

Table S5. The metabolic activity of cells at 1 μM and 50 μM of tested compound represented as mean percent ± SD.

Compd	HUVEC MA (%)		HepG2 MA (%)	
	**1 μM**	**50 μM**	**1 μM**	**50 μM**
**3**	114.2 ± 10.3	87.6 ± 10.0	108.7 ± 10.7	28.4 ± 5.6
**4**	112.3 ± 6.7	0.3 ± 0.2	112.2 ± 11.6	0.3 ± 1.3
**5**	107.5 ± 4.4	0.2 ± 0.3	112.4 ± 7.5	0.4 ± 1.7
**6**	90.5 ± 20.8	56.4 ± 3.1	102.9 ± 3.8	0.4 ± 0.8

**7**	93.7 ± 12.9	36.5 ± 2.6	111.0 ± 10.1	0.8 ± 1.2
**8**	98.5 ± 7.7	35.0 ± 7.2	110.0 ± 10.6	0.7 ± 0.3
**9**	92.0 ± 1.7	86.9 ± 3.9	104.8 ± 1.8	68.5 ± 7.0
**10**	88.6 ± 21.2	84.2 ± 19.5	108.5 ± 6.3	77.0 ± 2.4
**11**	92.9 ± 16.6	63.4 ± 11.3	107.1 ± 7.2	1.4 ± 0.3
**12**	105.2 ± 15.1	75.6 ± 12.0	98.0 ± 3.9	-1.3 ± 2.2
**13**	86.1 ± 17.1	-0.6 ± 0.4	84.5 ± 11.1	0.9 ± 0.1
**14**	92.0 ± 4.5	-0.5 ± 0.2	84.3 ± 8.9	-0.1 ± 0.3
**15**	108.0 ± 6.6	-0.3 ± 0.5	102.6 ± 2.3	0.5 ± 0.3
**16**	97.7 ± 9.4	63.1 ± 2.1	100.2 ± 1.5	24.1 ± 0.9
**17**	105.8 ± 3.5	66.1 ± 3.5	94.0 ± 0.8	48.3 ± 1.0
**18**	105.9 ± 0.9	0.1 ± 0.6	102.5 ± 2.3	0.8 ± 1.0
**19**	89.6 ± 3.8	0.2 ± 1.0	89.7 ± 3.8	-0.3 ± 2.1
**20**	96.4 ± 9.1	2.5 ± 0.3	100.5 ± 4.8	-0.4 ± 1.6
**21**	107.0 ± 5.4	99.0 ± 1.9	102.6 ± 4.7	91.9 ± 8.2
**22**	98.3 ± 4.9	73.9 ± 2.2	104.5 ± 2.5	5.1 ± 1.3

REFERENCES

(1) Kolarič, A.; Novak, D.; Weiss, M.; Hrast, M.; Zdovc, I.; Anderluh, M.; Minovski, N. Cyclohexyl amide-based novel bacterial topoisomerase inhibitors with prospective GyrA-binding fragments. *Future Med. Chem.* **2019**, *11*, 935−945.

(2) Bax, B. D.; Chan, P. F.; Eggleston, D. S.; Fosberry, A.; Gentry, D. R.; Gor-rec, F.; Giordano, I.; Hann, M. M.; Hennessy, A.; Hibbs, M.; Huang, J.; Jones, E.; Jones, J.; Brown, K. K.; Lewis, C. J.; May, E. W.; Saunders, M. R.; Singh, O.; Spitzfaden, C. E.; Shen, C.; Shillings, A.; Theobald, A. J.; Wohlkonig, A.; Pearson, N. D.; Gwynn, M. N. Type IIA topoisomerase inhibition by a new class of antibacterial agents. *Nature* **2010**, *466*, 935−940.

(3) Singh, S. B.; Kaelin, D. E.; Wu, J.; Miesel, L.; Tan, C. M.; Meinke, P. T.; Ol-sen, D.; Lagrutta, A.; Bradley, P.; Lu, J.; Patel, S.; Rickert, K. W.; Smith, R. F.; Soisson, S.; Wei, C.; Fukuda, H.; Kishii, R.; Takei, M.; Fukuda, Y. Oxa-bicyclooctane-linked novel bacterial topoisomerase inhibitors as broad spectrum antibacterial agents. *ACS Med. Chem. Lett.* **2014**, *5*, 609−614.

(4) Surivet, J. P.; Zumbrunn, C.; Rueedi, G.; Bur, D.; Bruyère, T.; Locher, H.; Ritz, D.; Seiler, P.; Kohl, C.; Ertel, E. A.; Hess, P.; Gauvin, J. C.; Mirre, A.; Kaegi, V.; Dos Santos, M.; Kraemer, S.; Gaertner, M.; Delers, J. ; Ender-lin-Paput, M.; Weiss, M.; Sube, R.; Hadana, H.; Keck, W.; Hubschwerlen, C. Novel tetrahydropyran-based bacterial topoisomerase inhibitors

with potent anti-gram positive activity and improved safety profile. *J. Med. Chem.* **2015**, *58*, 927−942.

(5) Hearnshaw, S. J.; Edwards, M. J.; Stevenson, C. E.; Lawson, D. M.; Maxwell, A. A New Crystal Structure of the Bifunctional Antibiotic Simocyclinone D8 Bound to DNA Gyrase Gives Fresh Insight into the Mechanism of Inhibition. *J. Mol. Biol.* **2014**, *426*, 2023-2033.

(6) Veselkov, D.A.; Laponogov, I.; Pan, X.S.; Selvarajah, J.; Skamrova, G.B.; Branstrom, A.; Narasimhan, J.; Prasad, J.V.; Fisher, L.M.; Sanderson, M.R. Structure of a quinolone-stabilized cleavage complex of topoisomerase IV from Klebsiella pneumoniae and comparison with a related Streptococcus pneumoniae complex *Acta Cryst. D Struct. Biol.* **2016**, *72*, 488-496.

(7) Kolarič, A.; Germe, T.; Hrast, M.; Stevenson, C. E. M.; Lawson, D. M.; Burton, N. P.; Vörös, J.; Maxwell, A.; Minovski, N.; Anderluh, M. Potent DNA gyrase inhibitors bind asymmetrically to their target using symmetrical bifurcated halogen bonds. *Nat. Commun.* **2021**, *12*, 1-13.
